# Characterization of Pharmacologic and Pharmacokinetic Properties of CCX168, a Potent and Selective Orally Administered Complement 5a Receptor Inhibitor, Based on Preclinical Evaluation and Randomized Phase 1 Clinical Study

**DOI:** 10.1371/journal.pone.0164646

**Published:** 2016-10-21

**Authors:** Pirow Bekker, Daniel Dairaghi, Lisa Seitz, Manmohan Leleti, Yu Wang, Linda Ertl, Trageen Baumgart, Sarah Shugarts, Lisa Lohr, Ton Dang, Shichang Miao, Yibin Zeng, Pingchen Fan, Penglie Zhang, Daniel Johnson, Jay Powers, Juan Jaen, Israel Charo, Thomas J. Schall

**Affiliations:** 1 Department of Medical and Clinical Affairs, ChemoCentryx, Inc., 850 Maude Avenue, Mountain View, California, United States of America; 2 Department of Biology, ChemoCentryx, Inc., 850 Maude Avenue, Mountain View, California, United States of America; 3 Department of Chemistry, ChemoCentryx, Inc., 850 Maude Avenue, Mountain View, California, United States of America; 4 Department of Drug Metabolism and Pharmacokinetics, ChemoCentryx, Inc., 850 Maude Avenue, Mountain View, California, United States of America; 5 Department of Discovery and Research, ChemoCentryx, Inc., 850 Maude Avenue, Mountain View, California, United States of America; Medizinische Universitat Graz, AUSTRIA

## Abstract

The complement 5a receptor has been an attractive therapeutic target for many autoimmune and inflammatory disorders. However, development of a selective and potent C5aR antagonist has been challenging. Here we describe the characterization of CCX168 (avacopan), an orally administered selective and potent C5aR inhibitor. CCX168 blocked the C5a binding, C5a-mediated migration, calcium mobilization, and CD11b upregulation in U937 cells as well as in freshly isolated human neutrophils. CCX168 retains high potency when present in human blood. A transgenic human C5aR knock-in mouse model allowed comparison of the *in vitro* and *in vivo* efficacy of the molecule. CCX168 effectively blocked migration in *in vitro* and *ex vivo* chemotaxis assays, and it blocked the C5a-mediated neutrophil vascular endothelial margination. CCX168 was effective in migration and neutrophil margination assays in cynomolgus monkeys. This thorough *in vitro* and preclinical characterization enabled progression of CCX168 into the clinic and testing of its safety, tolerability, pharmacokinetic, and pharmacodynamic profiles in a Phase 1 clinical trial in 48 healthy volunteers. CCX168 was shown to be well tolerated across a broad dose range (1 to 100 mg) and it showed dose-dependent pharmacokinetics. An oral dose of 30 mg CCX168 given twice daily blocked the C5a-induced upregulation of CD11b in circulating neutrophils by 94% or greater throughout the entire day, demonstrating essentially complete target coverage. This dose regimen is being tested in clinical trials in patients with anti-neutrophil cytoplasmic antibody-associated vasculitis.

***Trial Registration*** ISRCTN registry with trial ID ISRCTN13564773.

## Introduction

**Fig 2:**
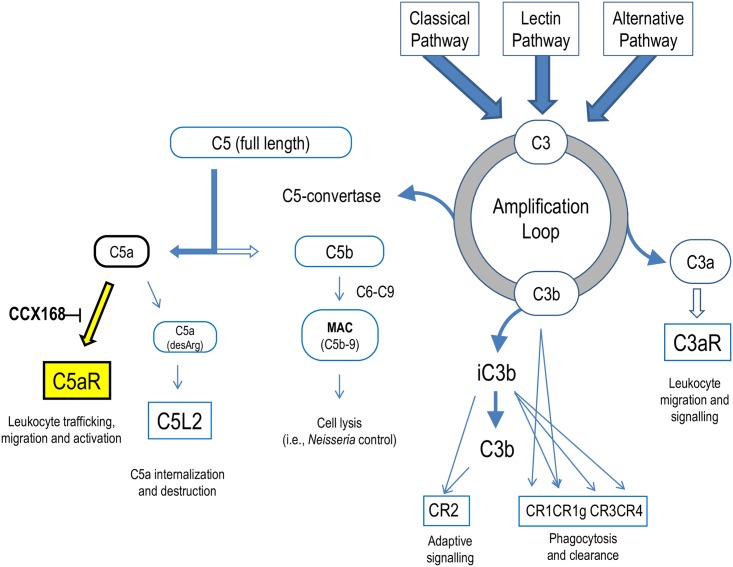
The Complement Cascade Showing the Point of Intervention of CCX168. The complement cascade can be activated by the classical, lectin, or alternative pathways and leads to the formation of C3a, C3b, C5b-9 (terminal complement complex) and C5a. During the amplification loop, full length C3 is cleaved to form C3a and C3b; C3aR is the G protein coupled expressed receptor that responds to C3a, while C3b covalently binds to foreign surfaces and aids in phagocytosis and clearance. C5 convertase is formed during the amplification loop, leading to the cleavage of full length C5 at a specific arginine-leucine bond to form C5a and C5b. C5b associates with complement components C6, C7, C8, and C9 to form the terminal complement complex or membrane attack complex (MAC), typically on the surface of pathogenic bacterial cells. C5aR (CD88) is the G protein coupled receptor expressed by innate immune cells, such as neutrophils, that responds to C5a (12 kDa), a potent pro-inflammatory mediator [2,11]. C5a rapidly induces expression of adhesion molecules on the cell surface of innate immune cells, such as neutrophils, and induces the directed migration, or chemotaxis, of these cells. C5a also mediates inflammation by stimulating vascular permeability, neutrophil degranulation, and release of lysosomal proteases and oxidative free radicals [8]. C5a is a transient molecule, being rapidly degraded of its carboxy terminal arginine [12], and hence losing about 10-fold activity on C5aR, and then being internalized and degraded via C5L2, a 7 trans-membrane receptor that has an anti-inflammatory role [13–17]. CCX168 is a small molecule antagonist of C5aR that selectively and competitively binds to this receptor.

The complement system plays a central role in generating innate and adaptive immune responses to infectious agents, foreign antigens, and tumor cells [1]. Complement 5a (C5a) is a potent pro-inflammatory mediator [2], formed by the cleavage of complement component 5. This rapidly induces surface expression of adhesion molecules and directed migration, or chemotaxis, of innate immune cells such as neutrophils (Fig 2). Inappropriate or excessive activation of the complement system, in general, and C5a formation in particular, can lead to severe inflammation and tissue destruction. Evidence of its role in various human pathologies, ranging from acute complications such as septic shock to chronic autoimmune diseases such as anti-neutrophil cytoplasmic antibody (ANCA)-associated vasculitis, atypical hemolytic uremic syndrome, systemic lupus erythematosus, rheumatoid arthritis, and ischemia/reperfusion injury [3], makes the complement system an important target for drug discovery [4–6]. Interest in targeting C5a and its receptor, C5aR, for drug development has been widespread since reports in the early 1990’s with respect to their strong inflammatory effects [7–9]. Targeting the response to C5a with a C5aR antagonist would selectively quench the inflammatory response associated with disease and cellular damage while not directly affecting the formation of the terminal complement complex or membrane attack complex C5b-9, which is known to be required for resistance to encapsulated bacterial infection, such as with *Neisseria meningitidis* [10].

Various therapeutics targeting upstream components of the complement system that showed promise in animal models have failed to perform as expected in the clinical setting, possibly because the pathways intersect with other immune, defense, and inflammation processes [4,5]. Attempts to develop small molecule therapeutics targeting C5aR resulted in drugs with poor pharmacokinetic properties, including high clearance, low oral absorption, and/or cross reactivity with the hERG potassium ion channel and cytochrome P450 enzymes. In addition, lack of potency under physiologically-relevant conditions (i.e., whole blood, primary cells, and presence of plasma proteins) and agonist properties also hindered clinical development [5,18].

CCX168 (International Nonproprietary Name avacopan) is a novel, orally administered antagonist of C5aR that is in clinical development for ANCA-associated vasculitis and other renal diseases. The efficacy of CCX168 was tested in a mouse model of ANCA-induced glomerulonephritis [19]. In this model of necrotizing and crescentic glomerulonephritis [20], when CCX168 was given orally to human C5aR knock-in mice, the induction of disease by the myeloperoxidase ANCA was suppressed, consistent with results from the genetic deletion of C5aR [21]. In contrast, genetic deletion of C5L2 had the reverse effect, resulting in more severe disease. In addition, the terminal complement complex did not play a role in disease severity, as mice deficient in a component of the complex, complement 6, responded normally to ANCA disease induction.

The goal of this series of studies was to characterize CCX168 *in vitro* and in preclinical models, and then obtain detailed pharmacokinetic and pharmacodynamic data in healthy human volunteers in a Phase 1 clinical study.

## Methods

### In vitro Potency Assessments

#### Cells and reagents

Human U937 cells (ATCC, Rockville MD) were cultured in RPMI-1640 medium (Sigma Aldrich, St. Louis, USA) supplemented with 10% fetal bovine serum (Sigma Aldrich) and with dibutyryl cAMP (0.5 mM, Sigma Aldrich) added to the cells 18 hours before use [22]. THP-1, HEK293, MOLT4, Baf3 and MDA-MB435 cells were obtained from ATCC and grown according to their recommendations. L1.2 cells were licensed from Dr. Eugene Butcher (Stanford University, Stanford, CA). Activated human T lymphocytes were cultured as described [23–25]. All blood was collected into EDTA as an anti-coagulant. Human whole blood was collected from healthy volunteers and used within two hours. Neutrophils were isolated from human whole blood using standard density gradient separation methods. Cynomolgus monkey whole blood was from the California National Primate Research Center (Davis, CA) and was used within four hours of collection. For *in vivo* assays, CCX168 was formulated in PEG-400/solutol-HS-15 (70:30, Sigma-Aldrich, St. Louis, MO and BASF, Ludwigshafen, Germany), human C5a and all chemokines were obtained from R&D Systems (Minneapolis, MN), [^125^I]-C5a was from PerkinElmer (Boston, MA) and plasma (human and mouse) and synovial fluids (from individuals with rheumatoid arthritis or osteoarthritis) were purchased (Bioreclamation, Hicksville, NY). Alpha-1-acid glycoprotein was from Sigma-Aldrich. Indo-1AM and CyQuant were from Life Technologies (Grand Island, NY). Anti-CD11b and anti-C5aR monoclonal antibodies were from BD Biosciences (San Jose, CA). Anti-CD11b (human and mouse) antibody human clone number ICRF44 and mouse clone number M1/70 were used. Anti-C5aR (human) monoclonal antibodies were from BD Biosciences (San Jose, CA): Clone number 20/70. Catalogue numbers for the cell lines used are as follows: U937: ATCC-CRL-1593.2; THP-1: ATCC- TIB-202; HEK293: ATCC-CRL-3216; MOLT4: ATCC CRL-1582; Baf3: R&D Systems with paying licensing fee; MDA-MB435: ATCC- HTB-129.

#### *In vitro* assays

Chemotaxis, calcium mobilization, and radioligand binding assays were conducted as previously described [23–25]. The respiratory burst assay was conducted as described [26].

#### Data analysis

The ability of CCX168 to affect C5a-mediated migration was determined by quantifying the extent of the rightward shift in the concentration curves. This was expressed as an “A” value. For example, an A_2_ value indicates the concentration of CCX168 that results in a two-fold rightward shift of the dose-response curve for a C5a-mediated effect, and correlates with 50% receptor occupancy by a competitive antagonist such as CCX168. Potency calculations (A_2_) from functional assays were made as described [24] using the following equation:

$$pA_{2}=\;p\left[drug\left(M\right)\right]\;–\;p\left[\left(A’/A\right)-1\right]$$

where A reflects the potency of the agonist in the absence of antagonist and A’ reflects the potency of the agonist in the presence of antagonist at a given concentration of drug ([drug(M)]). The potency values (A and A’) for the CD11b upregulation and calcium mobilization assays were calculated using nonlinear regression with a one-site competition model, equation Y = Bottom + (Top-Bottom)/(1+10^(X-LogIC_50_)), with GraphPad Prism Software (San Diego, CA), as were reported inhibition values (IC_50_).

Statistical testing for significance of the test group results compared to the vehicle control group was performed. The data were inspected visually to determine if the distribution was substantially normal. Since the data were found to be normally distributed, Student’s t-test was applied and non-parametric testing was not performed.

### Animal models

All animal studies were approved by the institutional animal care and use committee of ChemoCentryx and in accordance with the *Guide for the Care and Use of Laboratory Animals* of the National Research Council. For retro-orbital blood draws, an ophthalmic analgesic solution (tetracaine hydrochloride 0.5%) was applied ~1–5 minutes prior to blood draws and each orbital sinus was only used once to minimize any suffering associated with the procedure. Mice were humanely sacrificed by cervical dislocation under CO_2_ in accordance with current American Veterinary Medical Association's Guidelines on Euthanasia.

#### C5a-induced leukopenia in human C5aR knock-in mice

Human C5aR knock-in mice were dosed with vehicle (PEG-400/solutol-HS15 70:30, 5 mL/kg) or CCX168 by oral gavage. One hour after dosing, C5a (20 μg/kg, 0.1 mL dose volume) was injected intravenously and blood samples collected from retro-orbital eye bleeds. Blood leukocyte levels were analyzed by flow cytometry.

#### C5a-induced neutropenia in cynomolgus monkeys

All experiments performed in cynomolgus monkeys were performed at Covance Research Products (Alice, TX) with the approval of Covance Research Products Animal Care and Use Committee and in compliance with the *Guide for the Care and Use of Laboratory Animals* essentially as previously described [27].

### Phase 1 Clinical Trial in Healthy Volunteers

#### Objectives and study design

The primary objective of this clinical trial was to evaluate the safety and tolerability of CCX168 in humans. Secondary objectives included the characterization of the pharmacokinetic and pharmacodynamic profiles of CCX168.

This was a randomized, double-blind, placebo-controlled, single-ascending-dose and multiple-ascending-dose Phase 1 study (Covance Phase 1 unit, Switzerland). All subjects were to be in good general health prior to enrolling, and gave written informed consent to participate before any study procedures were initiated. The study was performed in accordance with the Declaration of Helsinki principles, Good Clinical Practice guidelines, and the International Conference on Harmonisation guidelines. The study protocol was approved by the Ethics Committee on 22 October 2009 and the Health Authority on 15 December 2009, prior to subject enrollment. The first subject was screened for the study on 21 December 2009 and the last subject completed the study on 29 September 2010. The study was not registered in a clinical trials registry prior to study start because it is a Phase 1 study which does not require registration in such a registry. The authors confirm that all ongoing and related trials for this drug/intervention are registered.

Forty healthy subjects (n = 8 per cohort) were randomized in a 3:1 ratio in five sequential cohorts to receive either a single dose of CCX168 (n = 6) or placebo (n = 2) in Period 1 or multiple doses for 7 days in Period 2, except for the first cohort (CCX168 n = 5; placebo n = 3). The unequal randomization was selected to ensure that the majority of subjects receive CCX168 and not placebo, which is customary for a Phase 1 study. The CCX168 single doses tested were 1, 3, 10, 30, and 100 mg and the CCX168 multiple doses tested were 1 mg once daily (q.d.), 3 mg q.d., 10 mg q.d., 30 mg twice daily (b.i.d.), and 50 mg b.i.d. Subjects were followed for 1 week after the single dose and 3 weeks after completing the 7-day treatment period. There were at least 2 weeks between the single-dose and multiple-dose study periods, and the multiple-dose period was not started until the single-dose period for 1, 3, and 10 mg CCX168 had been completed. The single doses in Period 1 were given as a solution to all subjects in Period 1 and to all subjects in the first three cohorts of Period 2. In order to blind subjects to the study drug, matching volumes of dosing solution without CCX168 were given to the placebo subjects, and the dosing solution was provided in opaque containers. For the 30 mg and 50 mg twice daily dosing cohorts in Period 2, CCX168 or placebo were administered as gelatin capsules containing 10 mg CCX168. The CCX168 and placebo capsules were identical in appearance. Only the study pharmacist at the study site had access to the treatment randomization code.

The sample size determination was based on practical and not statistical considerations. Subjects were assigned to receive CCX168 or placebo based on a randomization list generated by the study pharmacist prior to start of dosing.

Regarding the primary assessment, safety and tolerability, the subject incidence of adverse events was summarized. Summary statistics were calculated on the safety laboratory parameters, and the CCX168 pharmacokinetic and pharmacodynamics parameters. No inferential statistical testing was performed on any study variables.

The study was funded by ChemoCentryx, Inc.

#### Safety and tolerability assessments

Standard clinical methods were used to assess safety and tolerability, including physical examinations, vital signs, 12-lead electrocardiogram, and clinical laboratory measurements, throughout the study. Adverse events were recorded over the course of the study. Investigators assessed all adverse events for severity, duration, outcome, and possible relationship to the study medication. Adverse event severity was assessed based on the Common Terminology Criteria for Adverse Events version 4.0.

#### Pharmacokinetic assessment

Blood samples (6 mL) were collected at predetermined time points. After centrifuging at 2,000*g* for 10 min, plasma was stored at -80°C until analysis. After protein precipitation using acetonitrile, supernatant solutions were analyzed by high-performance liquid chromatography-tandem mass spectrometry using a validated method (nominal plasma concentration range: 0.2–100 ng/mL). PK values were generated using non-compartmental analysis with WinNonlin Phoenix, version 5.2 (Pharsight, Mountain View, CA).

#### Pharmacodynamic assessment

Peripheral blood samples from selected Phase 1 cohorts were collected at the indicated times; 0.1-mL aliquots were mixed with C5a over a range of concentrations and incubated at 37°C for 30 min, after which the blood was cooled on wet ice and a monoclonal antibody specific for CD11b (BD Biosciences) was added. After 30 minutes on ice, red blood cells were lysed using ice-cold FACS Lysing Solution (BD Biosciences), and the leukocytes preserved in fixative. Using flow cytometry, neutrophils were identified by their forward/side-scatter properties and the mean fluorescence intensity (MFI) of anti-CD11b staining on these cells was measured.

For the single-dose period, the effect of CCX168 treatment on C5a-induced upregulation of CD11b was assessed at 2 and 24 hours after a single dose of 10 mg CCX168, at 2 and 12 hours after a single dose of 30 mg and 100 mg CCX168, and at 2 and 12 hours on Day 7 of the multiple-dose period at 30 mg CCX168 given twice daily. The reason for shifting from 24 to 12 hours after dosing was that it was determined, based on the early PK results, that a twice daily dosing regimen would be more appropriate than a once daily regimen.

## Results

### In vitro Assessment of CCX168 Potency and Selectivity

**Fig 3:**
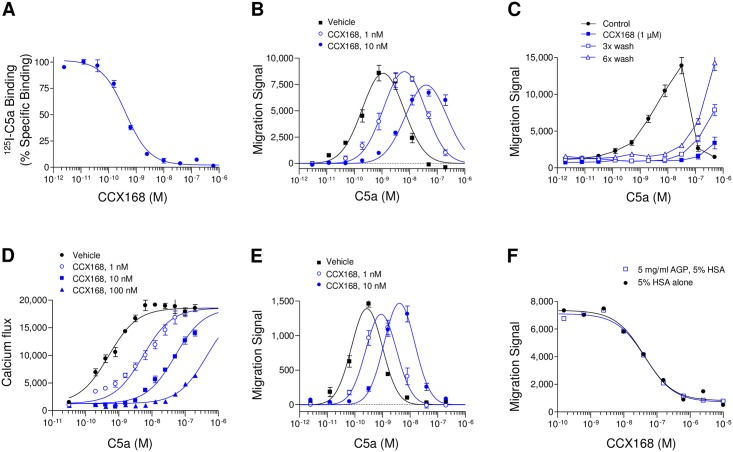
*In vitro* Characterization of CCX168 Using C5aR-Expressing U937 Cells. (A) CCX168 inhibition of [^125^I]-C5a binding to U937 cells with a potency (IC_50_ value) of 0.1 nM; each data point is the mean of 4 replicates ± standard error; study repeated 5 separate times, representative experiment shown. (B) Effects of vehicle control (■) and 1 nM (○) or 10 nM (●) CCX168 on C5a-mediated chemotaxis of U937 cells in buffer; each data point is the mean of 8 replicates ± standard error; study repeated 5 separate times, representative experiment shown. (C) C5a-mediated chemotaxis of U937 cells in the absence (●) or presence (■) of 1 μM CCX168, as well as following 1 μM CCX168 / 3x wash (□) and 1 μM CCX168 / 6x wash (Δ) treatments; each data point is the mean of 8 replicates ± standard error; study repeated 2 separate times, representative experiment shown. (D) C5a-induced intracellular calcium release in U937 cells, as measured by FLIPR, in the presence of vehicle control (●) and various concentrations of CCX168, 1 nM (○), 10 nM (■), or 100 nM (▲); each data point is the mean of 4 replicates ± standard error; study repeated 2 separate times, representative experiment shown. (E) C5a-mediated chemotaxis of U937 cells in 100% human plasma in the presence of vehicle control (■) and 1 nM (○) or 10 nM (●) CCX168; each data point is the mean of 8 replicates ± standard error; study repeated 2 separate times, representative experiment shown. (F) Inhibition by CCX168 of U937 cell chemotaxis towards 0.1 nM C5a in the presence (□) or absence (●) of α1-acid glycoprotein (AGP, 5 mg/mL) in buffer containing 5% human serum albumin (HSA); each data point is the mean of 8 replicates ± standard error; study repeated 2 separate times, representative experiment shown.

CCX168 displaced [^125^I]-C5a binding to C5aR on a human myeloid cell line (U937) with a potency (IC_50_ value) of 0.1 nM (Fig 3A). Two measures of CCX168 potency were used, the IC_50_ (50% inhibition of a half-maximal agonist concentration), and the dose ratio or A_2_ (the concentration of CCX168 that produces a 2-fold right-shift in C5a activity) (Fig 3B and 3C). CCX168 inhibits C5a-mediated chemotaxis of U937 cells with a potency (A_2_) of 0.2 nM. Addition of CCX168 to U937 cells in a calcium mobilization assay inhibited C5a with a potency (A_2_) of 0.1 nM (Fig 3D). CCX168 inhibits chemotaxis of U937 cells in 100% human plasma (Fig 3E) with no loss of effect in the presence of α1-acid glycoprotein (Fig 3F). Furthermore, CCX168 did not display any agonist activities with any of the assays used (cytoplasmic calcium flux, chemotaxis, or CD11b upregulation). CCX168 is selective for C5aR, with no activity (IC_50_ >5,000 nM) measured with the C5aR-related receptors C5L2, C3aR, ChemR23, GPR1, and FPR1, a panel of 18 of the chemokine receptors, a panel of 54 pharmacologically relevant receptors, and the cytochrome P450 enzymes 1A2, 2C9, 2C19, 2D6, 3A4 (details in S1 and S2 Tables). In addition, CCX168 did not inhibit the hERG potassium ion channel as measured in a patch clamp assay (IC_50_ <5000 nM).

**Fig 4:**
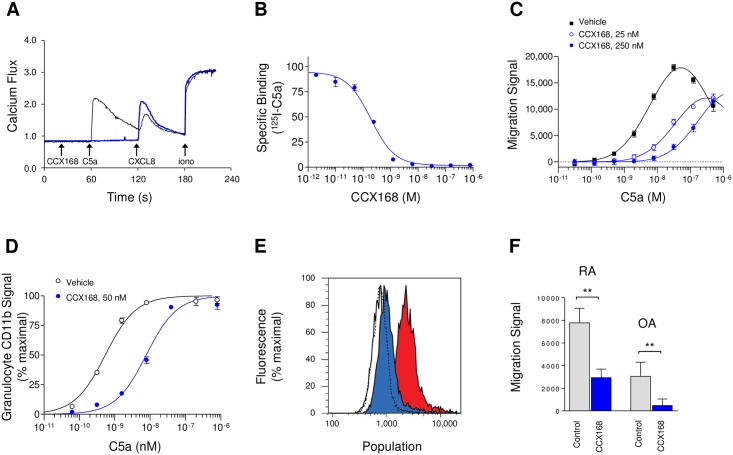
*In vitro* Characterization of CCX168 Using Freshly-Isolated Human Neutrophils or Human Whole Blood. (A) Sequential intracellular calcium release in human neutrophils in response to C5a (100 nM), CXCL8 (100 nM) or ionomycin (1 μg/mL), in the presence (blue line) or absence (black line) of CCX168 (10 μM). CCX168 blocked calcium release induced by C5a but not by the CXCR1 ligand CXCL8. (B) Binding of [^125^I]-C5a to human neutrophils in the presence of a range of concentrations of CCX168. CCX168 inhibited [^125^I]-C5a binding with a potency (IC_50_ value) of 0.2 nM. Each data point represents the mean of 4 replicates ± standard error, and the experiment was repeated 2 separate times. (C) C5a-induced chemotaxis of leukocytes in human whole blood, in the presence of vehicle control (■), and 25 nM (○) or 250 nM (●) CCX168. CCX168 inhibited leukocyte chemotaxis in a dose-dependent manner. Each data point represents the mean of 8 replicates ± standard error, and the experiment was repeated 5 separate times. (D) C5a-induced upregulation of CD11b on the surface of neutrophils in human whole blood in the presence of vehicle control (○) or 50 nM CCX168 (●). CCX168 inhibited CD11b upregulation. Each data point represents the mean of 4 replicates ± standard error, and the experiment was repeated 4 separate times. (E) C5a-induced oxidative burst in isolated human neutrophils in the presence of vehicle control (red histogram) or CCX168 (100 nM, blue histogram). The empty histograms represent untreated neutrophils (i.e., no C5a) in the presence of vehicle control (solid black line) or CCX168 (dotted black line). CCX168 blocked the C5a-induced oxidative burst but did not affect untreated neutrophils. The experiment was repeated 3 times. (F) Chemotaxis of leukocytes in human whole blood towards synovial fluid in the presence of vehicle control or CCX168 (100 nM). Experiments using synovial fluid from a patient with rheumatoid arthritis (RA) or osteoarthritis (OA) are shown. CCX168 inhibited leukocyte chemotaxis induced by each of these samples. Each bar represents the mean of 8 replicates ± standard error, and the experiment was repeated two times. **p<0.01 based on Student’s t-test.

CCX168 competitively and selectively blocked C5a-induced calcium mobilization in purified human neutrophils, with an IC_50_ value of 0.2 nM (Fig 4A). Similar results were obtained using purified human blood monocytes. CCX168 also inhibited binding of [^125^I]-C5a to C5aR on human neutrophils with an IC_50_ of 0.2 nM (Fig 4B). Moreover, CCX168 inhibited C5a-mediated chemotaxis of neutrophils in freshly-collected human blood with an A_2_ of 1.7 nM (Fig 4C). CCX168 inhibited C5a-induced increases in the level of integrin CD11b on neutrophils in human whole blood with an A_2_ of 3.0 nM (Fig 4D). In addition, CCX168 inhibited C5a-induced release of reactive-oxygen species from isolated neutrophils, and was able to completely block respiratory burst in these neutrophils (Fig 4E). Synovial fluid samples taken from subjects with rheumatoid arthritis (RA) or osteoarthritis (OA) induced chemotaxis of human blood leukocytes [24]; CCX168 significantly reduced this response, suggesting that both types of synovial samples contain active C5a (Fig 4F).

### Activity of CCX168 in Transgenic Human C5aR Knock-in Mice

**Fig 5:**
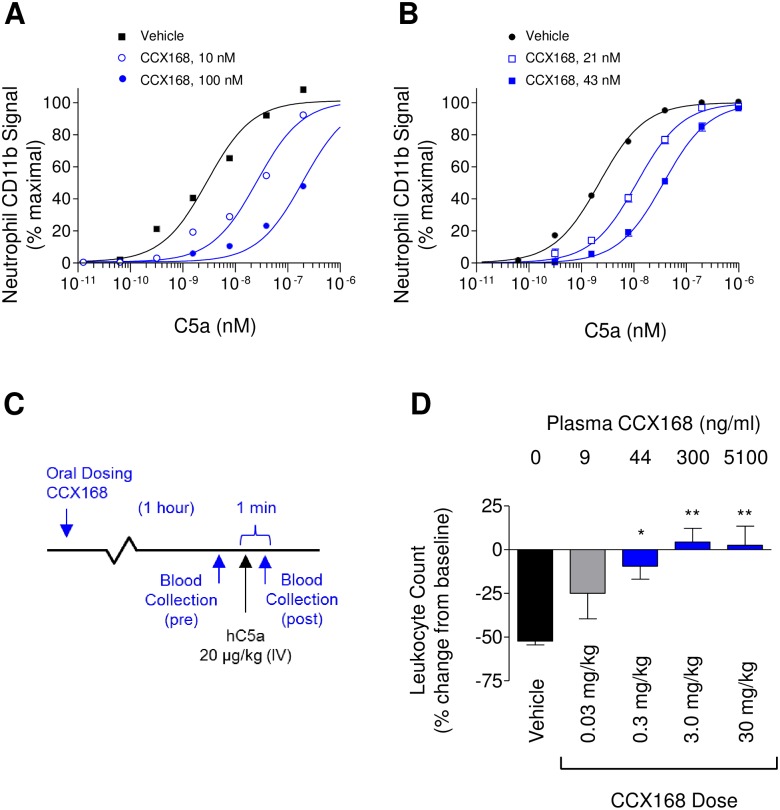
Biological Effects of CCX168 on Transgenic Human C5aR Knock-in Mice. (A) C5a-induced upregulation of CD11b on the surface of neutrophils in whole blood from human C5aR knock-in mice, after addition of vehicle (■), 10 nM (○), or 100 nM (●) CCX168. CCX168 inhibited CD11b upregulation in a dose-dependent manner. Each data point represents the mean of 3 replicates ± standard error; the study was repeated 3 separate times. (B) C5a-induced upregulation of CD11b on the surface of neutrophils in whole blood from human C5aR knock-in mice 1 hour after oral dosing with vehicle (●), 0.075 mg/kg (□), or 0.15 mg/kg (■) CCX168. Neutrophil CD11b upregulation was diminished in blood from CCX168-treated mice. Plasma concentrations of CCX168 are indicated. (C) Schematic of the C5a-induced leukopenia study in human C5aR knock-in mice. One hour following oral administration of CCX168, C5a (20 μg/kg) was administered intravenously, with blood drawn immediately before and 1 minute after C5a injection. Blood samples were analyzed for leukocyte numbers. (D) Effect of CCX168 in the C5a-induced leukopenia study in hC5aR knock-in mice. The percent change in the number of leukocytes in the blood sample collected after C5a injection, relative to the sample collected prior to C5a injection, is shown for each group (4 mice per group). Above each bar is the average concentration of CCX168 in the pre-injection blood samples. CCX168 inhibited the depletion of blood leukocytes caused by intravenous administration of C5a. *p<0.05, **p<0.01 based on Student’s t-test.

CCX168 displays reduced activity on C5aR from most model species, including mice, rats, and rabbits. Therefore, we generated a transgenic mouse strain in which the mouse C5aR coding region was replaced with the human C5aR coding region, and the innate immune cells of these human C5aR knock-in mice express human C5aR and respond normally to mouse or human C5a [19]. The antagonism by CCX168 of the transgenic immune cells was demonstrated by multiple methods. *In vitro*, CCX168 potently (A_2_ value of 1.4 nM) blocked C5a-induced CD11b upregulation on neutrophils from human C5aR knock-in mice when CCX168 was added to freshly-collected blood from these mice (Fig 5A). *Ex vivo*, oral dosing of CCX168 to the human C5aR knock-in mice potently inhibited subsequent C5a-induced CD11b upregulation on neutrophils from freshly collected blood samples. The neutrophils from CCX168-treated mice were again much less responsive to C5a compared to vehicle-treated mice, with CCX168 exhibiting an A_2_ value of 3.3 nM (Fig 5B). An *in vivo* assay was conducted wherein CCX168 or vehicle was dosed orally to the hC5aR knock-in mice and, one hour later, human C5a was injected intravenously (Fig 5C). Precisely one minute after C5a injection, the number of circulating blood leukocytes was counted and compared to the reading just before the C5a injection. In vehicle-treated mice, the circulating blood leukocyte count was reduced by 53% relative to pre-C5a treatment (Fig 5D). In mice dosed orally with 0.03 mg/kg of CCX168, the resulting plasma CCX168 concentration of 15 nM (8.7 ng/mL) reduced the drop in circulating leukocytes from 53% to 25% (Fig 5D). In mice administered 0.3 mg/kg of CCX168, the resulting plasma CCX168 concentration of 75 nM (44 ng/mL) reduced the drop in circulating leukocytes from 53% to only 10% relative to baseline (p<0.05 for CCX168 vs. vehicle control). Oral doses of CCX168 of either 3 or 30 mg/kg completely blocked C5a-induced leukopenia in hC5aR knock-in mice (p<0.01 for CCX168 compared with vehicle control).

### Activity of CCX168 in Cynomolgus Monkeys

**Fig 6:**
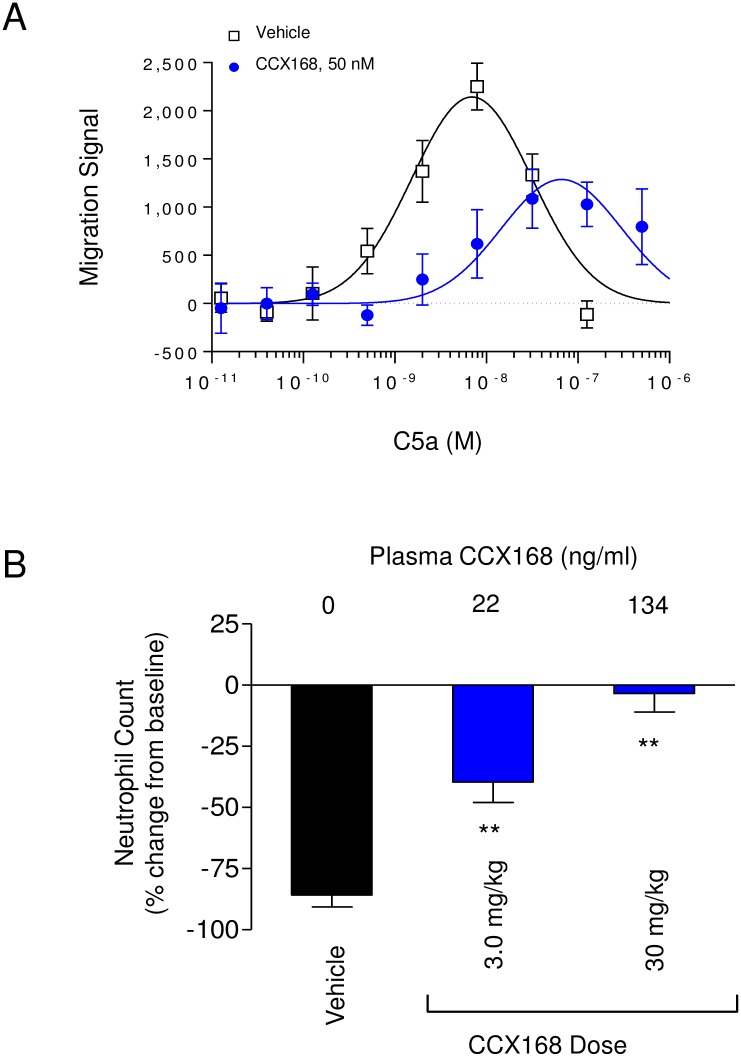
Biological Effects of CCX168 in Cynomolgus Monkeys. (A) *In vitro* C5a-induced chemotaxis of freshly-isolated neutrophils from cynomolgus monkeys, conducted in 100% cynomolgus plasma containing vehicle (□) or 50 nM CCX168 (●). CCX168 inhibited neutrophil chemotaxis (A_2_ = 3.0 nM). Each data point represents the mean of 8 replicates ± standard error, and the study was repeated 2 separate times. (B) *In vivo* effect of CCX168 in the C5a-induced neutropenia model in monkeys. The percent change in the number of neutrophils in the blood collected after C5a injection, relative to the sample collected prior to C5a injection, is shown (4 monkeys per group). Above each bar is the average concentration of CCX168 in the pre-injection blood samples. CCX168 inhibited, in a dose-dependent manner, the depletion of blood neutrophils caused by intravenous administration of C5a. **p<0.01 based on Student’s t-test.

CCX168 potently inhibited cynomolgus monkey C5aR in a chemotaxis assay with freshly-isolated cynomolgus blood neutrophils in 100% plasma (A_2_ = 3.0 nM; Fig 6A). Oral administration of CCX168 to cynomolgus monkeys resulted in sustained plasma concentrations (t_½_ = 6.0 hours at 100 mg/kg). A C5a-induced neutropenia model was established similar to the one described in Fig 5C, wherein intravenous injection of C5a in vehicle-treated monkeys led to an 80% decrease in circulating blood neutrophil numbers, measured 1 minute after C5a injection. In monkeys pretreated with an oral 3 mg/kg dose of CCX168, the resulting plasma concentration of 38 nM (22 ng/mL) CCX168 inhibited the C5a-induced reduction in blood neutrophils from 80% to 35% relative to baseline (Fig 6B, p<0.01 for CCX168 vs. vehicle control). With a 30 mg/kg oral dose of CCX168, the 230 nM (134 ng/mL) plasma CCX168 concentration completely blocked subsequent C5a-induced neutropenia (Fig 6B; p<0.01 for CCX168 vs. vehicle control).

### Phase 1 Clinical Trial

#### Subject disposition and characteristics

**Fig 1:**
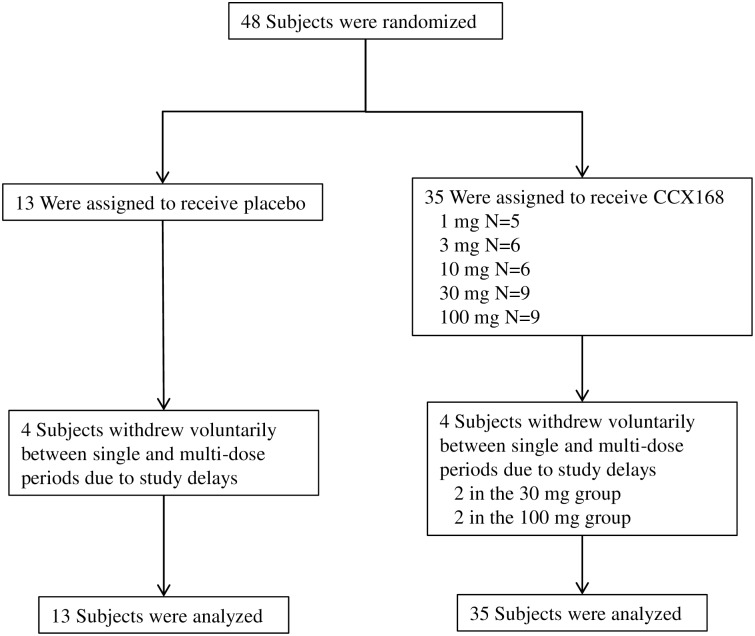
Subject Disposition for Phase 1 Clinical Study.

A total of 48 subjects were enrolled, 24 men and 24 women. The subject disposition is shown in Fig 1. Thirteen subjects received placebo and 35 received CCX168. Eight subjects withdrew voluntarily due to study delays between the single-dose and multi-dose periods of the study. However, their available data were included in the analyses. The baseline demographics and characteristics for the single-dose period are summarized in Table 1 and for the multiple-dose period in Table 2. The mean age was 38 (range 21 to 45) years. In each period, 21 (52.5%) of the 40 subjects were male and 19 (47.5%) were female. Forty seven subjects were Caucasian and one was Asian. There were no withdrawals from the study due to adverse events.

**Table 1:** Demographic Data for Subjects in Period 1 of the Phase 1 Clinical Study (Mean, Min—Max).

Treatment	Mean Age [years] (range)	Mean Height [cm] (range)	Mean Weight [kg] (range)	Mean Body Mass Index [kg/m^2^] (range)
**Placebo (N = 11)**	38.9 (29–44)	173.1 (166–189)	70.49 (57.0–84.9)	23.50 (20.0–26.7)
**1 mg CCX168 (N = 5)**	38.6 (31–44)	178.4 (157–188)	76.90 (67.3–96.0)	24.25 (20.3–27.3)
**3 mg CCX168 (N = 6)**	41.7 (36–44)	174.5 (163–188)	74.22 (61.1–88.7)	24.39 (20.7–27.4)
**10 mg CCX168 (N = 6)**	33.3 (24–44)	173.2 (159–190)	73.12 (56.2–91.5)	24.15 (20.2–26.1)
**30 mg CCX168 (N = 6)**	32.8 (26–43)	172.7 (159–193)	69.77 (56.5–82.1)	23.46 (19.6–26.5)
**100 mg CCX168 (N = 6)**	40.3 (34–43)	168.3 (153–183)	62.48 (54.4–73.6)	22.08 (19.9–25.0)

**Table 2:** Demographic Data for Subjects in Period 2 of the Phase 1 Clinical Study (Mean, Min—Max).

Treatment	Mean Age [years] (range)	Mean Height [cm] (range)	Mean Weight [kg] (range)	Mean Body Mass Index [kg/m^2^] (range)
**Placebo (N = 11)**	40.1 (35–44)	170.6 (153–191)	70.46 (54.4–101.5)	24.15 (20.0–29.3)
**1 mg CCX168 once daily (N = 5)**	38.6 (31–44)	178.4 (157–188)	76.90 (67.3–96.0)	24.25 (20.3–27.3)
**3 mg CCX168 once daily (N = 6)**	41.7 (36–44)	174.5 (163–188)	74.22 (61.1–88.7)	24.39 (20.7–27.4)
**10 mg CCX168 once daily (N = 6)**	33.3 (24–44)	173.2 (159–190)	73.12 (56.2–91.5)	24.15 (20.2–26.1)
**30 mg CCX168 twice daily (N = 6)**	38.2 (29–44)	174.8 (159–193)	68.75 (55.5–82.1)	22.46 (19.6–26.5)
**50 mg CCX168 twice daily (N = 6)**	37.5 (21–45)	167.3 (160–183)	59.93 (49.8–67.1)	21.45 (18.5–25.0)

#### Safety and tolerability of CCX168 in humans

CCX168 was well tolerated. No dose-limiting adverse events and no serious adverse events were reported or observed at any dose level or dosing regimen used. No apparent dose response was observed in incidence of adverse events. The most common adverse events reported in subjects receiving CCX168 was headache, reported in 21% of subjects receiving CCX168 compared to 18% in the placebo group. Diarrhea (7% vs. 9% in placebo), dizziness (7% vs. 0% in placebo), lower abdominal pain (7% vs. 0% in placebo), nausea (7% vs. 0% in placebo), and oropharyngeal pain (7% vs. 9% in placebo) were the other more commonly observed adverse events. There was not a dose dependency regarding the incidence of these adverse events. No laboratory, ECG, or vital signs abnormalities related to CCX168 use occurred.

#### Pharmacokinetic profile

**Fig 7:**
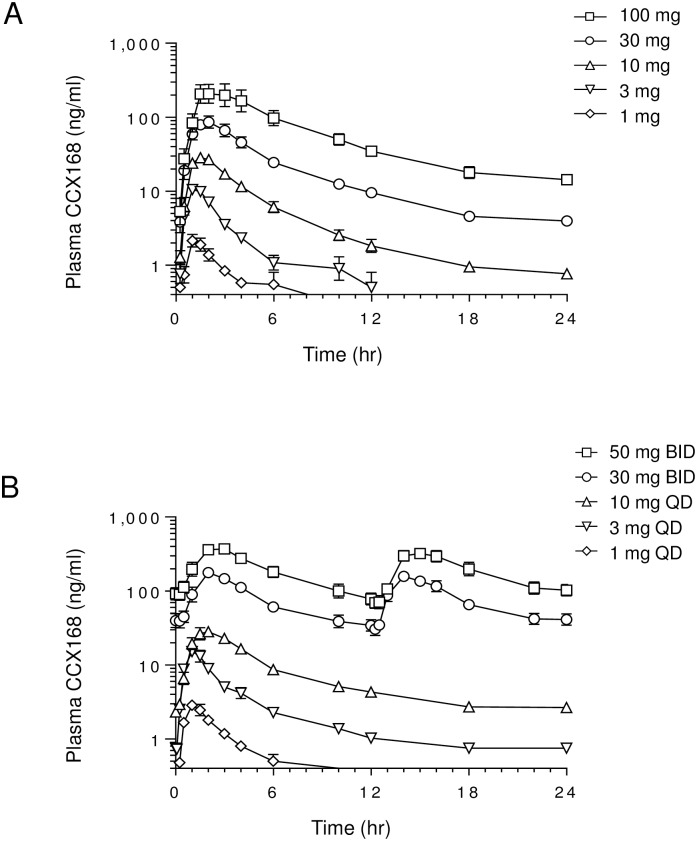
Pharmacokinetic Profile of CCX168 in Healthy Human Volunteers. Plasma concentration versus time profiles of CCX168 after (A) single oral doses of 1 mg (◊), 3 mg (∇), 10 mg (Δ), 30 mg (○), or 100 mg (□) CCX168 and (B) after receiving CCX168 once daily at 1 mg (◊), 3 mg (∇), or 10 mg (Δ), or twice daily at 30 mg (○) or 50 mg (□) for 7 days. The data points represent the mean ± standard error.

Following oral administration, CCX168 was absorbed rapidly and reached peak plasma levels after 1 to 2 hours (Table 3 and Fig 7A). After reaching the peak plasma level, CCX168 showed a biphasic elimination profile, as observed from the log-linear concentration-time profiles (Fig 7A), with a rapid early phase followed by a long terminal phase, a moderate to high apparent clearance in humans, and a large apparent volume of distribution. Despite the long observed terminal half-life, repeat administration for seven days resulted in only modest accumulation at the higher dose levels. Steady state was reached after 3 to 4 days of oral dosing.

**Table 3:** Summary of Pharmacokinetic Parameters for CCX168 after Oral Administration to Healthy Human Volunteers.

Parameter	Single Dose Groups					Multiple Dose Groups on Day 7				
	1 mg (n = 5)	3 mg (n = 6)	10 mg (n = 6)	30 mg (n = 6)	100 mg (n = 6)	1 mg q.d. (n = 5)	3 mg q.d. (n = 6)	10 mg q.d. (n = 6)	30 mg b.i.d. (n = 6)	100 mg b.i.d. (n = 6)
**C**_**max**_ **(ng/mL)**	2 (0.9)	9 (2)	25 (6)	79 (36)	197 (157)	3 (1)	14 (4)	31 (9)	191 (60)	359 (139)
**Tmax (hr)**	1.1 (0.2)	1.2 (0.3)	1.7 (0.3)	1.7 (0.4)	2.5 (1.8)	1.0 (0)	1.2 (0.3)	1.9 (0.6)	2.2 (1.0)	2.8 (1.0)
**λ**_**z**_ **(hr**^**-1**^**)**	0.37 (0.10)	0.44 (0.18)	0.03 (0.02)	0.01 (0.01)	0.01 (0.004)	0.17 (0.19)	0.02 (0.02)	0.005 (0.002)	0.006 (0.001)	0.006 (0.001)
**t**_**1/2**_ **(hr)**	2.0 (0.7)	1.9 (1.0)	22.9 (7.3)	71.8 (32.8)	64.0 (22.1)	12.2 (13.7)	71.8 (40.2)	162 (74.7)	129 (30.7)	120 (19.5)
**CL/F (L/hr)**	195 (80)	131 (39)	87 (41)	52 (15)	62 (34)	83 (51)	34 (22)	17 (7)	6 (2)	4 (1)
**V**_**z**_**/F (L)**	518 (131)	324 (100)	2,620 (722)	4,990 (2,070)	5,260 (2,370)	822 (421)	2,540 (709)	3,520 (897)	1,080 (436)	695 (165)
**AUC**_**0-t**_ **(ng•hr/mL)**	5 (3)	23 (9)	122 (38)	557 (149)	1,880 (999)	13.2 (9)	93 (50)	415 (125)	3,770 (1,180)	9,020 (2,570)
**AUC**_**inf**_ **(ng•hr/mL)**	6 (3)	25 (10)	130 (39)	628 (199)	2,030 (1,070)	17 (12)	119 (61)	672 (310)	5,710 (2,050)	13,000 (3,120)

Values shown are geometric mean values, with standard deviations in parentheses. AUC, area under the curve; b.i.d., twice daily; CL/F, apparent oral clearance; C_max_, peak plasma concentration; q.d., once daily; t_1/2_, half-life; T_max_, time to achieve C_max_; V_z_/F, apparent volume of distribution; λ_z_ terminal phase rate constant.

In the single-ascending-dose period of the study, CCX168 displayed a linear dose-exposure profile across the 1 to 100 mg dose range (Table 3 and Fig 7A). Mean maximal plasma levels of 197 ng/mL (340 nM) were reached after a single 100 mg dose (Fig 7A), after which the terminal plasma CCX168 half-life was 64 hours. In the multiple-ascending-dose period, average CCX168 plasma concentration versus time curves on day 7 of dosing are shown in Fig 7B. Mean maximal plasma levels of 191 ng/mL (328 nM) were reached on day 7 after twice daily administration of 30 mg CCX168 (Fig 7B), after which the terminal plasma CCX168 half-life was 129 hours. The steady state mean trough concentration (at 12 hours after dosing) was 36 ng/mL (61 nM) on day 7 with 30 mg CCX168 twice daily.

#### Pharmacodynamic profile

**Fig 8:**
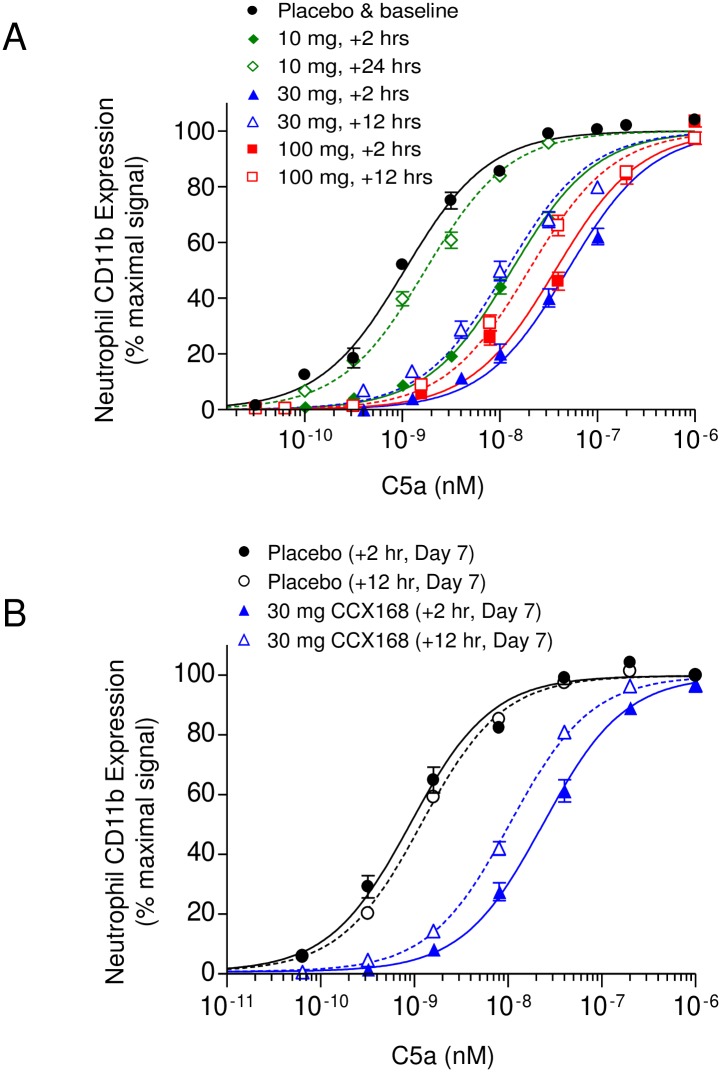
Pharmacodynamic Profile of CCX168 in Healthy Human Volunteers. Pharmacologic inhibition of C5aR by CCX168 as measured with an on-site, whole-blood *ex vivo* assay of C5a activity. (A) C5a-induced upregulation of CD11b on the surface of neutrophils in whole blood collected in the placebo group (●), and at 2 hours (♦) and 24 hours (◊) after a single dose of 10 mg CCX168, at 2 hours (▲) and 12 hours (Δ) after a single dose of 30 mg CCX168, and 2 hours (■) and 12 hours (□) after a single dose of 100 mg CCX168. CD11b upregulation was diminished in blood from subjects dosed with CCX168. (B) C5a-induced upregulation of CD11b on the surface of neutrophils in whole blood collected at 2 hours (●) or 12 hours (○) after the last dose of a 7-day regimen of placebo, or 2 hours (▲) or 12 hours (Δ) after the last dose of a 7-day regimen of 30 mg CCX168 given twice daily. CD11b upregulation was diminished in both blood samples from CCX168 subjects with a 10-fold decrease in C5a potency exhibited in the 12-hour samples.

The whole-blood *ex vivo* assay, which is based on inhibition of C5a-induced CD11b expression, was used to assess neutrophil C5aR coverage in the healthy human volunteers. The greater the inhibition of C5aR by CCX168 in the subjects’ bloodstream, the less responsive their neutrophils are to C5a, and therefore C5a potency is reduced (Fig 8A). Two hours after a 30 mg dose of CCX168, neutrophil C5aR was markedly blocked with a 20-fold rightward shift, whereas no blockade was observed in subjects receiving placebo (Fig 8B). This effect was maintained 12 hours post-dose, when the average extent of C5aR blockade was a 10-fold rightward shift. This high level of C5aR blockade observed at 2 and 12 hours post-dose is consistent with expectations based on the potency and average plasma CCX168 concentrations at the two time points (Fig 7A). In the single-ascending-dose period, C5a exhibited an EC_50_ of 1.2 nM in the absence of CCX168, and this C5a potency was reduced in a dose-dependent manner in the presence of CCX168 with an A_2_ value of 4.8 nM (Fig 8A). The potency of C5a-induced CD11b on blood neutrophils in the multiple-ascending-dose period was also inhibited after seven days of administration of 30 mg CCX168 twice daily but not placebo (Fig 8B).

## Discussion & Conclusions

Inappropriate or excessive activation of C5a formation is implicated in several human diseases [3], and blocking neutrophil trafficking by C5a has therefore been identified as a promising therapeutic strategy. Selectively blocking the action of C5a on its receptor, without affecting other parts of the complement cascade, would be desirable, because blocking the formation of the membrane attack complex could interfere with defense against pathogenic bacteria such as *Neisseria meningitidis* [10].

Attempts to develop small molecule antagonists resulted in molecules with suboptimal pharmacokinetic profiles, low activity under physiological conditions, non-selectivity for C5aR, immunogenicity, and CYP enzyme liabilities [4,5,18].

We initiated a discovery and development effort to identify and characterize an orally administered, potent and selective C5aR antagonist. Our early characterization of CCX168 found that it was highly selective in binding to the human and primate C5a receptor. With either cultured human myeloid cells or with freshly-isolated neutrophils, CCX168 displayed sub-nanomolar antagonist activity under normal *in vitro* conditions in cell culture buffer, and no evidence of agonist activity by CCX168 in any of the assays used. CCX168 was highly potent in C5a-binding, chemotaxis, and calcium flux assays in buffer using U937 cells, and retained this high potency in the chemotaxis assay using neutrophils of human C5aR knock-in mice, as well as monkey neutrophils. These results were repeated in freshly isolated human neutrophils and CCX168 was shown to also block C5a-mediated CD11b upregulation in these cells. Importantly, CCX168 remained a potent inhibitor of C5aR in 100% human blood.

Since CCX168 does not bind to native mouse C5aR, we created a transgenic human C5aR knock-in mouse, and showed that the transgenic receptor functions as well as in humans based on a series of C5a-mediated CD11b upregulation experiments, and also in a C5a-induced neutrophil endothelial margination assay. Using these novel mice, we found that CCX168 was highly effective in ameliorating the glomerulonephritis induced by anti-myeloperoxidase antibodies [19]. Mice treated with 30 mg/kg CCX168 once daily had a 93% and 100% improvement, respectively, in the number of glomeruli with crescents or necrosis, and an improvement in proteinuria, hematuria, and leukocyturia.

A further demonstration of the role of alternative complement activation is that patients with active AAV were shown to have higher plasma levels of C3a, C5a, soluble C5b-9, and Bb compared to patients with AAV in remission, with the Bb levels correlating significantly with total and cellular crescents in renal biopsies [28]. A literature-based review also indicated that C3a and C5a, among other markers in blood, and MCP-1 and C5a in urine samples appeared to be promising markers in patients with AAV [29].

The cynomolgus monkey C5aR is highly homologous to the human receptor [30], and we showed that CCX168 was a potent inhibitor of C5a-induced neutrophil migration, and C5a-induced neutrophil margination in monkeys.

CCX168 was well tolerated and safe in a Phase 1 clinical trial in 48 healthy human volunteers at all doses tested (1 to 100 mg). The pharmacokinetic profile of CCX168 showed a dose-dependent increase in plasma CCX168 with single doses up to 100 mg. CCX168 blocked the C5a-mediated CD11b upregulation in freshly isolated neutrophils from the dosed subjects in a concentration-dependent manner. A dose regimen of 30 mg CCX168 twice daily provided C5aR coverage that was approximately 94% or higher throughout the day, based on assays done at the 2-hour and 12-hour time points after dosing on day 7 (steady state).

Based on an integrated analysis across multiple *in vitro*, preclinical, as well as this clinical trial, a CCX168 dose regimen of 30 mg twice daily was considered reasonable for clinical trials in patients with ANCA-associated vasculitis, which are currently being conducted.

In conclusion, CCX168 is a potent selective inhibitor of the human C5a receptor, based on its ability to block C5a-mediated chemotaxis, neutrophil margination, and CD11b upregulation in a variety of *in vitro* and preclinical models, and most recently in a Phase 1 clinical trial in healthy volunteers. Based on these data, we believe that CCX168 is a promising drug candidate for addressing the tissue and organ damage caused by excessive complement activation in ANCA vasculitis and diseases such as atypical hemolytic uremic syndrome.

## Supporting Information

S1 CONSORT Checklist CONSORT checklist for Phase 1 study. (DOC)

S1 Protocol Protocol for Phase 1 study. (PDF)

S1 Table Selectivity profile towards other chemokine receptors, cytochrome P450 enzymes and hERG patch clamp test. (DOCX)

S2 Table CCX168 selectivity profile against a broad panel of targets. (DOCX)
