# Correction: Characterization of Pharmacologic and Pharmacokinetic Properties of CCX168, a Potent and Selective Orally Administered Complement 5a Receptor Inhibitor, Based on Preclinical Evaluation and Randomized Phase 1 Clinical Study

**DOI:** 10.1371/journal.pone.0210593

**Published:** 2019-01-04

**Authors:** Pirow Bekker, Daniel Dairaghi, Lisa Seitz, Manmohan Leleti, Yu Wang, Linda Ertl, Trageen Baumgart, Sarah Shugarts, Lisa Lohr, Ton Dang, Shichang Miao, Yibin Zeng, Pingchen Fan, Penglie Zhang, Daniel Johnson, Jay Powers, Juan Jaen, Israel Charo, Thomas J. Schall

The x-axis labels are incorrect within Fig 4D, Fig 5A, Fig 5B, Fig 8A, and Fig 8B. The correct x-axis label is: C5a (M). Please see the correct Fig 4, Fig 5, and Fig 8 here.

**Fig 4:**
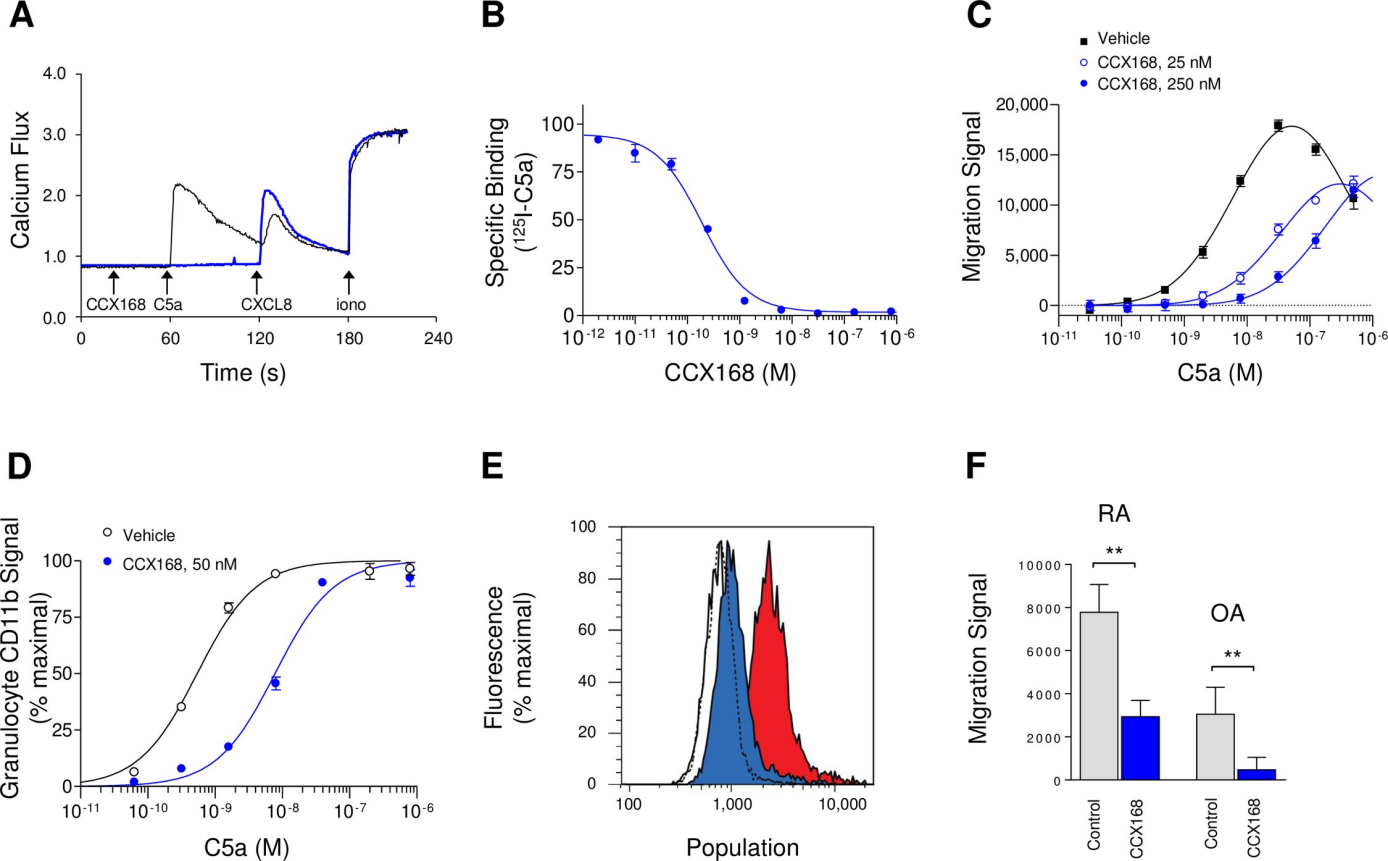
*In vitro* Characterization of CCX168 Using Freshly-Isolated Human Neutrophils or Human Whole Blood. **(**A) Sequential intracellular calcium release in human neutrophils in response to C5a (100 nM), CXCL8 (100 nM) or ionomycin (1 μg/mL), in the presence (blue line) or absence (black line) of CCX168 (10 μM). CCX168 blocked calcium release induced by C5a but not by the CXCR1 ligand CXCL8. (B) Binding of [^125^I]-C5a to human neutrophils in the presence of a range of concentrations of CCX168. CCX168 inhibited [^125^I]-C5a binding with a potency (IC_50_ value) of 0.2 nM. Each data point represents the mean of 4 replicates ± standard error, and the experiment was repeated 2 separate times. (C) C5a-induced chemotaxis of leukocytes in human whole blood, in the presence of vehicle control (■), and 25 nM (○) or 250 nM (●) CCX168. CCX168 inhibited leukocyte chemotaxis in a dose-dependent manner. Each data point represents the mean of 8 replicates ± standard error, and the experiment was repeated 5 separate times. (D) C5a-induced upregulation of CD11b on the surface of neutrophils in human whole blood in the presence of vehicle control (○) or 50 nM CCX168 (●). CCX168 inhibited CD11b upregulation. Each data point represents the mean of 4 replicates ± standard error, and the experiment was repeated 4 separate times. (E) C5a-induced oxidative burst in isolated human neutrophils in the presence of vehicle control (red histogram) or CCX168 (100 nM, blue histogram). The empty histograms represent untreated neutrophils (i.e., no C5a) in the presence of vehicle control (solid black line) or CCX168 (dotted black line). CCX168 blocked the C5a-induced oxidative burst but did not affect untreated neutrophils. The experiment was repeated 3 times. (F) Chemotaxis of leukocytes in human whole blood towards synovial fluid in the presence of vehicle control or CCX168 (100 nM). Experiments using synovial fluid from a patient with rheumatoid arthritis (RA) or osteoarthritis (OA) are shown. CCX168 inhibited leukocyte chemotaxis induced by each of these samples. Each bar represents the mean of 8 replicates ± standard error, and the experiment was repeated two times. **p<0.01 based on Student’s t-test.

**Fig 5:**
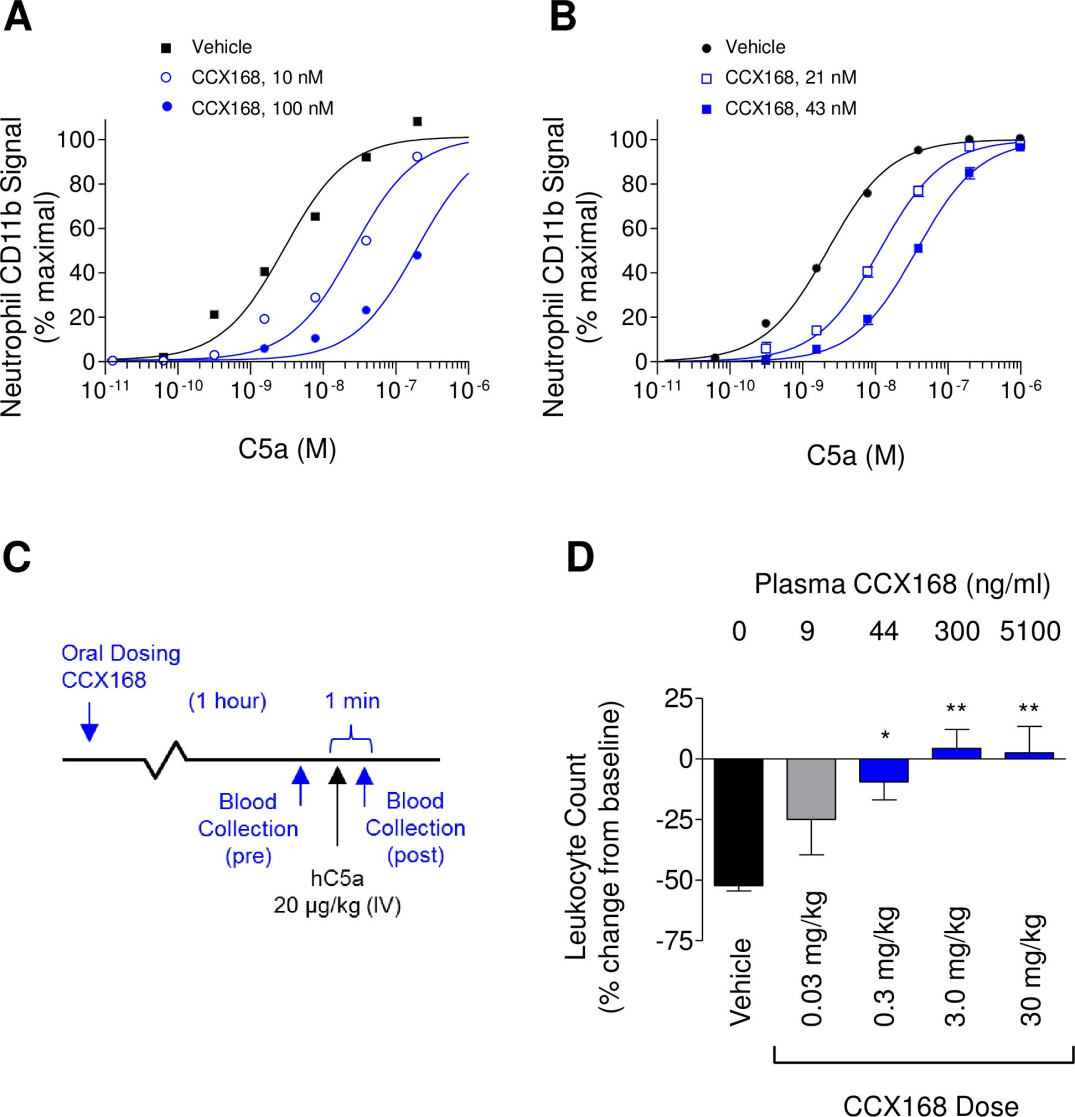
Biological Effects of CCX168 on Transgenic Human C5aR Knock-in Mice. (A) C5a-induced upregulation of CD11b on the surface of neutrophils in whole blood from human C5aR knock-in mice, after addition of vehicle (■), 10 nM (○), or 100 nM (●) CCX168. CCX168 inhibited CD11b upregulation in a dose-dependent manner. Each data point represents the mean of 3 replicates ± standard error; the study was repeated 3 separate times. (B) C5a-induced upregulation of CD11b on the surface of neutrophils in whole blood from human C5aR knock-in mice 1 hour after oral dosing with vehicle (●), 0.075 mg/kg (□), or 0.15 mg/kg (■) CCX168. Neutrophil CD11b upregulation was diminished in blood from CCX168-treated mice. Plasma concentrations of CCX168 are indicated. (C) Schematic of the C5a-induced leukopenia study in human C5aR knock-in mice. One hour following oral administration of CCX168, C5a (20 μg/kg) was administered intravenously, with blood drawn immediately before and 1 minute after C5a injection. Blood samples were analyzed for leukocyte numbers. (D) Effect of CCX168 in the C5a-induced leukopenia study in hC5aR knock-in mice. The percent change in the number of leukocytes in the blood sample collected after C5a injection, relative to the sample collected prior to C5a injection, is shown for each group (4 mice per group). Above each bar is the average concentration of CCX168 in the pre-injection blood samples. CCX168 inhibited the depletion of blood leukocytes caused by intravenous administration of C5a. *p<0.05, **p<0.01 based on Student’s t-test.

**Fig 8:**
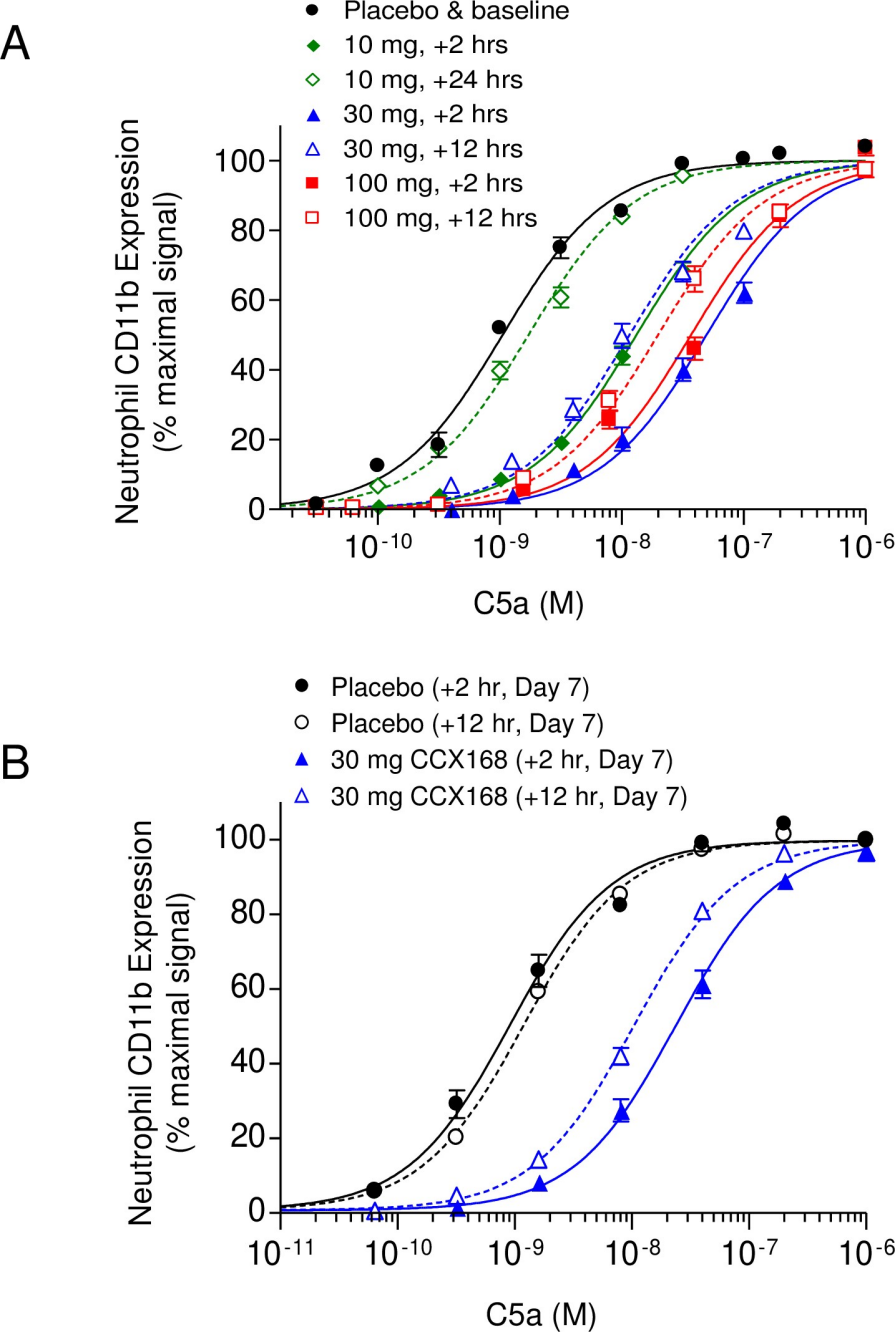
Pharmacodynamic Profile of CCX168 in Healthy Human Volunteers. Pharmacologic inhibition of C5aR by CCX168 as measured with an on-site, whole-blood *ex vivo* assay of C5a activity. (A) C5a-induced upregulation of CD11b on the surface of neutrophils in whole blood collected in the placebo group (●), and at 2 hours (♦) and 24 hours (◊) after a single dose of 10 mg CCX168, at 2 hours (▲) and 12 hours (Δ) after a single dose of 30 mg CCX168, and 2 hours (■) and 12 hours (□) after a single dose of 100 mg CCX168. CD11b upregulation was diminished in blood from subjects dosed with CCX168. (B) C5a-induced upregulation of CD11b on the surface of neutrophils in whole blood collected at 2 hours (●) or 12 hours (○) after the last dose of a 7-day regimen of placebo, or 2 hours (▲) or 12 hours (Δ) after the last dose of a 7-day regimen of 30 mg CCX168 given twice daily. CD11b upregulation was diminished in both blood samples from CCX168 subjects with a 10-fold decrease in C5a potency exhibited in the 12-hour samples.
