# Supplementary material for: Characterization of Pharmacologic and Pharmacokinetic Properties of CCX168, a Potent and Selective Orally Administered Complement 5a Receptor Inhibitor, Based on Preclinical Evaluation and Randomized Phase 1 Clinical Study
Source: PLoS One. 2016 Oct 21;11(10):e0164646. doi: 10.1371/journal.pone.0164646 (PMC5074546; doi:10.1371/journal.pone.0164646)
Supplement: S1 Protocol — (PDF) [file pone.0164646.s002.pdf]

PROTOCOL

TITLE PAGE

Study Title: A Double-Blind, Placebo-Controlled, Single and Multiple Ascending Dose Phase 1 Study to Evaluate the Safety, Tolerability, and Pharmacokinetics of CCX168 in Healthy Male and Female Subjects

Protocol Number: CL001_168

Investigational Product: CCX168

Indications: Vasculitis, Systemic Lupus Erythematosus (SLE)

Sponsor: ChemoCentryx, Inc.

Development Phase: 1

Sponsor's Responsible Medical Officer: Pirow Bekker, MD, PhD

ChemoCentryx, Inc

Mountain View, California, USA

Sponsor Signatory: Pirow Bekker, MD, PhD

Approval Date: 5 October 2009 FINAL

Confidential

The information contained herein is the property of the Sponsor and may not be reproduced, published, or disclosed to others without written authorization of the Sponsor.

This study will be conducted according to the principles of Good Clinical Practice as described in International Conference on Harmonization guidelines, including the archiving of essential documents.

INVESTIGATOR SIGNATORY PAGE

Protocol Number: CL001_168

Protocol Title: A Double-Blind, Placebo-Controlled, Single and Multiple Ascending Dose Phase 1 Study to Evaluate the Safety, Tolerability, and Pharmacokinetics of CCX168 in Healthy Male and Female Subjects

I agree:

- to assume responsibility for the proper conduct of the study at this site.
- to conduct the study in compliance with this protocol, any future amendments, and with any other study conduct procedures provided by ChemoCentryx, Inc.
- not to implement any deviations from or changes to the protocol without agreement from the sponsor and prior review and written approval from the Institutional Review Board (IRB)/Ethics Committee (EC), except where necessary to eliminate an immediate hazard to the subjects, or for administrative aspects of the study (where permitted by all applicable regulatory requirements).
- that I am thoroughly familiar with the appropriate use of the investigational drug(s), as described in this protocol, and any other information provided by the sponsor including, but not limited to the following: the current version of the Clinical Investigator's Brochure prepared by ChemoCentryx, Inc. and approved product label, if applicable.
- that I am aware of and will comply with current ICH/FDA good clinical practices guidelines (GCP) and all regulatory requirements.
- to ensure that all persons assisting me with the study are adequately informed about the investigational drug(s) and their study-related duties and function as described in the protocol.

[signature] 6-Oct-2009

Principal Investigator Date

Michael Seiberling, M.D.

Printed Name

Address* COVANCE Clinical Research Unit AG
Lettenweg 118
CH-4123 Allschwil (Basel)
Switzerland

Phone Number* +41 61 487 2400

* If the address or phone number needs to be changed during the course of the study, this will be done by the Investigator, with written notification to the Sponsor, and will not require (a) protocol amendment(s).

SPONSOR CONTACT INFORMATION

Protocol Number: CL001_168

Protocol Title: A Double-Blind, Placebo-Controlled, Single and Multiple Ascending Dose Phase 1 Study to Evaluate the Safety, Tolerability, and Pharmacokinetics of CCX168 in Healthy Male and Female Subjects

Medical Monitor	Pirow Bekker, MD, PhD<br>Senior Vice President, Medical and Clinical Affairs<br>ChemoCentryx, Inc<br>Mountain View, California USA<br>Office telephone number: 650-210-2924<br>Fax number: 650-210-2910<br>Email: pbekker@chemocentryx.com
Clinical Operations Manager	Dan Johnson, MA<br>Clinical Project Manager<br>ChemoCentryx, Inc<br>Mountain View, California USA<br>Office telephone number: 650-210-2923<br>Fax number: 650-210-2910<br>Email: djohnson@chemocentryx.com

SPONSOR SIGNATURE FOR APPROVAL

Protocol Number: CL001_168

Protocol Title: A Double-Blind, Placebo-Controlled, Single and Multiple Ascending Dose Phase 1 Study to Evaluate the Safety, Tolerability, and Pharmacokinetics of CCX168 in Healthy Male and Female Subjects

[signature] 5-OCT-2009

Pirow Bekker, MD, PhD Date

Senior Vice President, Medical and Clinical Affairs

TABLE OF CONTENTS

TITLE PAGE 1
INVESTIGATOR SIGNATORY PAGE 2
SPONSOR CONTACT INFORMATION 3
SPONSOR SIGNATURE FOR APPROVAL 4
TABLE OF CONTENTS 5
SYNOPSIS 7
STUDY SCHEMA 15
TIME AND EVENTS TABLE FOR PERIOD 1 16
TIME AND EVENTS TABLE FOR PERIOD 2 17
LIST OF ABBREVIATIONS 18
1 Introduction 20
1.1 Background 20
1.2 Study Drug Development 21
1.2.1 Non-Clinical Pharmacology 21
1.2.2 Non-Clinical Safety and Toxicology 22
1.2.3 Non-Clinical ADME 25
1.3 Prior Human Experience 26
1.4 Rationale for the Study 26
2 Objectives 26
2.1 Primary Objective 26
2.2 Secondary Objectives 26
3 Study Design 27
4 Study Population 28
4.1 Size of the Population 28
4.2 Inclusion Criteria 28
4.3 Exclusion Criteria 29
4.4 Removal of Subjects from Therapy of Assessment 30
5 Study Medication/Treatment 30
5.1 Product Characteristics 30
5.2 Randomization and Method of Treatment Assignment 30
5.3 Doses and Regimens 30
5.4 Rationale for Dose Selection 32
5.5 Drug Supply 33
5.5.1 Packaging and Labeling 33
5.5.2 Storage 33
5.6 Blinding 33
5.7 Drug Accountability 33
5.8 Treatment Compliance 34
5.9 Concomitant Medications and Restrictions 34
6 Study PROCEDURES 34
6.1 Screening and Enrollment 34
6.2 Study Conduct 35
7 Study Assessments 35
7.1 Efficacy Assessments 35
7.2 Safety Assessments 35
7.2.1 Physical Examinations and Vital Signs 35
7.2.2 Clinical Laboratory Assessments 36

7.2.3 Reporting of Adverse Events....37
7.2.4 Reporting of Serious Adverse Events....38
7.3 Pharmacokinetic Assessments....38
7.4 Pharmacodynamic Assessments....39
7.4.1 CD11b Assays....39
7.4.2 Whole Blood Migration Assays....39
7.5 Study Completion and Withdrawal....40
7.6 Data Monitoring Committee....40
7.7 Statistical Methods....40
7.8 Subject Populations....41
7.8.1 Intent-to-Treat....41
7.8.2 Safety....41
7.8.3 Evaluable....41
7.9 Efficacy Endpoints....41
7.10 Safety Endpoints....41
7.11 Pharmacokinetic Endpoints....41
7.12 Pharmacodynamic Endpoints....42
7.13 Statistical Analysis Methodology....42
7.13.1 Baseline Characteristics....42
7.13.2 Primary Efficacy Analysis....42
7.13.3 Secondary Efficacy Analyses....42
7.13.4 Covariates....42
7.13.5 Safety Analyses....42
7.13.6 Pharmacokinetic Analysis....43
7.13.7 Pharmacodynamic Analysis....45
7.14 Sample Size Justification....45
7.15 Protocol Deviations....45
8 Study Completion and Termination....45
8.1 Study Completion....45
8.2 Study Termination....45
9 Regulatory and Administrative Requirements....45
9.1 Investigator Responsibilities....45
9.2 Institutional Review Board or Ethics Committee....46
9.3 Informed Consent....46
9.4 Protocol Modifications....46
9.5 Regulatory Documentation....46
9.6 Subject Identification Register....46
9.7 Record Retention....47
9.8 Case Report Form Completion....47
9.9 Monitoring....47
9.10 Data Quality Assurance....47
9.11 On-site Audits....47
9.12 Use of Information and Publication....48
10 References....48
11 Appendices....49
11.1 Statement of Obligations of Sponsor, Monitor, and Clinical Investigator....49
11.2 Informed Consent Form....52
11.3 Common Terminology Criteria for Adverse Events v3.0 (CTCAE)....53

SYNOPSIS

Name of Sponsor	Name of Active Ingredient	Study number:
ChemoCentryx, Inc	CCX168	CL001_168

Title

A Double-Blind, Placebo-Controlled, Single and Multiple Ascending Dose Phase 1 Study to Evaluate the Safety, Tolerability, and Pharmacokinetics of CCX168 in Healthy Male and Female Subjects

Investigator

Michael Seiberling, M.D.

Study center

Covance

Study period	Phase of development
4 months	Phase 1

Objectives

The primary objective of this study is to evaluate the safety and tolerability of single and multiple oral doses of CCX168, over a range of dose levels, in healthy male and female subjects. The secondary objectives of this study are to evaluate the:

- Single and multiple dose pharmacokinetic profile of CCX168 over a range of dose levels,
- Relationship between CCX168 plasma concentrations and C5aR-dependent CD11b upregulation in circulating neutrophils, and
- Relationship between CCX168 plasma concentrations and C5aR-dependent cell migration in a whole blood migration assay.

Methodology

Up to 40 healthy male or female subjects will complete this double-blind, placebo-controlled, dose escalation study. This is the first time that CCX168 will be administered to humans. There will be two study periods. During Period 1, subjects will be enrolled in up to five serial cohorts of eight subjects each. CCX168 doses of 1, 3, 10, 30, and 100 mg will be tested. Dose escalation will continue as planned unless safety or tolerability issues appear that prevent further dose escalation. During the second study period, subjects will receive the same dose as during the first period but once daily for a period of 7 days continuously. The lowest dose cohort in Period 2 will only be dosed after the same dose and two higher doses have been given in the single-dose study period (Period 1).

The first dose cohort in Period 1 will be dosed in a staggered way so that 2 of the 8 subjects will be dosed on the first day (one randomized to placebo and one randomized to CCX168), the third and fourth subjects will be dosed at least 48 hours after the first 2 subjects have been

dosed (one randomized to placebo and one randomized to CCX168), and the rest of the subjects (4 of 8) will be randomized 3:1, CCX168:placebo and dosed at least 48 hours after the first 4 subjects have been dosed. Subjects in the other dose cohorts will be dosed on the same day for each cohort, but with intervals of at least 10 minutes between dosing of subjects in the cohort. In Cohorts 2 to 5, subjects will be randomized 6:2, CCX168:placebo.

Dose escalation in Period 1 will occur only after review by the Investigator and the Sponsor's Medical Monitor of all available pharmacokinetic data, and of safety data for at least three days after the initial dosing from all subjects in each of the preceding cohort(s). The National Cancer Institute (NCI) Common Terminology Criteria (CTC) for Adverse Events v3.0 (Appendix 11.3) will be used to assess dose-limiting toxicities. Occurrence of the same Grade 1 or higher CTC scale toxicities in two or more subjects in any cohort will be considered possible evidence of dose-limiting toxicity. If the adverse events are considered possibly related to study drug, the following options will be considered: cease dosing, administer a dose lower than that received by the prior cohort, or repeat a previous dose level. Occurrence of a Grade 2 or higher toxicity in one subject in any cohort will be considered evidence of possible dose-limiting toxicity. If the adverse events are considered possibly related to study drug, the following options will be considered: cease dosing, administer a dose lower than that received by the prior cohort, or repeat a previous dose level. In the absence of dose-limiting adverse events, dose escalation will continue up to a maximum dose of 100 mg (single dose). Clinical judgment must be exercised for any clinically significant toxicity, which may trigger the actions described above (cease dosing, administer a dose lower than that received by the prior cohort, or repeat a previous dose level).

Dose escalation in Period 2 will only occur after the next two higher single doses have been tested and found to be safe, for example, 1 mg CCX168 will only be tested in the multi-dose period after 1, 3, and 10 mg CCX168 dose has been tested and found to be safe in the single-dose Period 1. Furthermore, dose escalation in Period 2 will only occur if the preceding lower dose given daily for 7 days has been found safe. The same CTC criteria and stopping rules of Period 1 apply to the Period 2 dosing.

It is planned that the subjects who complete Period 1 also participate in Period 2 to determine the safety and tolerability, pharmacokinetic (PK) and pharmacodynamic (PD) profile of CCX168 when given daily for 7 days. If a subject who participated during Period 1 decided not to participate in Period 2, the subject will be replaced for Period 2. Treatment allocation for the subjects participating in the Period 2 evaluation (i.e., subject assignment to active or placebo treatment) and dose will be identical to the Period 1 assignment, e.g., subjects assigned to receive a 1 mg single dose of CCX168 during Period 1 will receive 1 mg CCX168 daily for 7 days during Period 2. Replacement subjects will receive the same treatment as the subjects they replace.

In Period 1, subjects will remain at the study center for at least 24 hours after administration of study medication, during which time they will be observed closely for safety and blood samples will be collected for PK and PD analysis. Subjects will return to the study center on Study Days 3, 4, and 8 for collection of blood samples and for safety monitoring. Visits for Study Days 2, 3, 4, and 8 must occur on the scheduled days. All subjects will return to the study center for a second treatment period following a wash-out period of at least fourteen days from the Period 1 dosing. For Period 2, subjects will remain at the study center for the duration of the 7-day dosing period. During this time, subjects will be observed closely for safety and blood samples will be collected for PK and PD analysis. Subjects will be discharged from the study center on Day 8, and will return to the study center on Days 9, 10, and 15 for collection of blood samples

and for safety monitoring. Study Days 9, 10, and 15 must occur on the scheduled days. Subjects will be terminated from the study at the completion of the telephone contact on Study Day 29 of their second treatment period. The telephone contact on Study Day 29 could occur within +/- 2 days of the scheduled day.

To the extent possible, any adverse events that are deemed study drug-related and are ongoing at discharge will be followed-up to resolution or until a determination is made that the unresolved event is stable.

Number of Subjects

Healthy male or female subjects will be entered sequentially into one of up to five cohorts of eight subjects each. No more than five subjects within a cohort may be of the same gender. Up to 40 subjects will complete the study. Subjects who terminate prematurely before completing the sample collections through Study Day 3 of Period 1 may be replaced as warranted to ensure that at least six evaluable subjects from each dose group complete Period 1. Replacements for subjects who terminate prematurely will complete both treatment periods. Subjects who discontinue after Period 1 but before Period 2 will be replaced. Subjects who discontinue during Period 2 will not be replaced.

Main Criteria for Inclusion

1. Male or female subjects, aged 19-45 years inclusive, who are in generally good health, whose body mass index is 19 to 29 kg/m^2;
2. Willing and able to give written Informed Consent and to comply with the requirements of the study protocol;
3. Negative result of the human immunodeficiency virus (HIV) screen, the hepatitis B screen, and the hepatitis C screen;
4. Judged to be healthy by the Investigator, based on medical history, physical examination (including electrocardiogram [ECG]), and clinical laboratory assessments. Subjects with clinical laboratory values that are outside of normal limits and/or with other abnormal clinical findings that are judged by the Investigator not to be of clinical significance may be entered into the study;
5. Female subjects of childbearing potential, and male subjects with partners of childbearing potential, may participate if adequate contraception is used during, and for at least the four weeks after, any administration of study medication. Adequate contraception is defined as usage by at least one of the partners of a barrier method of contraception, together with usage by the female partner, commencing at least three months prior to Screening, of a stable regimen of any form of hormonal contraception or an intra-uterine device. Use of abstinence alone is not considered adequate. Use of a barrier method alone is considered adequate only if the male partner was vasectomized at least six months prior to Screening. Use of a double-barrier method of contraception is acceptable. Women who are not of childbearing potential may participate if they are surgically sterile or if they are at least two years post menopause.

Main Criteria for Exclusion

1. Women who are pregnant, breastfeeding, or have a positive serum pregnancy test at

Screening and/or on Study Day -1;

2. Expected requirement for use of any medication (with the exception of continuing use by female subjects of hormonal contraceptives in accordance with a regimen that has been stable for at least the three months prior to Screening) during the study period;
3. History within the three months prior to study entry of use of tobacco and/or nicotine-containing products;
4. History within one year prior to study entry of illicit drug use;
5. History of alcohol abuse at any time in the past;
6. History of any form of cancer;
7. Consumed alcoholic beverages, or any food or drink containing grapefruit or grapefruit juice within 24 hours of screening;
8. History or presence of any medical condition or disease which, in the opinion of the Investigator, may place the subject at unacceptable risk for study participation;
9. Donated or lost more than 350 mL of blood or blood products within 56 days prior to screening, or donated plasma within 7 days of randomization;
10. Subject's hemoglobin less than 12 g/dL (or less than 7.45 mmol/L);
11. Participated in any clinical study of an investigational product within 30 days prior to randomization;
12. Subject has any evidence of hepatic disease; AST, ALT, GGT, alkaline phosphatase, or bilirubin > 1.5 x the upper limit of normal;
13. Subject has any evidence of renal impairment; serum creatinine > 1.5 x upper limit of normal;
14. Subject's urine tested positive at Screening and/or on Study Day -1 for any of the following: opioids, amphetamines, cannabinoids, benzodiazepines, barbiturates, cocaine, cotinine, or alcohol (Breathalyzer test allowed for alcohol).

Test Product

CCX168 will be administered as a solution to all dose cohorts in both treatment periods. Subjects will drink 30 mL of water following the dose solution. Two formulations will be tested in Period 1. Based on the PK profiles of the two formulations, one will be selected for Period 2. CCX168 will be administered in accordance with the following plan:

- 1 mg CCX168 – Cohort 1
- 3 mg CCX168 – Cohort 2
- 10 mg CCX168 – Cohort 3
- 30 mg CCX168 – Cohort 4

- 100 mg CCX168 – Cohort 5

Within each cohort of eight subjects, six subjects will be assigned to receive CCX168, except for Cohort 1 (five to CCX168 and three to placebo). All doses of CCX168 will be administered orally, on a double-blind basis. Subjects will fast (with the exception of water) beginning at least ten hours prior to administration of study medication and continuing until at least four hours after administration of study medication for both treatment periods. Water will be allowed as desired, except for the 1-hour periods before and immediately after study drug administration.

Reference Therapy, Dose and Mode of Administration

Placebo will consist of the dosing solutions minus CCX168 for all cohorts. Within each cohort of eight subjects, two subjects will be assigned to receive placebo (except for Cohort 1 in which three subjects will receive placebo). In order to protect the study blind, these subjects will receive matching volumes of the same dosing solutions. Subjects will drink 30 mL of water following the dose solution. Subjects will fast (with the exception of water) beginning at least ten hours prior to administration of study medication and continuing until at least four hours after administration of study medication for both treatment periods. Water will be allowed as desired, except for the 1-hour periods before and immediately after study drug administration.

Duration of Treatment and Observation

Subjects will be screened within 21 days prior to study entry. Subjects will be admitted to the study center on Study Day -1 of Period 1. On Study Day 1 of Period 1, subjects will receive a single dose of study medication. Subjects will remain at the study center for at least 24 hours after administration of study medication, during which time they will be observed closely for safety and blood samples will be collected for PK and PD analysis. Subjects will return to the study center on Study Days 3, 4, and 8 for collection of blood samples and for safety monitoring. All subjects will return to the study center for a second treatment period following a wash-out period of at least fourteen days from first dosing. For Period 2, subjects will remain at the study center for the duration of the 7-day dosing period. During this time, subjects will be observed closely for safety and blood samples will be collected for PK and PD analysis. Subjects will be discharged from the study center on Day 8, and will return to the study center on Days 9, 10, and 15 for collection of blood samples and for safety monitoring. Subjects will be terminated from the study at the completion of the telephone contact on Study Day 29 of their second treatment period.

To the extent possible, any adverse events that are deemed study drug-related and are ongoing at discharge will be followed-up to resolution or until a determination is made that the unresolved event is stable.

Safety Assessments

Safety will be evaluated by periodic physical examinations and body system reviews, assessments of vital signs, routine clinical laboratory tests (including blood chemistry, hematology, and urinalysis), electrocardiographic (ECG) monitoring, and monitoring of adverse events.

Pharmacokinetic Assessments

Concentrations of CCX168 will be determined in plasma from 6-mL blood samples collected in K_2EDTA tubes at the following times during Period 1.

- Study Day 1: immediately prior to administration of study medication (Time 0), and at Hours 0.08, 0.25, 0.5, 1, 1.5, 2, 3, 4, 6, 10, and 12 after administration of study medication
- Study Day 2: at Hours 18, and 24 after administration of study medication
- Study Day 3: at approximately Hour 48 after administration of study medication
- Study Day 4: at approximately Hour 72 after administration of study medication
- Study Day 8: at approximately Hour 168 after administration of study medication

Concentrations of CCX168 will be determined in plasma from 6-mL blood samples collected in K_2EDTA tubes at the following times during Period 2.

- Study Day 1: immediately prior to administration of study medication (Time 0), and at Hours 0.08, 0.25, 0.5, 1, 1.5, 2, 3, 4, 6, 10, and 12 after administration of study medication
- Study Day 2: at Hours 18, and 24 after first administration of study medication
- Study Day 3: at approximately Hour 48 after first administration of study medication
- Study Day 4: at approximately Hour 72 after first administration of study medication
- Study Day 5: at approximately Hour 96 after first administration of study medication
- Study Day 6: at approximately Hour 120 after first administration of study medication
- Study Day 7: immediately prior to 7th administration of study medication (Time 144), and at Hours 144.08, 144.25, 144.5, 145, 145.5, 146, 147, 148, 150, 154, and 156 after first administration of study medication
- Study Day 8: at Hours 162, and 168 after first administration of study medication
- Study Day 9: at approximately Hour 192 after first administration of study medication
- Study Day 10: at approximately Hour 216 after first administration of study medication
- Study Day 15: at approximately Hour 336 after first administration of study medication

Pharmacodynamic Assessments

Blood samples will be collected from subjects in the 10 mg, 30 mg, and 100 mg dose cohorts to determine the relationship between CCX168 plasma concentrations and inhibition of C5aR-dependent upregulation of CD11b, as well as migration of peripheral blood neutrophils. In Period 1, the samples will be collected at pre-dose and Hours 2 and 24 after dosing.

Depending on the results obtained from Period 1 samples, the pharmacodynamic samples might not be collected in Period 2. If required, the Period 2 samples will be drawn before the first dose of this period and Hour 146 (2 hours after the 7th daily dose), and Hour 168 (24 hours after the 7th daily dose). The time of the Hour 2 collection in Period 1 and Hour 146 in Period 2 may be adjusted for the 30 and 100 mg dose cohorts based on the actual T_{max} of CCX168 for the 10 mg CCX168 dose.

Statistical Methods

Screening, compliance, safety and tolerability data

All baseline subject characteristics of demographic data (age, height, weight, race), smoking status, medical history (abnormalities only), physical examination (abnormalities only), ECG, and concomitant medications at study entry will be listed. Demographics will be summarized. All clinical safety and tolerability data will be listed for each subject and by treatment. Data from all placebo subjects will be pooled. Individual vital signs and individual differences from baseline in vital signs will be listed by treatment and measurement time and summarized descriptively. ECG assessments will be listed. Laboratory values outside the laboratory's normal ranges will be listed separately, together with associated repeats and comments as to their clinical significance. All reported adverse events will be coded using MedDRA and listed. Treatment-emergent adverse events will be summarized by treatment and by other categorical information of interest.

Pharmacokinetic analysis

Individual plasma concentrations of CCX168 will be listed, plotted, and summarized descriptively and graphically for each treatment. The following parameters will be determined for Period 1 and for Days 1 and 7 of Period 2, where applicable:

C_{max}	Maximum plasma concentration
t_{max}	Time of maximum plasma concentration
λ_z	Terminal phase rate constant
$t_{½z}$	Apparent terminal half-life
CL/F	Apparent oral clearance
V_z/F	Apparent volume of distribution
CL_{ss}/F	Apparent oral clearance at steady-state
V_{zss}/F	Apparent volume of distribution at steady-state
AUC_{0-t}	Area under the plasma concentration-time curve from Time 0 to Time t (time of last quantifiable plasma concentration)
AUC_{inf}	Area under the plasma concentration-time curve from Time 0 to infinity
AUC_{0-24}	Area under the plasma concentration-time curve from Time 0 to 24h

In addition, the accumulation ratio (AUC_{0-24} for Day 7 divided by Day 1) will also be calculated.

Dose-proportionality will be assessed using the "power model" approach based on the 90% confidence interval for the slope.

Pharmacodynamic analysis

The degree of C5aR occupancy by CCX168 for each subject at each time point will be determined based on the results from the CD11b-upregulation and migration experiments. Specifically, the endpoints will include either the percent inhibition of a C5a-induced signal in comparison to controls or else a measure of the dose ratio brought about by CCX168 on a C5a dose response in comparison to controls. This dose ratio will be given as an A_x value, where A_2 is the concentration of drug that gives rise to a two-fold shift in the agonist dose-response curve.

Results will be listed and summarized descriptively and graphically for each subject and treatment group.

STUDY SCHEMA

Study Period 1

Single Dose 1 mg*→Safety Review→3 mg→SR**→10 mg→ SR→30 mg→ SR→100 mg→ SR

Study Period 2

Multiple Dose (7 days) → 1 mg→SR→3 mg→SR→10 mg→ SR→30 mg→ SR→100 mg→ SR

* Staggered dosing (2, 2, 4 subjects)
** SR = Safety Review meeting

TIME AND EVENTS TABLE FOR PERIOD 1

	Screen	Treatment Day [7]					
**Study Day**	**-21 to -2**	**-1**	**1**	**2**	**3**	**4**	**8**
Informed Consent	X						
Demographics/Medical History	X						
Complete Physical Examination, Weight, Height[1]	X						X
Body System Review		X	X	X	X	X	
Digitally acquired 12-lead ECG[2]	X	X	X	X	X	X	X
Vital Signs[3]	X	X	X[8]	X	X	X	X
Blood Chemistry, Hematology, PT, aPTT[4]	X	X		X		X	X
Urinalysis[5]	X	X		X		X	X
HIV, HBV, HCV Screening	X						
Urine Screen for Drugs of Abuse, Cotinine, and Alcohol (Breathalyzer allowed)	X	X					
Pregnancy Testing[6]	X	X					
Randomization		X[9]					
Study Drug Administration			X[10]				
PK Sample Collection			X[11]	X[12]	X	X	X
PD Sample Collection			X[13]				
Concomitant Medications	X	X	X	X	X	X	X
Adverse Events		X	X	X	X	X	X
Overnight clinic stay		X	X				

1 Height measured at Screening only
2 Assessed at Screening, Day -1, at Time 0 (in duplicate), at Hours 0.5, 1, 2, 3, 4, 8, 12, and 24 (Day 2) after initial administration of study medication, and on Study Days 3, 4, and 8. 12-lead ECGs will be acquired and stored digitally for analysis.
3 Assessment of heart rate, oral body temperature, blood pressure (orthostatic at Screening and at all other time points for 24 hours after dosing), and respiration rate
4 PT, prothrombin time; aPTT, activated partial thromboplastin time
5 This also includes assessment of the color of the urine.
6 For all women. Pregnancy testing only needs to be repeated on Day -1 if there were more than 14 days since testing done at Screening.
7 All study visits through Day 8 must occur on the scheduled days
8 Assessed at Time 0 and at Hours 0.5, 1, 2, 3, 4, 8, 12, and 24 (Day 2) after administration of study medication.
9 Randomization on Day 1 prior to dosing is allowed.
10 Subjects will fast (with the exception of water) beginning at least 10 hours prior to administration of study medication and continuing until at least 4 hours after administration of study medication.
11 Blood samples (6 mL) collected at Time 0 and at Hours 0.08, 0.25, 0.5, 1, 1.5, 2, 3, 4, 6, 10, and 12 after administration of study medication.
12 Blood samples (6 mL) collected at Hours 18 and 24 after administration of study medication.
13 Blood samples (25 mL) collected at Pre-dose and at Hours 2 and 24 for ONLY the 10 mg, 30 mg, and 100 mg CCX168 dose cohorts.

TIME AND EVENTS TABLE FOR PERIOD 2

	Treatment Day [7]												
**Study Day**	**-1**	**1**	**2**	**3**	**4**	**5**	**6**	**7**	**8**	**9**	**10**	**15**	**29**
Complete Physical Examination, Weight	X		X						X			X	
Body System Review		X		X	X	X	X	X		X	X		
Digitally acquired 12-lead ECG[1]	X	X	X		X			X	X			X	
Vital Signs[2]	X	X	X	X	X	X	X	X	X	X	X	X	
Blood Chemistry, Hematology, PT, aPTT[3]	X		X		X				X			X	
Urinalysis[4]	X		X		X				X			X	
Urine Screen for Drugs of Abuse, Cotinine, and Alcohol (Breathalyzer allowed)	X												
Pregnancy Testing[5]	X												
Study Drug Administration[6]		X	X	X	X	X	X	X					
PK Sample Collection		X[8]	X[9]	X[9]	X[9]	X[9]	X[9]	X[10]	X[10]	X	X	X	
PD Sample Collection		X[11]						X[11]					
Concomitant Medications	X	X	X	X	X	X	X	X	X	X	X	X	X
Adverse Events	X	X	X	X	X	X	X	X	X	X	X	X	X
Overnight clinic stay	X	X	X	X	X	X	X	X					
Telephone contact													X

[1] Assessed at Day -1, at Time 0 (in duplicate), at Hours 0.5, 1, 2, 3, 4, 8, 12, and 24 (Day 2) after initial administration of study medication, on Study Day 4, on Study Day 7 at Hours 144, 144.5, 145, 146, 147, 148, 152, 156, and 168 (Day 8) and Day 15. 12-lead ECGs will be acquired and stored digitally for analysis.
[2] Assessment of heart rate, oral body temperature, blood pressure, and respiration rate (supine, after at least 3 minutes of rest)
[3] PT, prothrombin time; aPTT, activated partial thromboplastin time
[4] This also includes assessment of the color of the urine
[5] For all women
[6] Subjects will fast (with the exception of water) beginning at least 10 hours prior to administration of study medication and continuing until at least 4 hours after administration of study medication on each of the 7 dosing days.
[7] All study visits through Day 15 must occur on the scheduled days; the Day 29 telephone visit may occur within +/- 2 days of the scheduled visit.
[8] Blood samples (6 mL) collected at Time 0 and at Hours 0.08, 0.25, 0.5, 1, 1.5, 2, 3, 4, 6, 10, and 12 after administration of the first dose of study medication.
[9] Blood samples (6 mL) collected at Hours 18 and 24 (Day 2), Hour 48 (Day 3), Hour 72 (Day 4), Hour 96 (Day 5), and Hour 120 (Day 6) after administration of the first dose of study medication.
[10] Blood samples (6 mL) collected at Hours 144, 144.08, 144.25, 144.5, 145, 145.5, 146, 147, 148, 150, 154, and 156 (Day 7), and Hours 162 and 168 (Day 8) after administration of the first dose of study medication.
[11] Depending on the results obtained from Period 1 samples, the pharmacodynamic samples might not be collected in Period 2. If required, blood samples (25 mL) will be drawn before the first dose of Period 2 and at Hours 146 and 168 for ONLY the 10 mg, 30 mg, and 100 mg CCX168 dose cohorts.

LIST OF ABBREVIATIONS

AE	adverse event
ALT	alanine aminotransferase (also called SGPT)
ANCA	anti-neutrophil cytoplasmic antibodies
API	active pharmaceutical ingredient
AST	aspartate aminotransferase (also called SGOT)
APTT	activated partial thromboplastin time
BLQ	below limit of quantification
BUN	blood urea nitrogen
C3a	complement 3a
C4a	complement 4a
C5a	complement 5a
C5aR	complement 5a receptor
C5b9	complement 5b9
CPK	creatinine phosphokinase
CRA	Clinical Research Associate (also known as the Study Monitor)
CRF	case report form
CRO	contract research organization
EC	ethics committee
EC_{50}	50% effective concentration
ECG	electrocardiogram
FACS	fluorescence activated cell sorting
FDA	Food and Drug Administration
FLIPR	Fluorometric Imaging Plate Reader
g	gram
GCP	good clinical practice
GGT	Gamma-glutamyl transpeptidase
GPCR	G protein-coupled receptor
HED	human equivalent dose
HEENT	head, eyes, ears, nose, throat
HIV	human immunodeficiency virus
IC_{50}	concentration to inhibit 50%
ICH	International Conference on Harmonisation
INR	International Normalized Ratio
IRB	Institutional Review Board
K_2EDTA	ethylene diamine tetra-acetic acid
LDH	lactate dehydrogenase
kg	kilogram
MABEL	Minimal Anticipated Biological Effect Level
MAC	membrane attack complex
MCH	mean cell hemoglobin
MCHC	mean cell hemoglobin concentration
MedDRA	Medical Dictionary for Drug Regulatory Affairs
mg	milligram
mL	milliliter
N	number
NOAEL	No observed adverse effect level
PD	pharmacodynamic(s)
PK	pharmacokinetic(s)
PT	prothrombin time

RBC	red blood cell
SAE	serious adverse event
SGPT	serum glutamic pyruvic transaminase (also called ALT)
SGOT	serum glutamic oxaloacetic transaminase (also called as AST)
SLE	systemic lupus erythematosus
SOP	standard operating procedure
WBC	white blood cell

1 INTRODUCTION

1.1 Background

The activation of the complement pathway generates biologically active fragments of complement proteins, e.g. C3a, C4a and C5a anaphylatoxins and C5b-9 membrane attack complexes (MAC), all of which mediate inflammatory responses by inducing leukocyte chemotaxis, activating macrophages, neutrophils, platelets, mast cells and endothelial cells and by increasing vascular permeability, cytolysis and tissue injury.

C5a is one of the most potent pro-inflammatory mediators of the complement system, being at least 100 times more potent than C3a. This 190 kD polypeptide, along with a C5b fragment, is produced by enzymatic cleavage of a C5 precursor during activation of any of the 3 complement pathways. C5a induces expression of adhesion molecules and chemotactic migration of neutrophils, eosinophils, basophils and monocytes. It also mediates inflammatory reactions by causing smooth muscle contraction, increasing vascular permeability, inducing basophil and mast cell degranulation and inducing release of lysosomal proteases and oxidative free radicals. The anaphylactic and chemotactic effects of C5a are mediated through its interaction with the C5a receptor (C5aR), a G protein-coupled receptor (GPCR) expressed on human neutrophils, monocytes, basophils, eosinophils, renal glomerular tissues, and lung smooth muscle and endothelial cells.

Recently, several reports have shown that anti-neutrophil cytoplasmic antibody (ANCA)-induced glomerulonephritis in mice (a model that closely recapitulates the histological features of human pauci-immune necrotizing crescentic glomerulonephritis in Wegener's granulomatosis and microscopic polyangiitis) is dramatically ameliorated by genetic deletion of either C5 or C5aR (Schreiber et al., 2008). The development of Systemic Lupus Erythematosus (SLE) is associated with the deposition of IgG-containing immune complexes in various tissues/organs, with the ensuing activation of the complement cascade and production of inflammatory stimuli such as C5a. Glomerular expression of C5aR mRNA and protein was shown to correlate positively with the degree of mesangial hypercellularity and level of serum creatinine in mesangial glomerulonephritis, including lupus nephritis (Abe et al., 2001). Recent studies showed that C5aR-deficient mice and mice treated with a small peptidic anti-C5aR antagonist are protected from tissue injury induced by immune complex formation. In addition, use of a C5 mAb in a spontaneous mouse model of lupus-like autoimmune disease resulted in significant amelioration of the course of glomerulonephritis and in markedly increased survival. A genetic version of the disease (MRLlpr mice) is also attenuated significantly when the C5aR receptor is deleted from that genetic background.

The therapeutic indications considered initially for CCX168, a potent and selective C5aR antagonist, are in the treatment of vasculitis or SLE.

Wegener's granulomatosis currently is treated with glucocorticosteroids and cyclophosphamide. Current treatment options for SLE range from the use of NSAIDs and anti-malarials (e.g., hydroxychloroquine) for less severe cases, to the use of glucocorticosteroids and immunosuppressive agents such as cyclophosphamide for the most severe cases. Several biologic therapies have been tested without success. Recently, the BLyS antibody belibumab has shown evidence of efficacy in an SLE study. Even with this success, many patients are not effectively treated and there remains a significant unmet medical need. Current treatments have considerable side effects and risks. An orally-available small

molecule therapeutic that demonstrates efficacy and safety may benefit patients with vasculitis and SLE.

1.2 Study Drug Development

1.2.1 Non-Clinical Pharmacology

1.2.1.1 In-Vitro Efficacy and Selectivity for C5aR

CCX168 is a potent antagonist of the human C5a receptor (hC5aR). As measured *in vitro* with a myeloid human cell line, CCX168 functionally inhibits C5a-mediated chemotaxis with a potency (IC_{50}) of 0.92 nM. Additionally, CCX168 displaces ^{125}I-C5a from hC5aR with a potency (IC_{50}) of 0.65 nM. When tested on freshly isolated human neutrophils, CCX168 inhibits the C5a-mediated increase in cytoplasmic calcium levels with a potency (IC_{50}) of 0.2 nM.

CCX168 has been evaluated for its ability to inhibit the C5a-mediated chemotaxis of neutrophils in freshly isolated human whole blood. CCX168 produced 50% inhibition (IC_{50}) of C5a-mediation neutrophil migration in this assay at a concentration of 1.7 nM; 90% inhibition (A_{10} value) was determined in human whole blood at a CCX168 concentration of 15.4 nM. CCX168 also inhibits C5aR in cynomolgus monkeys with a potency similar to that observed with human whole blood. However, CCX168 lacks affinity for mouse, rat or dog C5aR (IC_{50} > 10 µM).

CCX168 displays greater than 10,000-fold selectivity for hC5aR relative to other chemotactic receptors, including CCR1, CCR2, CCR4, CCR5, CCR6, CCR7, CCR8, CCR9, CCR10, CCR12, CXCR1, CXCR2, CXCR3, CXCR4, CXCR6, CXCR7, C5L2, C3aR, ChemR23, GPR1 and FPR1. CCX168 has been further evaluated against a panel of 55 unrelated receptors and membrane-associated proteins. Weak levels of activity (>1,000-fold selectivity relative to hC5aR) were noted against the site 2 sodium channel (59% inhibition with 10 µM CCX168).

1.2.1.2 Efficacy Models

CCX168 has been evaluated *in vivo* utilizing models that are relevant to the intended therapeutic use in humans. When C5a is generated locally in the bloodstream, C5aR-bearing leukocytes in the vicinity immediately upregulate adhesion molecules and adhere to the inner face of the blood vessel. If C5a is introduced systemically by intravenous injection, leukocyte adherence occurs immediately throughout the vasculature and, as a result, the number of leukocytes still flowing in the bloodstream drops transiently by a substantial amount. In general, evaluation of C5aR antagonists in animal models poses a challenge because C5aR antagonists that are potent for human C5aR, including CCX168, are less potent for C5aR orthologs in most other model species (such as mice, rats and rabbits). For this reason, CCX168 has been evaluated in C5a-induced leukopenia models using transgenic mice in which the mouse C5aR gene has been replaced with the human C5aR gene and non-human primates.

ChemoCentryx generated a human C5aR knock-in (hC5aR KI) mouse strain in which the mouse C5aR gene has been replaced with the human C5aR gene. The innate immune cells of these mice respond normally to C5a, in a manner highly sensitive to CCX168. *In vitro*, CCX168 blocks hC5a-mediated chemotaxis of leukocytes freshly isolated from these hC5aR KI mice with a potency (IC_{50} = 0.5 nM in 100% mouse plasma). This value is nearly identical to the potency (1.7 nM) exhibited by CCX168 in its inhibition of neutrophil migration to hC5a in whole human blood, indicating that the hC5aR KI mice are suitable for pharmacodynamic

evaluation of CCX168. In the human C5aR knock-in mice, an intravenous dose of 20 µg/kg hC5a robustly induces this leukopenia within one minute after injection. Pretreatment of the mice with an oral dose of 0.3 mg/kg CCX168, which resulted in a plasma concentration of approximately 75 nM at 60 min. post-dose, almost completely blocked the C5a-induced leukopenia. A dose of 0.03 mg/kg CCX168, producing a plasma concentration of 15 nM, resulted in a 50% reduction in the C5a-induced leukopenic response.

In cynomolgus monkeys, it was determined that an intravenous hC5a dose of 10 µg/kg robustly induces a drop in neutrophils (neutropenia) within one minute. Pre-treatment of the cynomolgus monkeys with a 30 mg/kg oral dose of CCX168 completely blocked the C5a-induced neutropenia. This dose of CCX168 resulted in a plasma concentration of approximately 230 nM at the time of hC5a administration. A dose of 3 mg/kg resulted in greater than 50% reduction of the hC5a response, an effect that was associated with a CCX168 plasma concentration of approximately 38 nM.

These mechanism-based pharmacology studies, taken together, support our estimate that maintaining human plasma CCX168 concentrations sufficiently high to provide ≥ 90% receptor coverage will provide significant clinical benefits in inflammatory conditions associated with C5aR activation.

1.2.2 Non-Clinical Safety and Toxicology

The toxicology program was designed to support this initial clinical Phase 1 study to assess the safety, tolerability and pharmacokinetics of CCX168 in healthy male volunteers. In this regard, a comprehensive toxicology program has been conducted in light of existing ICH nonclinical toxicology guidance (including M3 Nonclinical Safety Studies for the Conduct of Human Clinical trials for Pharmaceuticals; S2B Specific Aspects of Regulatory Genotoxicity Tests for Pharmaceuticals; and S7A Safety Pharmacology Studies for Human Pharmaceuticals).

The nonclinical toxicology program with CCX168 consisted of a series of acute (single-dose) and/or multiple-dose toxicology oral studies conducted in rats and cynomolgus monkeys. Oral repeat-dose range finding studies were subsequently followed by formal multiple-dose (28-day) studies utilizing the oral route of administration. The definitive 28-day studies involved the administration of CCX168 at daily doses up to 100 mg/kg/day in rats and 50 mg/kg/day in cynomolgus monkeys and included comprehensive clinical evaluations and the microscopic assessment of a full list of tissues. The multiple-dose cynomolgus study included electrocardiographic measurements. Toxicokinetic data was also collected in the multiple-dose rat and cynomolgus studies. The highest doses used in the definitive 28-day studies (up to 100 and 50 mg/kg/day, in rats and cynomolgus monkeys, respectively) represented Human Equivalent Doses (HED's) of 15.6 and 18.5 mg/kg/day, respectively. These doses are several orders of magnitude higher than the proposed starting clinical dose of 0.016 mg/kg (1 mg CCX168 for a 60-kg subject).

The effects of CCX168 upon the central nervous, respiratory, and cardiovascular systems were also assessed in single-dose stand-alone safety pharmacology experiments in rats and cynomolgus monkeys. An *in vitro* study to assess the potential effects of CCX168 upon hERG channel ionic conductance was also conducted. With regard to genotoxicity, *in vitro* bacterial (reverse mutation in histidine-requiring strains of *S. typhimurium* and tryptophan-requiring strains of *E. coli*) and mammalian (mutation at the thymidine kinase locus of mouse lymphoma L5178Y cells) mutagenicity tests were performed.

All toxicology studies and safety pharmacology studies with the exception of dose analyses performed in support of the acute toxicology and hERG studies were conducted in accordance with GLP regulations. A summary of the nonclinical toxicology, genotoxicology, and safety pharmacology studies performed in support of CCX168 are described in the following tables:

Overview of Toxicology Studies Performed with CCX168

Study Type	Method of Administration/Dosing Schedule	Species	Report No.	Doses
Acute	Oral /Single-Dose	Rat	PC0381_168_a<br>PC0381_168_b	0, 5, 25, 100 mg/kg
Repeat Dose	Oral / 7-Day	Rat	PC0374_168	0, 30, 100mg/kg
	Oral / 4 / (2) -day	Cynomolgus monkey	PC0383_168_a<br>PC0383_168_b	3, 50, 65, (80), (120) mg/kg
	Oral/28-Day	Rat	PC0384_168	0, 5, 25, 100 mg/kg
	Oral/28-Day	Cynomolgus monkey	PC0383_168	0, 5, 15, 50 mg/kg
Genotoxicity				
Ames	NA	*S. typhimurium* and tryptophan-requiring strains of *E. coli*	PC0378_168	Up to 5000 µg/plate
Mouse Lymphoma	NA	Mouse lymphoma cells	PC0379_168	Up to 500 µg/mL

Overview of Safety Pharmacology Studies Performed with CCX168

Study Type	Method of Administration/Duration	Species	Study No.	Doses
Central Nervous System	Oral/Single-Dose	Rat	PC0375_168	0, 5, 25, 100 mg/kg
Cardiovascular – *In vivo*	Oral/Single-Dose	Cynomolgus monkey	PC0377_168	0, 5, 15, 50 mg/kg
Cardiovascular – *In vitro* (HERG)	*In vitro*	Human cells transfected with human K-channel gene	PC0380_168	0.6, 1.2, 2.3 and 6.9 µM
Respiratory	Oral /Single-Dose	Rat	PC0376_025	0, 5, 25, 100 mg/kg

Single-dose and repeat-dose toxicology, safety pharmacology, and genotoxicity studies have been conducted with CCX168. Four-week definitive toxicology studies were conducted in rats and cynomolgus monkeys at CCX168 doses up to 100 and 50 mg/kg/day, respectively, a significant multiple above the proposed starting human dose of 0.016 mg/kg (1 mg for a 60-kg subject).

Based on in vivo safety pharmacology studies, which included a neuropharmacology study in rats, a pulmonary safety study in rats, and a cardiovascular study in conscious telemetered cynomolgus monkeys, there was no evidence of toxicity of CCX168. In an *in vitro* cardiovascular safety study, the IC_{50} value for hERG inhibition was determined to be > 2.3 µM, (the limit of solubility), further indicating CCX168 is unlikely to cause arrhythmias *in vivo*. Protein binding, red blood cell partitioning, hepatocyte metabolism, cytochrome P450 inhibition and induction, Caco-2 permeability and genotoxicity studies, including *in vitro* bacterial mutagenicity (Ames test) and *in vitro* mammalian cell mutagenicity (mouse lymphoma) studies, were also conducted and did not identify any safety concerns or significant potential for drug-drug interactions. In an acute toxicology study, single doses of CCX168 up to 100 mg/kg in rats produced no remarkable effects.

CCX168 was well tolerated in 28-day studies up to doses of 100 mg/kg in rats and 50 mg/kg in cynomolgus monkeys. There were no significant toxicological findings of concern in these studies. At selected time points, minor but statistically significant differences existed in selected clinical pathology parameters between control and CCX168 treated rats. These included an increase in reticulocytes in 100 mg/kg/day recovery-phase females, an increase in prothrombin time in males given doses > 25 mg/kg/day and a minimal increase in ALT levels on Day 30 in females administered 100 mg/kg/day. These differences were not clearly test article-related and not considered to be of toxicological importance because of their small magnitude, mean and/or individual values falling within the range of normal variability and/or reversibility of the finding following a 2-week treatment-free period.

Given the proposed starting clinical dose and cautious dose-escalation scheme to be employed in the Phase 1 trial, the risk for serious or unanticipated untoward events to occur in the proposed clinical trial based on the collective animal data is considered low.

1.2.3 Non-Clinical ADME

The pharmacokinetic behavior of CCX168 has been assessed in female CD-1 mice, male Sprague-Dawley rats, male beagle dogs, and male cynomolgus monkeys through intravenous (i.v.) and/or oral (p.o.) dosing and the data are summarized in the table below. Following intravenous dosing, the compound has a moderate to medium total body clearance in mice, rats and dogs (30 - 50% of liver blood flow). The terminal elimination half-life is moderate to long, at ca. 2 h in mice and rats, and 14.2 h in dogs, while the volume of distribution is moderate to medium (1.5, 2.5, and 4.7 L/kg for mice, rats and dogs, respectively). Following oral dosing in mice and rats, CCX168 is readily absorbed, showing moderate bioavailability for the aqueous hydroxypropyl methylcellulose (HPMC) suspension and high bioavailability for the PEG-400/Solutol HS-15 solution.

Pharmacokinetic Parameters of CCX168 in CD-1 Mice, Sprague-Dawley Rats, Beagle Dogs, and Cynomolgus Monkeys after Administration of a Single Oral Gavage or an Intravenous Dose of CCX168

a. Intravenous dosing

Parameter	Mouse	Rat	Dog
**dose (mg/kg)**	0.5	0.5	0.5
**Formulation**	Ethanol / *N,N*-dimethylacetamide / propylene glycol / 0.9% saline (10:10:30:75)	N,N-dimethylacetamide / ethanol / propylene glycol (31.6:36.8:31.6)	propylene glycol / *N,N*-dimethylacetamide / water (31.6/31.6/36.8)
**N =**	9 [a]	2	3
**CL [mL/min/kg]**	26.6	21.2	11.9
**$t_{1/2}$ [h]**	1.8	1.9	14.2
**Vd_{ss} [L/kg]**	1.5	1.8	4.7

[a] Non-serial blood sampling was used and a composite PK profile was obtained using the mean concentration at each time point

b. Oral dosing

Parameter	Mouse		Rat		Monkey
**p.o. dose (mg/kg)**	2	30	2	30	100
**Formulation**	1% HPMC (suspension)	PEG-400/solutol HS-15 (70:30) (solution)	1% HPMC (suspension)	PEG-400/Solutol HS-15 (70:30) (solution)	PEG-400 / solutol HS-15 (70:30) (solution)
**N =**	9 [a]	9 [a]	2	3	3
**C_{max} [ng/mL]**	75	4630	152	2530	3500
**AUC [ng•h/mL]**	240	18600	464	24600	33300
**$t_{1/2}$ [h]**		5.6	2.3	4.6	6.0
**t_{max} [h]**	1.0	1.0	1.0	1.5	4.0
**F [%]**	17	87	27	104	-

[a] Non-serial blood sampling was used and a composite PK profile was obtained using the mean concentration at each time point
i.v. = intravenous
p.o. = oral
C_{max} = maximum concentration
AUC = area under the concentration-vs.-time curve
CL = total body clearance

$t_{½}$ = terminal half-life
Vd_{ss} = volume of distribution at steady state
T_{max} = time of peak concentration
F = bioavailability

CCX168 displays moderate to medium *in vitro* metabolic turnover in cryo-preserved mouse, rat, and dog hepatocytes and low to moderate turnover in human hepatocytes. This result generally correlates well with the observed *in vivo* clearance in mice, rats and dogs and predicts a low to moderate clearance in humans. *In vitro* metabolism of CCX168 is primarily through monohydroxylation in monkey and human hepatocytes and through monohydroxylation, dealkylation and glucuronidation in rat and dog hepatocytes.

The compound is very highly protein bound in mouse, rat, dog and human plasma, at ca. 99% or higher, with corresponding unbound fractions at ca. 1% or lower. CCX168 has a low metabolism-mediated drug-drug interaction potential as a perpetrator, as its inhibition against major human cytochrome P450 isoforms is minimal (negligible for CYP1A2, 2C9, 2C19, and 2D6, and moderate for CYP3A4) and it shows no CYP3A4 and CYP2B6 induction potential at 10 μM in a human hepatocyte cytochrome P450 assay.

1.3 Prior Human Experience

This is a first-in-human clinical trial.

1.4 Rationale for the Study

CCX168 has potential in the treatment of patients with vasculitis or SLE. Since this is the first study in humans, the rationale for the study is to evaluate the safety, tolerability, PK and PD profiles of CCX168 after oral administration of single and multiple escalating doses of CCX168.

2 OBJECTIVES

2.1 Primary Objective

The primary objective of this study is to evaluate the safety and tolerability of single and multiple oral doses of CCX168, over a range of dose levels, in healthy male and female subjects.

2.2 Secondary Objectives

The secondary objectives of this study are to evaluate the:

- Single and multiple dose pharmacokinetic profile of CCX168 over a range of dose levels,
- Relationship between CCX168 plasma concentrations and C5aR-dependent CD11b upregulation in circulating neutrophils, and
- Relationship between CCX168 plasma concentrations and C5aR-dependent cell migration in a whole blood migration assay.

3 STUDY DESIGN

Up to 40 healthy male or female subjects will complete this double-blind, placebo-controlled, dose escalation study. This is the first time that CCX168 will be administered to humans. There will be two study periods. During Period 1, subjects will be enrolled in serial cohorts of eight subjects each. Within each cohort, six subjects will be randomly assigned to receive a single dose of CCX168 and two subjects will be randomly assigned to receive a single dose of placebo, except for Cohort 1 in which five subjects will receive CCX168 and three placebo. The reason for having three placebo subjects in Cohort 1 is to blind each of the three sequential steps of dosing of Cohort 1 (see below). Up to five sequential cohorts of subjects may receive increasing single doses of CCX168. Dose escalation will continue as planned unless safety or tolerability issues appear that prevent further dose escalation. During the second study period, subjects will receive the same dose as during the first period but once daily for a period of 7 days continuously. The lowest dose cohort in Period 2 will only be dosed after the same dose and two higher doses have been administered in the single-dose study period (Period 1). Furthermore, dose escalation in Period 2 will only occur if the preceding lower dose given daily for 7 days has been found safe.

The first dose cohort in Period 1 will be dosed in a staggered way so that 2 of the 8 subjects will be dosed on the first day (one randomized to placebo and one randomized to CCX168), the third and fourth subjects will be dosed at least 48 hours after the first 2 subjects have been dosed (one randomized to placebo and one randomized to CCX168), and the rest of the subjects (4 of 8) will be randomized 3:1, CCX168:placebo and dosed at least 48 hours after the first 4 subjects have been dosed. This staggered dosing of the first cohort follows the European Medicines Agency *Guideline on Strategies to Identify and Mitigate Risks for First-in-Human Clinical Trials with Investigational Medicinal Products* (2007). The subjects in the rest of the dose cohorts will be dosed with 10-minute intervals between dosing of each subject to prevent any precipitous adverse events from affecting the full dose cohort. This 10-minute interval is justified because delayed acute toxicity has not been observed with CCX168 in the toxicology studies and there is no scientific reason to believe it would occur in humans.

Dose escalation in Period 1 will occur only after review by the Investigator and the Sponsor’s Medical Monitor of all available PK data, and of safety data for at least three days after the initial dosing from all subjects in each of the preceding cohort(s). The National Cancer Institute (NCI) Common Terminology Criteria (CTC) for Adverse Events v3.0 (Appendix 11.3) will be used to assess dose-limiting toxicities. Occurrence of the same Grade 1 or higher CTC scale toxicities in two or more subjects in any cohort will be considered possible evidence of dose-limiting toxicity. If the adverse events are considered possibly related to study drug, the following options will be considered: cease dosing, administer a dose lower than that received by the prior cohort, or repeat a previous dose level. Occurrence of a Grade 2 or higher toxicity in one subject in any cohort will be considered evidence of possible dose-limiting toxicity. If the adverse events are considered possibly related to study drug, the following options will be considered: cease dosing, administer a dose lower than that received by the prior cohort, or repeat a previous dose level. In the absence of dose-limiting adverse events, dose escalation will continue up to a maximum dose of 100 mg (single dose). Clinical judgment must be exercised for any clinically significant toxicity, which may trigger the actions described above (cease dosing, administer a dose lower than that received by the prior cohort, or repeat a previous dose level).

Dose escalation in Period 2 will only occur after the next two higher single doses have been tested and found to be safe, for example, 1 mg CCX168 will only be tested in the multi-dose

period after a single 1, 3, and 10 mg CCX168 dose has been tested and found to be safe. Furthermore, dose escalation in Period 2 will only occur if the preceding lower dose given daily for 7 days has been found safe. The same CTC criteria and stopping rules of Period 1 apply to the Period 2 dosing.

It is planned that the subjects who complete Period 1 also participate in Period 2 to determine the safety and tolerability, PK and PD profiles of CCX168 when given daily for 7 days. If a subject who participated during Period 1 decided not to participate in Period 2, the subject will be replaced for Period 2. Treatment allocation for the subjects participating in the Period 2 evaluation (i.e., subject assignment to active or placebo treatment) and dose will be identical to the Period 1 assignment, e.g., subjects assigned to receive a 3 mg single dose of CCX168 during Period 1 will receive 3 mg CCX168 daily for 7 days during Period 2. Replacement subjects will receive the same treatment as the subjects who they replace.

In Period 1, subjects will remain at the study center for at least 24 hours after administration of study medication, during which time they will be observed closely for safety and blood samples will be collected for PK and PD analysis. Subjects will return to the study center on Study Days 3, 4, and 8 for collection of blood samples and for safety monitoring. All subjects will return to the study center for a second treatment period following a wash-out period of at least fourteen days from the Period 1 dosing. For Period 2, subjects will remain at the study center for the duration of the 7-day dosing period. During this time, subjects will be observed closely for safety and blood samples will be collected for PK and PD analysis. Subjects will be discharged from the study center on Day 8, and will return to the study center on Days 9, 10, and 15 for collection of blood samples and for safety monitoring. Subjects will be terminated from the study at the completion of the telephone contact on Study Day 29 of their second treatment period.

To the extent possible, any adverse events that are deemed study drug-related and are ongoing at discharge will be followed-up to resolution or until a determination is made that the unresolved event is stable.

4 STUDY POPULATION

4.1 Size of the Population

Approximately 40 subjects will be enrolled. Both men and women are eligible.

4.2 Inclusion Criteria

Subjects must meet all of the following inclusion criteria in order to enter the study:

1. Male or female subjects, aged 19-45 years inclusive, who are in generally good health, whose body mass index is 19 to 29 kg/m^2;
2. Willing and able to give written Informed Consent and to comply with the requirements of the study protocol;
3. Negative result of the human immunodeficiency virus (HIV) screen, the hepatitis B screen, and the hepatitis C screen;
4. Judged to be healthy by the Investigator, based on medical history, physical examination (including electrocardiogram [ECG]), and clinical laboratory assessments. Subjects with clinical laboratory values that are outside of normal limits and/or with

other abnormal clinical findings that are judged by the Investigator not to be of clinical significance may be entered into the study, and

5. Female subjects of childbearing potential, and male subjects with partners of childbearing potential, may participate if adequate contraception is used during, and for at least the four weeks after, any administration of study medication. Adequate contraception is defined as usage by at least one of the partners of a barrier method of contraception, together with usage by the female partner, commencing at least three months prior to Screening, of a stable regimen of any form of hormonal contraception or an intra-uterine device. Use of abstinence alone is not considered adequate. Use of a barrier method alone is considered adequate only if the male partner was vasectomized at least six months prior to Screening. Use of a double-barrier method of contraception is acceptable. Women who are not of childbearing potential may participate if they are surgically sterile or if they are at least two years post menopause.

4.3 Exclusion Criteria

1. Women who are pregnant, breastfeeding, or have a positive serum pregnancy test at Screening and/or on Study Day -1;
2. Expected requirement for use of any medication (with the exception of continuing use by female subjects of hormonal contraceptives in accordance with a regimen that has been stable for at least the three months prior to Screening) during the study period;
3. History within the three months prior to study entry of use of tobacco and/or nicotine-containing products;
4. History within one year prior to study entry of illicit drug use;
5. History of alcohol abuse at any time in the past;
6. History of any form of cancer;
7. Consumed alcoholic beverages, or any food or drink containing grapefruit or grapefruit juice within 24 hours of screening;
8. History or presence of any medical condition or disease which, in the opinion of the Investigator, may place the subject at unacceptable risk for study participation;
9. Donated or lost more than 350 mL of blood or blood products within 56 days prior to screening, or donated plasma within 7 days of randomization;
10. Subject's hemoglobin less than 12 g/dL (or less than 7.45 mmol/L);
11. Participated in any clinical study of an investigational product within 30 days prior to randomization;
12. Subject has any evidence of hepatic disease; AST, ALT, GGT, alkaline phosphatase, or bilirubin > 1.5 x the upper limit of normal;

13. Subject has any evidence of renal impairment; serum creatinine > 1.5 x upper limit of normal, and

14. Subject's urine tested positive at Screening and/or on Study Day -1 for any of the following: opioids, amphetamines, cannabinoids, benzodiazepines, barbiturates, cocaine, cotinine, or alcohol (Breathalyzer test allowed for alcohol).

4.4 Removal of Subjects from Therapy of Assessment

Subjects may be terminated early from the study for any of the following reasons:

1. Subject request. Subjects may withdraw their consent to participate in the study at any time without prejudice.

2. Investigator request. The Investigator may withdraw a subject if, in his/her clinical judgment, it is in the best interest of the subject or if the subject cannot comply with the protocol.

3. Sponsor request.

4. If a subject's hemoglobin drops below 11 g/dL (6.83 mmol/L), s/he may be dropped from the study at the Principal Investigator's discretion.

In the event of early withdrawal during Period 1, the tests and evaluations listed for Study Day 8 should be carried out whenever possible. In the event of early withdrawal during Period 2, the tests and evaluations listed for Study Day 15 should be carried out whenever possible. The Sponsor should be notified of all study withdrawals in a timely manner. Subjects who discontinue during Period 1 may be replaced to ensure that at least 6 subjects complete Period 1 at each dose level. Subjects who discontinue after Period 1 but before Period 2 will also be replaced. Subjects who discontinue during Period 2 will not be replaced. Replacement subjects will receive the same treatment of the subjects they replace.

5 STUDY MEDICATION/TREATMENT

5.1 Product Characteristics

CCX168 API will be provided by the Sponsor. CCX168 will be administered as a solution.

5.2 Randomization and Method of Treatment Assignment

Subjects will be enrolled in serial cohorts of eight subjects each. No more than five subjects within a cohort may be of the same gender. Within Cohort 1, five subjects will be randomly assigned to receive a single dose of CCX168 in Period 1 and then multiple doses in Period 2 and three subjects will be randomly assigned to receive a single dose of placebo in Period 1 and then multiple doses in Period 2. Within each subsequent cohort, six subjects will be randomly assigned to receive a single dose of CCX168 in Period 1 and then multiple doses in Period 2 and two subjects will be randomly assigned to receive a single dose of placebo in Period 1 and then multiple doses in Period 2. Up to 40 subjects will complete the study.

5.3 Doses and Regimens

CCX168 will be administered as a liquid solution for all dose cohorts in both treatment periods. Two formulations (designated A and B) will be tested in Period 1. Based on the PK profiles of

the two formulations, one will be selected for Period 2. CCX168 will be administered in accordance with the following plan:

- 1 mg CCX168 – Cohort 1
- 3 mg CCX168 – Cohort 2
- 10 mg CCX168 – Cohort 3
- 30 mg CCX168 – Cohort 4
- 100 mg CCX168 – Cohort 5

Within each cohort of eight subjects, six subjects will be assigned to receive CCX168, except for Cohort 1, in which five subjects will receive CCX168. All doses of CCX168 will be administered orally, on a double-blind basis. CCX168 will be dissolved and administered as dosing solutions prepared by the study pharmacist according to detailed instructions provided by the Sponsor. In Period 1, half of the subjects in each dose cohort (4 of 8) will receive formulation A and the other half will receive formulation B. Three of the six subjects randomized to CCX168 in each dose cohort will receive formulation A and the other three will receive formulation B. One of the two subjects randomized to placebo will receive formulation A and the other one will receive formulation B. For the first dose cohort (1 mg CCX168), the first two subjects (one randomized to placebo and one randomized to CCX168) will receive formulation A, the third and fourth subjects (one randomized to placebo and one randomized to CCX168) will receive formulation B. Two of the last four subjects of the cohort will receive formulation A randomly regarding treatment group assignment and the other two will receive formulation B randomly regarding treatment group assignment. This will be done to maintain the study blind.

The study pharmacist will prepare all dosing solutions according to detailed instructions provided separately. Subjects will fast (with the exception of water) beginning at least ten hours prior to administration of study medication and continuing until at least four hours after administration of study medication for both treatment periods. Administration of dosing solutions will be followed by administration of 30 mL of water. Water will be allowed as desired, except for the 1-hour period before and after study drug administration.

For all dose cohorts, CCX168 study drug will be dissolved in solution prior to dosing as shown in the table below:

CCX168 Dose (mg)	CCX168 Dosing Volume (mL)	CCX168 Dosing Concentration (mg/mL)
1	12	0.08
3	12	0.25
10	12	0.83
30	12	2.5
100	12	8.3

CCX168 Dose (mg)	CCX168 Dosing Volume (mL)	CCX168 Dosing Concentration (mg/mL)
Placebo	12	0

Subjects assigned to the placebo group will receive matching volumes of dosing solution without CCX168. The study pharmacist will prepare the placebo solutions according to detailed instructions provided separately.

Following administration of the dosing solutions, subjects will drink 30 mL of water.

All subjects will fast (with the exception of water) beginning at least ten hours prior to administration of study medication and continuing until at least four hours after administration of study medication. Water will be allowed as desired, except for the 1-hour periods before and immediately after study drug administration.

For Period 2, all subjects will receive either CCX168 or placebo once daily (at the same time each day) for 7 days continuously. Subjects will fast for at least 10 hours before dosing and 4 hours after dosing on each of the 7 days. Water will be allowed as desired, except for the 1-hour periods before and immediately after study drug administration.

5.4 Rationale for Dose Selection

The proposed starting dose (1 mg CCX168) for the Phase 1 clinical study is expected to be safe, based on toxicological (28-day NOAEL levels) and biological (MABEL approach) considerations, as described below.

Standard inter-species allometric scaling factors were applied to the NOAEL values (≥ 100 mg/kg in rats and ≥ 50 mg/kg in cynomolgus monkeys) derived from the 28-day toxicology studies, in order to conservatively select a safe starting clinical dose. The NOAEL values in rat and cynomolgus monkeys equate to a human equivalent dose (HED) of approximately ≥ 15.6 and ≥ 18.5 mg/kg/day, respectively. Application of the usual 10-fold additional safety margin to account for possible inter-species differences in toxicity, would result in the most conservative starting human dose of 1.5 mg/kg (about 90 mg for a 60-kg subject). Thus, the proposed starting dose of 0.016 mg/kg (1 mg) in the Phase 1 study is approximately 90 times lower than the safe starting human dose derived from the animal toxicology studies on an HED basis.

The proposed starting human dose of 1 mg dose is expected to produce a plasma AUC_{0-24} of about 30 ng.hr/mL, based on allometric scaling of the pharmacokinetic behavior of CCX168 in mice, rats and dogs. Even after application of an additional 10-fold safety margin, the proposed starting human dose is at least 30 times lower than the most-conservative starting dose that could be justified based on CCX168 exposure in the 28-day toxicology studies.

Calculation of the MABEL was based on an understanding of the pharmacological effects of CCX168 in the cynomolgus model of C5a-induced leucopenia. The lowest dose associated with a pharmacological effect in this model was 3 mg/kg. On an HED basis, this dose translates into 1 mg/kg in humans. The proposed starting human dose of 1 mg (0.016 mg/kg for a 60-kg subject) is approximately 60 times lower than this MABEL.

In summary, the proposed starting human dose (1 mg) of CCX168 for the Phase I study is predicted to be at least 30 times lower than the most conservative estimation of a safe human

starting dose, which in this case results from analysis of plasma exposure associated with the 28-day NOAEL in monkeys.

The planned dose escalation schedule was developed based on safety considerations. Increasing the 1 mg starting dose by half-log increments for the subsequent cohorts is considered to be safe and consistent with industry norms for dose escalation. The dose escalation schedule allows for exploration of doses likely to produce blood levels covering the therapeutic range, estimated to be about 10 to 30 mg once daily. Given the proposed starting clinical dose and cautious dose-escalation scheme to be employed in the Phase 1 trial, the risk of occurrence of serious or unanticipated untoward events in the proposed clinical trial is considered low.

5.5 Drug Supply

5.5.1 Packaging and Labeling

CCX168 API will be packaged in a high density polyethylene (HDPE) bottle and provided to the study pharmacist. The bottle will be labeled to indicate product name, lot number, and manufacturer.

5.5.2 Storage

CCX168 API will be stored at 15°C to 25°C (59°F to 77°F).

The API dosing solutions and placebo dosing solutions are to be prepared fresh daily and should be discarded after 24 hours of storage at 15°C to 25°C (59°F to 77°F).

5.6 Blinding

Within each cohort of eight subjects, six subjects will be assigned randomly to receive CCX168 and two will receive placebo, except for Cohort 1 in which five subjects will receive CCX168 and three will receive placebo. All doses of CCX168 will be administered orally, on a double-blind basis. CCX168 will be dissolved and administered as dosing solutions. In order to blind the study drug, matching volumes of dosing solution without CCX168 added will be given to the placebo group. Only the study pharmacist at the study site and his/her designees will have access to the treatment code, as well as the analysts performing the PK and PD assays. Unblinded PK and PD data will not be shared with the study subjects, investigative staff, or sponsor personnel conducting the clinical part of the study, until the study database has been locked and treatment codes made available.

5.7 Drug Accountability

The study pharmacist and investigator must maintain accurate records of dates and quantities of product(s) received, to whom dispensed (subject-by-subject accounting), and accounts of any product accidentally or deliberately destroyed. The pharmacist will also retain 10-mL samples of the dosing solutions at -70°C for subsequent analysis, if deemed necessary. The Investigator must retain all unused and/or expired study supplies until the study monitor has confirmed the accountability data.

5.8 Treatment Compliance

The CCX168 and placebo doses will be administered in the presence of study site personnel to ensure compliance. Any events of non-compliance to the protocol will be documented in the study records.

5.9 Concomitant Medications and Restrictions

Use of the following medications is prohibited:

- Prescription medications used within 14 days prior to randomization and/or during the study period, with the exception of hormonal contraceptive use by female subjects in accordance with a regimen that has been stable for at least the three months prior to Screening and that remains unchanged throughout the study period.
- Over-the-counter medications and herbal supplements.

All concomitant medications taken during the course of the study will be recorded on the concomitant medication pages of the CRF.

Use of the following is restricted:

- Beverage alcohol, caffeine, grapefruit-containing products, Seville oranges, and marmalade must not be consumed beginning at least 48 hours prior to randomization and continuing throughout the blood sample collection period.
- Poppy seeds/foods containing poppy seeds must not be consumed for at least the 24 hours prior to urine sampling for screening for drugs of abuse.

Subjects will fast (with the exception of water) beginning at least ten hours prior to administration of study medication and continuing until at least four hours after administration of study medication. Lunch will be given approximately 4 hours, a snack approximately 8 hours and dinner approximately 11 hours after dosing on each day.

Subjects should refrain from strenuous physical exercise for at least 4 days prior to first study drug administration and also during the study.

6 STUDY PROCEDURES

6.1 Screening and Enrollment

Informed Consent must be obtained prior to performance of any study-specific tests or evaluations. Within 21 days prior to study entry, subjects will undergo the following evaluations to determine their eligibility for study participation.

- Demographics, medical history, and concomitant medication usage will be recorded.
- A complete physical examination (excluding genitourinary, ophthalmic, and female breast assessments) will be performed; body weight, height, and body mass index will be determined.
- Vital signs (temperature, orthostatic blood pressure, heart rate, and respiration rate) will be assessed, using study center normal ranges.

- A 12-lead ECG will be recorded digitally and saved for analysis.
- Samples will be collected for routine clinical laboratory tests, including blood chemistry, hematology, coagulation tests, urinalysis, and virology, as detailed in Section 7.2.2.
- Samples will be collected for a urine screen for cotinine, drugs of abuse, and alcohol. Alcohol screening may be done by Breathalyzer test.
- For all women, a serum pregnancy test will be administered.

6.2 Study Conduct

Subjects will be screened within 21 days prior to study entry. Subjects will be admitted to the study center on Study Day -1 of Period 1. On Study Day 1 of Period 1 subjects will receive a single dose of study medication. Subjects will remain at the study center for at least 24 hours after administration of study medication, during which time they will be observed closely for safety and blood samples will be collected for PK and PD analysis. Subjects will return to the study center on Study Days 3, 4, and 8 for collection of blood samples and for safety monitoring. Visits for Study Days 2, 3, 4, and 8 must occur on the scheduled days.

All subjects will return to the study center for a second treatment period following a wash-out period of at least fourteen days from first dosing. For Period 2, subjects will remain at the study center for the duration of the 7-day dosing period. During this time, subjects will be observed closely for safety and blood samples will be collected for PK and PD analysis. Subjects will be discharged from the study center on Day 8, and will return to the study center on Days 9, 10, and 15 for collection of blood samples and for safety monitoring. Study Days 9, 10, and 15 must occur on the scheduled days. Subjects will be terminated from the study at the completion of the telephone contact on Study Day 29 of their second treatment period. The telephone contact on Study Day 29 could occur within +/- 2 days of the scheduled day.

To the extent possible, any adverse events that are deemed study drug-related and are ongoing at discharge will be followed-up to resolution or until a determination is made that the unresolved event is stable.

Refer to the Time and Events tables for a complete schedule of study procedures.

7 STUDY ASSESSMENTS

7.1 Efficacy Assessments

There are no efficacy assessments in this study.

7.2 Safety Assessments

7.2.1 Physical Examinations and Vital Signs

During Period 1, a complete physical examination (including evaluation of general appearance/mental status, HEENT [head, eyes, ears, nose, throat], and the following body systems: dermatologic, cardiovascular, respiratory, gastrointestinal, musculoskeletal and neurologic) will be performed at Screening and Study Day 8 for all subjects. A Body System review will be done on Days -1, 1, 2, 3, and 4.

During Period 2, a complete physical examination (including evaluation of general appearance/mental status, HEENT [head, eyes, ears, nose, throat], and the following body systems: dermatologic, cardiovascular, respiratory, gastrointestinal, musculoskeletal and neurologic) will be performed at Study Day -1, 2, 8, and 15 for all subjects. A Body System review will be done on Days 1, 3, 4, 5, 6, 7, 9, and 10.

Vital signs will be measured during Screening and on each study day during Periods 1 and 2, except for the telephone visit, as indicated in the Time and Events tables. Blood pressure (orthostatic for the first 24 hours of Period 1), pulse rate, respiration rate, and oral temperature will be measured in accordance with the study site's standard operating procedures. All assessments will be performed while the subject is in the supine position and has rested for at least three minutes.

Twelve-lead ECG measurements will be acquired and stored digitally at the time points specified in the Time and Events tables.

7.2.2 Clinical Laboratory Assessments

The following tests will be performed by the Study Site's Central Laboratory at the visits identified in the Time and Events tables.

- Hematology: hemoglobin, hematocrit, red blood cell (RBC) count, white blood cell (WBC) count with differential, platelet count, mean cell hemoglobin (MCH), mean cell hemoglobin concentration (MCHC), mean corpuscular volume.
- Serum Chemistry: liver panel (bilirubin, lactate dehydrogenase [LDH], SGOT/AST, SGPT/ALT), renal panel (BUN, creatinine), creatinine phosphokinase (CPK), albumin, sodium, potassium, bicarbonate, chloride, calcium, inorganic phosphorus, glucose, total proteins, alkaline phosphatase, γ-glutamyl transpeptidase, cholesterol, uric acid.
- Coagulation: prothrombin time, activated partial thromboplastin time, INR.
- Urinalysis: pH, specific gravity, glucose, protein, nitrite, ketones, bilirubin, blood, urobilinogen, RBC, WBC.
- Virology (measured only at Screening): hepatitis B surface antigen, hepatitis C antibodies, HIV 1 and 2 antibodies.
- Urine Screen for Drugs of Abuse and Tobacco Use: opioids, amphetamines, cannabinoids, benzodiazepines, barbiturates, cocaine, cotinine, and alcohol. Alcohol test may be done by Breathalyzer test.
- Serum pregnancy (measured only at Screening): for women of childbearing potential; the serum pregnancy test will be repeated on Study Day -1, if it is more than 14 days after the Screening pregnancy test.

At each specified time point in accordance with the Time and Events tables, blood will be collected into an EDTA tube for hematology, into an SST tube for serum chemistry and for pregnancy test (if applicable), into a citrate tube for coagulation testing, and into a plain tube without additives for virology measurements. Urine will be collected in a universal container for urinalysis, and for the urine screen for drugs of abuse and cotinine.

7.2.3 Reporting of Adverse Events

An adverse event (AE) is defined as any untoward medical occurrence in a subject participating in a clinical trial who is administered an investigational product, at any dose; the adverse event does not necessarily have to have a causal relationship with this product. An adverse event could therefore be any unfavorable and/or unintended sign (including abnormal laboratory findings), symptom, or disease temporally associated with the use of an investigational product, whether or not considered related to the investigational product. This definition includes intercurrent illnesses or injuries, and exacerbation of pre-existing conditions.

An unexpected adverse event is an adverse event that is not identified in nature, severity, or frequency in the current Clinical Investigator's Brochure, or that is of greater severity than expected based on the information in the Clinical Investigator's Brochure.

All adverse events occurring in subjects who have been randomized to treatment will be recorded on the CRF and will be reported in accordance with regulatory requirements. Adverse events reported prior to commencement of administration of study medication will be considered pre-treatment events.

All adverse events will be monitored until resolution or, if the AE is determined to be chronic, until a cause is identified. If an adverse event remains unresolved at the conclusion of the study, a clinical assessment will be made by the Investigator and the Sponsor's Medical Monitor whether continued follow-up of the adverse event is warranted.

The severity of each adverse event will be determined by the Investigator using the National Cancer Institute (NCI) Common Terminology Criteria (CTC) for Adverse Events v3.0 (Appendix 11.3). For adverse events not listed in the CTC tables, the following scale will be used to determine the event's severity:

- Mild (Grade 1): no limitation of usual activities.
- Moderate (Grade 2): some limitation of usual activities.
- Severe (Grade 3): inability to carry out usual activities.
- Life-threatening (Grade 4): an immediate risk of death.
- Death (Grade 5)

The relationship of the study medication to an adverse event will be determined by the Investigator based on the following definitions:

- Probably Not Related: the adverse event was more likely explained by causes other than the study medication.
- Possibly Related: the study medication administration and the adverse event occurrence were reasonably related in time, and the AE was explained equally well by causes other than the study medication or was more likely explained by exposure to the study medication than by other causes.

A serious adverse event (SAE) is defined as any untoward medical occurrence that at any dose:

- Results in death.

- Is life-threatening (i.e., the patient was, in the opinion of the Investigator, at immediate risk of death from the event as it occurred).
- Requires or prolongs hospitalization.
- Results in persistent or significant disability or incapacity.
- Is a congenital anomaly or birth defect.
- Is an important and significant medical event that, based on appropriate medical judgment, may jeopardize the patient and/or may require medical or surgical intervention to prevent one of the other outcomes defining serious.

7.2.4 Reporting of Serious Adverse Events

Any serious adverse event, whether or not considered study related, will be reported immediately (within 24 hours) to the Sponsor's Medical Monitor. Any medication or other therapeutic measures used to treat the event will be recorded on the appropriate CRF page(s) in addition to the outcome of the adverse event.

7.3 Pharmacokinetic Assessments

Concentrations of CCX168 will be determined in plasma from 6-mL blood samples collected in K_2EDTA tubes at the following times during Period 1:

- Study Day 1: immediately prior to administration of study medication (Time 0), and at Hours 0.08, 0.25, 0.5, 1, 1.5, 2, 3, 4, 6, 10, and 12 after administration of study medication
- Study Day 2: at Hours 18, and 24 after administration of study medication
- Study Day 3: at approximately Hour 48 after administration of study medication
- Study Day 4: at approximately Hour 72 after administration of study medication
- Study Day 8: at approximately Hour 168 after administration of study medication

Concentrations of CCX168 will be determined in plasma from 6-mL blood samples collected in K_2EDTA tubes at the following times during Period 2:

- Study Day 1: immediately prior to administration of study medication (Time 0), and at Hours 0.08, 0.25, 0.5, 1, 1.5, 2, 3, 4, 6, 10, and 12 after administration of study medication
- Study Day 2: at Hours 18, and 24 after first administration of study medication
- Study Day 3: at approximately Hour 48 after first administration of study medication
- Study Day 4: at approximately Hour 72 after first administration of study medication
- Study Day 5: at approximately Hour 96 after first administration of study medication
- Study Day 6: at approximately Hour 120 after first administration of study medication

- Study Day 7: immediately prior to 7th administration of study medication (Time 144), and at Hours 144.08, 144.25, 144.5, 145, 145.5, 146, 147, 148, 150, 154, and 156 after first administration of study medication
- Study Day 8: at Hours 162, and 168 after first administration of study medication
- Study Day 9: at approximately Hour 192 after first administration of study medication
- Study Day 10: at approximately Hour 216 after first administration of study medication
- Study Day 15: at approximately Hour 336 after first administration of study medication

For the pharmacokinetic evaluations, a 6-mL blood sample will be collected into an appropriately labeled K_2EDTA tube at each specified time point during the study. The sample will be mixed gently and kept on wet ice until centrifuged (within 30 minutes after collection) at approximately 2000 x g, in a refrigerated centrifuge, for approximately 10 minutes. Resultant plasma will be split into three ~1-mL aliquots and transferred to three appropriately labeled polypropylene tubes and stored at approximately -70°C or below until analysis.

Total plasma concentrations of CCX168 will be determined using a validated analytical method.

All blood samples will be collected by either direct venipuncture or an indwelling cannula inserted into a forearm vein.

7.4 Pharmacodynamic Assessments

7.4.1 CD11b Assays

Blood samples will be collected from subjects in the 10 mg, 30 mg, and 100 mg dose cohorts to determine the relationship between CCX168 plasma concentrations and inhibition of C5aR-dependent upregulation of CD11b in peripheral blood neutrophils. In Period 1, the samples will be collected at pre-dose and Hours 2 and 24 after dosing. Depending on the results obtained from Period 1 samples, the pharmacodynamic samples might not be collected in Period 2. If required, the Period 2 samples will be drawn before the first dose of this period and Hour 146 (2 hours after the 7th daily dose), and Hour 168 (24 hours after the 7th daily dose). The time of the Hour 2 collection in Period 1 and Hour 146 in Period 2 may be adjusted for the 30 and 100 mg dose cohorts based on the actual T_{max} of CCX168 for the 10 mg CCX168 dose.

For the pharmacodynamic assessments one 25-mL sample will be collected into an appropriately labeled K_2EDTA tube at each specified time point during the study. Each sample will be mixed gently and kept at ambient temperature at all times (temperature range: 18 to 30 ºC). For the CD11b assay, a 5-mL aliquot is needed. This sample will be processed at the study site and will then be shipped to ChemoCentryx under cold but not freezing conditions (temperature range: 1 to 10 ºC).

7.4.2 Whole Blood Migration Assays

Blood samples will also be collected from subjects in the 10 mg, 30 mg, and 100 mg dose cohorts to determine the relationship between CCX168 plasma concentrations and inhibition of C5aR-dependent migration of peripheral blood neutrophils. In Period 1, the samples will be collected pre-dose and Hours 2 and 24 after dosing. Depending on the results obtained from Period 1 samples, the pharmacodynamic samples might not be collected in Period 2. If

required, the Period 2 samples will be drawn before the first dose of this period and Hour 146 (2 hours after the 7th daily dose), and Hour 168 (24 hours after the 7th daily dose). The time of the Hour 2 collection in Period 1 and Hour 146 in Period 2 may be adjusted for the 30 and 100 mg dose cohorts based on the actual T_{max} of CCX168 for the 10 mg CCX168 dose.

For the pharmacodynamic assessments one 25-mL sample will be collected into an appropriately labeled K_2EDTA tube at each specified time point during the study. Each sample will be mixed gently and kept at ambient temperature at all times (temperature range: 18 to 30 ºC). For the whole blood migration evaluations, a 20-mL aliquot is needed. The ability of the peripheral blood neutrophils to respond chemotactically to a dose range of C5a and control agonists (such as CXCL8 or fMLP) will be measured.

7.5 Study Completion and Withdrawal

Day 29 of Period 2 will be the last Study Day for all subjects. Procedures for this day will be completed per the Time and Events Table. The telephone visit may occur within a +/- 2-day window. For early withdrawals (prior to Day 8) during Period 1, the procedures scheduled for Study Day 8 will be performed. For early withdrawals (prior to Day 15) during Period 2, the procedures scheduled for Study Day 15 will be performed. Subjects who discontinue during Period 1 may be replaced to ensure that at least 6 subjects complete Period 1 at each dose level. Subjects who discontinue after Period 1 but before Period 2 will also be replaced. Subjects who discontinue during Period 2 will not be replaced.

7.6 Data Monitoring Committee

There will not be a Data Monitoring Committee for this study.

7.7 Statistical Methods

Statistical and Analytical Plan

All baseline subject characteristics of demographic data (age, gender, height, weight, race), smoking status, medical history (abnormalities only), physical examination (abnormalities only), ECG parameters, and concomitant medications at study entry will be listed. Demographics will be summarized. All clinical safety and tolerability data will be listed for each subject and by treatment. Individual vital signs and individual differences from baseline in vital signs will be listed by treatment and measurement time and summarized descriptively. Laboratory values outside the laboratory's normal ranges will be listed separately, together with associated repeats and comments as to their clinical significance. All reported adverse events will be coded using Medical Dictionary for Drug Regulatory Affairs (MedDRA). Treatment-emergent adverse events will be listed by subject, treatment and by other categorical information of interest, such as onset and resolution times, time of onset relative to dose, severity, causal relationship to study medication, action taken, and outcome. Adverse events will be regarded as "pre-treatment" if they occur between Screening and the time of administration of the first dose of study medication. All other adverse events will be considered "treatment-emergent". The incidence of treatment-emergent adverse events will be summarized by treatment, system organ class, and preferred term, and the incidence of adverse events will also be summarized separately by maximum severity and relationship to study drug. Adverse events leading to withdrawal and serious adverse events (if any) will be listed and summarized, if appropriate.

Categorical measurements will be summarized by n (number of subjects) and % (percentage of subjects). Continuous variables will be summarized at a minimum by N (number of subjects), mean, median, standard deviation, minimum, and maximum.

Pre-treatment medications and concomitant medications administered during the study will be listed.

Individual vital signs and ECG parameters (PR, QRS, QT, and QTc) will be listed by treatment and measurement time, and summarized using descriptive statistics as appropriate. Individual changes from baseline in vital signs and ECG parameters will be calculated, and will be summarized descriptively and by statistical parameters as appropriate. The incidence of subjects with QT and QTc intervals greater than 450 msec and with increase from baseline of >30 and >60 msec will be summarized.

Laboratory values outside the laboratory's normal ranges will be listed by treatment and measurement time, together with associated repeats and comments as to their clinical significance. Laboratory results will also be summarized by treatment group.

Data from all placebo subjects will be pooled for the summary tables.

Data from the two study Periods will be presented separately.

7.8 Subject Populations

7.8.1 Intent-to-Treat

This includes all subjects who have been randomized into the study.

7.8.2 Safety

All subjects who have been randomized and received at least 1 dose of study drug.

7.8.3 Evaluable

All subjects who have been randomized, received at least 1 dose of study drug, and have sufficient data to be included in the analysis.

7.9 Efficacy Endpoints

Not applicable

7.10 Safety Endpoints

Safety endpoints include adverse event incidence and clinically significant changes in laboratory, ECG, and vital sign parameters.

7.11 Pharmacokinetic Endpoints

The following parameters will be determined for Period 1 and for Days 1 and 7 of Period 2, where applicable:

C_{max}	Maximum plasma concentration
t_{max}	Time of maximum plasma concentration
λ_z	Terminal phase rate constant

$t_{½z}$	Apparent terminal half-life
CL/F	Apparent oral clearance
V_z/F	Apparent volume of distribution
CL_{ss}/F	Apparent oral clearance at steady-state
V_{zss}/F	Apparent volume of distribution at steady-state
$AUC_{0\text{-}t}$	Area under the plasma concentration-time curve from Time 0 to Time t (time of last quantifiable plasma concentration)
AUC_{inf}	Area under the plasma concentration-time curve from Time 0 to infinity
$AUC_{0\text{-}24}$	Area under the plasma concentration-time curve from Time 0 to 24h

In addition, the accumulation ratio ($AUC_{0\text{-}24}$ for Day 7 divided by Day 1) will also be calculated.

Dose-proportionality will be assessed using the "power model" approach based on the 90% confidence interval for the slope.

7.12 Pharmacodynamic Endpoints

The degree of C5aR occupancy by CCX168 for each subject at each time point will be determined based on the results from the CD11b-upregulation and migration experiments. Specifically, the endpoints will include either the percent inhibition of a C5a-induced signal in comparison to controls or else a measure of the dose ratio brought about by CCX168 on a C5a dose response in comparison to controls. This dose ratio will be given as an A_x value, where A_2 is the concentration of drug that gives rise to a two-fold shift in the agonist dose-response curve.

7.13 Statistical Analysis Methodology

7.13.1 Baseline Characteristics

Baseline characteristics and demographics will be listed by subject and summarized in tabular form.

7.13.2 Primary Efficacy Analysis

Not applicable

7.13.3 Secondary Efficacy Analyses

Not applicable

7.13.4 Covariates

The effect of age, gender, weight, and BMI on the PK and PD endpoints may be evaluated.

7.13.5 Safety Analyses

Summary statistics will be calculated for all safety parameters. No inferential statistical analysis will be done on safety parameters. All available safety data from each dose cohort will be reviewed before dose escalation. Adverse event and safety laboratory data from all preceding dose cohorts will be reviewed to determine if there are any adverse trends.

7.13.6 Pharmacokinetic Analysis

The pharmacokinetic analysis will be conducted using model-independent methods, and will be based on plasma concentrations of CCX168 from those subjects who have evaluable plasma concentration-time profiles.

Individual plasma concentrations of CCX168 will be listed by subject, sampling time, and dose group, and will be summarized descriptively by dose group using the arithmetic mean, standard deviation, median, minimum, and maximum.

Individual plasma concentration-time profiles of CCX168 will be plotted on both a linear and a logarithmic scale by dose group. Mean values +/- SD will be presented graphically for each cohort.

Nominal sampling times will be used in the pharmacokinetic analysis if these do not differ from actual times by more than 20%, in which case the actual times will be used. Values that are below the quantification limit (BQL) will be set to zero for the pharmacokinetic analysis, except that BQL values between two non-BQL values will be set to missing for pharmacokinetic parameter calculation. For the purpose of calculating AUC_{0-t}, when two consecutive BQL plasma concentrations are encountered after t_{max}, all subsequent values will be excluded from the analysis. Quantifiable concentrations at pre-dose will be examined to determine possible causes and may require re-examination of the analytical method.

Pharmacokinetic parameters will be listed by subject and treatment group. Descriptive summaries including arithmetic mean, standard deviation, coefficient of variation, minimum, maximum, median, geometric mean, and 95% confidence intervals will be presented.

The two formulations used in the single-dose period of the study will be compared with regard to AUC, C_{max}, and $t_{1/2}$ for the purpose of selecting a formulation for the multi-dose period of the study.

The following pharmacokinetic parameters will be determined for Day 1 and Day 7, where applicable:

C_{max} Maximum plasma concentration, obtained directly from the experimental observations without interpolation

t_{max} Time of maximum plasma concentration, obtained directly from the experimental observations without interpolation

λ_z Apparent terminal phase rate constant, estimated by linear regression of the log concentration-time data associated with the terminal phase of the plasma concentration-time profile. The number of data points included in the regression will be determined by visual inspection. A minimum of three data points in the terminal phase, excluding C_{max}, will be required to estimate λ_z.

$t_{1/2z}$ Terminal half-life, calculated as:

$$t_{\frac{1}{2}z} = \frac{log_e(2)}{\lambda_z}$$

CL/F Apparent oral clearance, calculated as dose/AUC_{0-inf}

CL_{ss}/F Apparent oral clearance at steady-state, calculated as dose/AUC_{0-24}

V_z/F Apparent volume of distribution, calculated as (dose/[λ_z* AUC_{0-inf}])

V_{ss}/F Apparent volume of distribution at steady-state, calculated as (dose/[λ_z* AUC_{0-24}])

AUC_{0-t} Area under the plasma concentration-time curve from Time 0 to Time t (time of last quantifiable plasma concentration), calculated using the linear trapezoidal rule

AUC_{inf} Area under the plasma concentration-time curve from Time 0 to infinity, calculated as:

$$AUC_{inf} = AUC_{0-t} + \left(\frac{C_{last}}{\lambda_z}\right)$$

where C_{last} is the last quantifiable concentration

AUC_{0-24} Area under the plasma concentration-time curve from Time 0 to Hour 24, calculated using the linear trapezoidal rule

Assessment of Accumulation

The degree of accumulation of CCX168 following administration of seven daily doses of CCX168 will be assessed statistically by comparing log transformed AUC_{0-24} on Study Days 1 and 7 using a mixed effects model with day as the fixed effect and subject as the random effect. Interaction between dose and day will be investigated, and taken into account if significant. The geometric least squares means (LS_{means}) for Study Days 1 and 7, the ratio of geometric LS_{means} (Study Day 7/Study Day 1), and a 90% confidence interval (CI) for the ratio, may be displayed.

Assessment of Dose Proportionality

If at least three dose levels are investigated in this study, a preliminary assessment of dose-proportionality of the pharmacokinetics of CCX168 will be performed.

Log-transformed AUC_{inf}, C_{max} and $t_{½}$ values will be derived, and a model of the form

Log (parameter) = Intercept + Subject + β*Log (Dose) + Error

where subject is a random term and dose is a fixed term, will be fitted to assess a between-subjects estimate of the slope in order to assess dose-proportionality. A point estimate of the slope β, with its 90% confidence interval, will provide a plausible range within which the true slope occurs. The interpretation of the slope will be such that a tentative conclusion of dose-proportionality for the dose-dependent parameters (AUC_{inf} and C_{max}; AUC_{0-24} will be used in place of AUC_{inf} if the latter is more than 20% extrapolated) will be made if the 90% confidence interval for the slope contains 1. For dose-independence of $t_{1/2}$, the 90% confidence interval should contain 0.

7.13.7 Pharmacodynamic Analysis

The degree of C5aR occupancy by CCX168 for each subject at each time point will be determined based on the results from the CD11b-upregulation and migration experiments. Specifically, the analysis will include a measure of CCX168 inhibition of C5a-induced signal in the CD11b assay. In addition, the analysis will include a dose ratio determined from the whole blood migration assay, where the A_2 values are calculated from the following equation:

$$pA_2 = p[\mathbf{drug(M)}] - p[(\mathbf{A'/A})\mathbf{-}1]$$

where **A** reflects the potency of the agonist in the absence of antagonist and **A'** reflects the potency of the agonist in the presence of antagonist at a given concentration of drug (**[drug(M)]**).

7.14 Sample Size Justification

This Phase 1 study will enroll a total of 8 healthy volunteers per dose cohort, 6 randomized to CCX168 and 2 to placebo, except for cohort 1 in which 5 subjects will receive CCX168 and 3 placebo. The sample size is based on practical and not statistical considerations. It is customary for Phase 1 studies to enroll a small number of subjects per dose cohort.

7.15 Protocol Deviations

Protocol deviations that could affect the outcome of the study will be listed and the potential impact on the study results discussed.

8 STUDY COMPLETION AND TERMINATION

8.1 Study Completion

A subject has completed the study when s/he has completed the study procedures per protocol.

8.2 Study Termination

The end of study is defined as the last study visit of the last clinical trial subject.

9 REGULATORY AND ADMINISTRATIVE REQUIREMENTS

9.1 Investigator Responsibilities

Prior to trial initiation, the Investigator will provide the Sponsor with a fully executed and signed FDA Form 1572 and a Financial Disclosure Form. Financial Disclosure Forms also will be completed for all Sub-Investigators listed on the Form 1572 who will be involved directly in the treatment or evaluation of research subjects in this trial.

The study will be conducted in accordance with the Declaration of Helsinki (amended by the 59th World Medical Association General Assembly, October 2008) and Good Clinical Practice (GCP) according to International Conference on Harmonisation (ICH) guidelines. Specifically, the study is based on adequately performed laboratory and animal experimentation; the study will be conducted under a protocol reviewed by an IRB/EC; the study will be conducted by scientifically and medically qualified persons; the benefits of the study are in proportion to the risks; the rights and welfare of the subjects will be respected; and each subject will give his/her informed consent before any protocol-specific tests or evaluations are performed.

9.2 Institutional Review Board or Ethics Committee

Prior to initiating the study, the Investigator will obtain written confirmation from the IRB/EC that the IRB/EC was properly constituted and compliant with all United States Food and Drug Administration (FDA) requirements and applicable local Regulatory requirements. A copy of the confirmation will be provided to the Sponsor. The Principal Investigator will provide the IRB/EC with all appropriate materials, including the protocol and Informed Consent documents. The trial will not be initiated until IRB/EC approval of the protocol, the Informed Consent document, and all recruiting materials are obtained in writing by the Investigator and copies are received by the Sponsor. Appropriate reports on the progress of the study will be made to the IRB/EC and the Sponsor by the Principal Investigator in accordance with applicable governmental regulations and in agreement with the policy established by the Sponsor.

9.3 Informed Consent

A properly executed, written, Informed Consent Form, in compliance with the Declaration of Helsinki, ICH GCP, and US Code of Federal Regulations for Protection of Human Subjects (21 CFR 50.25[a,b], CFR 50.27, and CFR Part 46, Subpart A), will be signed by each subject prior to entering the trial. The Investigator will provide a copy of the signed Informed Consent Form to each subject and will maintain a copy in the subject’s record file.

9.4 Protocol Modifications

Only the Sponsor may modify the protocol. Amendments to the protocol will be made only after consultation and agreement between the Sponsor and the Principal Investigator. The only exception is when the Investigator considers that a subject’s safety would be compromised without immediate action. In this circumstance, immediate approval of the chairperson of the IRB/EC must be sought, and the Investigator should inform the Sponsor’s Medical Monitor and the full IRB/EC within five working days after the emergency occurred. All other amendments that have an impact on subject risk or the study objectives, and/or that require revision of the Informed Consent Form, must receive approval from the IRB/EC prior to their implementation, except when the changes involve only logistical or administrative aspects of the trial. The IRB/EC must be notified of changes that are made to study contact personnel, but IRB/EC review or approval of these changes is not required.

9.5 Regulatory Documentation

All regulatory documentation including regulatory submissions, 1572 forms, and correspondence regarding this study will be kept by the Sponsor. The CRO that will conduct the study on behalf of the Sponsor will maintain all study documentation according to their SOPs.

9.6 Subject Identification Register

The investigator agrees to complete a subject identification register, which will be used for the purpose of long-term follow-up, if needed. This form will be treated as confidential, and will be filed by the investigator in a secure locked place. Otherwise, all reports and communications relating to the study will identify participants by initials and assigned number only.

9.7 Record Retention

The Investigator must retain all study records required by the Sponsor and by the applicable regulations in a secure and safe facility. The Investigator must consult a Sponsor CRA before disposal of any study records, and must notify the Sponsor of any change in the location, disposition, or custody of the study files. The FDA requires retention of records for two years following the date a marketing application is approved, or for two years after the FDA is notified that the IND is discontinued if there is no marketing application. Records must be retained for a period at least as long as that specified by FDA regulations.

9.8 Case Report Form Completion

Case Report Forms (CRFs) will be generated for each subject. If paper CRFs are used, correction to data on CRFs may be made only by putting a line through the incorrect data and writing the correct values, allowing the original text to remain legible. Each correction must be initialed and dated by the person making the change. If corrections are made after review and signature by the Investigator, s/he must be made aware of the changes and must document this awareness. If electronic CRFs are used, the electronic system must comply with CFR 21 Part 11.

It is the policy of the Sponsor that study data must be verifiable to the source data, which necessitates access to all original recordings, laboratory reports, and subjects' records. The Investigator must therefore agree to allow access to subjects' records, and source data must be made available for all study data. The subjects (or their legal representatives) must also allow access to the subjects' medical records, and they will be informed of this requirement and will indicate their agreement when giving Informed Consent.

9.9 Monitoring

At intervals during the study, as well as after the completion of subject enrollment, the study center will be monitored by a CRA for compliance, which will include ensuring that accurate and complete data are recorded on CRFs, and reviewing source documentation and drug accountability records. The study will be conducted according to the principles of GCP as accepted in the United States.

9.10 Data Quality Assurance

The study will be monitored by the study center's in-house Quality Assurance unit for compliance to the protocol, to GCP, to the study center's Standard Operating Procedures, and to Sponsor requirements.

9.11 On-site Audits

The Sponsor's representatives will meet with the study center prior to initiation of the study to review with the center personnel information regarding the investigational agent, protocol requirements, monitoring requirements, and reporting of serious adverse events.

In certain circumstances, a secondary audit may be conducted by members of a Quality Assurance group designated by the Sponsor. The Investigator will be informed if this is to take place and advised as to the nature of the audit. Representatives of the Food and Drug Administration (FDA) and/or representatives of other regulatory authorities may also conduct an audit of the study. If informed of such an audit, the Investigator should notify the Sponsor immediately.

9.12 Use of Information and Publication

It is understood by the Investigator that the information generated in this study will be used by the Sponsor in connection with the development of the product and therefore may be disclosed to government agencies in various countries. To allow for the use of information derived from the study, it is understood that the Investigator is obliged to provide the Sponsor with complete test results, all study data, and access to all study records.

The Sponsor recognizes the importance of communicating study data and may elect to publish some or all of the results of this study in scientific journals, at seminars or conferences, and/or in other manner(s) it so chooses. Results from this study shall not be made available to any third party by the investigating team without the express permission of the Sponsor.

10 REFERENCES

Abe K, Muyazaki M, Koji T, et al. 2001. Enhanced expression of complement C5a receptor mRNA in human diseased kidney assessed by in situ hybridization. Kidney Int 60:137-146.

Schreiber A, Xiao H, Jennette JC, et al. 2009. C5a Receptor mediates neutrophil activation and ANCA-induced glomerulonephritis. J Am Soc Nephrol 20:289-298.

11 APPENDICES

11.1 Statement of Obligations of Sponsor, Monitor, and Clinical Investigator

Sponsor and Monitor

If the Sponsor is not familiar with the Study Site, the Sponsor or its designated representative, will:

A. Conduct a prestudy visit to:

1. Establish the acceptability of the facility and record this in a written report.
2. Discuss the proposed clinical trial with the Investigator, review the CRF requirements, and supply the Investigator Brochure and the draft protocol for review and approval.
3. Discuss with the Investigator FDA and other regulatory requirements with respect to Informed Consent, IRB approval of the trial, the protocol, protocol amendments, and Informed Consent changes.

B. Conduct periodic site visits to:

1. Assure adherence to the protocol.
2. Review CRFs and medical records for accuracy and completeness of information.
3. Examine pharmacy records for documentation of quantity and date of receipt of investigational supplies, dispensation and accountability data for administration to each subject, loss of materials, contamination, and unused supplies.
4. Record and report observations on the progress of the trial and continued acceptability of the facilities in a Site Visit Report.
5. Review Investigator files for required documents, e.g., protocols, protocol amendments, IRB/EC approvals (protocols, amendments, Informed Consent, etc), IRB/EC charter and membership, and communications between the IRB/EC and the Investigator.

Clinical Investigator

A. IRB/EC

The Investigator must assure the monitor that the IRB/EC:

1. Meets FDA regulations as defined in 21 CFR Part 56 and other applicable requirements.
2. Has authority delegated by the parent institution and found in IRB by-laws, operation guidelines, or charter to approve or disapprove clinical trials and protocols, including Informed Consent Forms and other documents (protocol amendments, information to be supplied to subjects concerning Informed Consent, etc).

2. Complies with proper personnel makeup of an IRB/EC.

3. Convenes meetings using acceptable rules of order for making decisions, recording such decisions, and implementing them.

4. Files contain (a) documentation of its decisions such as are found in IRB/EC minutes and correspondence, (b) written guidelines or by-laws governing IRB/EC functions, (c) protocols, (d) protocol information to be supplied to the subject, (e) correspondence between the IRB/EC and the Investigator (Informed Consent Form changes, protocol amendments, etc).

B. Informed Consent of Human Subjects.

The Principal Investigator must assure the monitor that the Informed Consent Form:

1. Meets FDA regulations as defined in 21 CFR Part 50 Informed Consent, and other applicable requirements.

2. Has been approved by the IRB/EC, including, when required, information to be given to the subject regarding the trial in which s/he is enrolled.

   a. The Informed Consent Form includes the Basic Elements and any Additional Elements necessary.

   b. The subject and a study center representative sign the Informed Consent Form and the subject is given a copy.

C. Storage and Dispensing of Study Supplies.

The Investigator (or pharmacist or pharmacy technician) must demonstrate to the monitor that:

1. Adequate and accurate written records show receipt and disposition of all study supplies, including dates, serial or lot numbers, quantities received, and each quantity dispensed, administered, or used, with identification of each subject.

2. Purpose and reasons are given in written records for study material disposal, e.g., the amount contaminated, broken, or lost, and the quantity returned to the Sponsor.

D. Case Report Forms.

The Investigator must assure the monitor that:

1. Case report forms, when completed, accurately reflect the medical records on each subject.

2. Case report forms and medical records will be accessible to the monitor or FDA inspectors during site visits.

E. Files and Records.

The Investigator must assure the quality, integrity, and content of his or her files that will be inspected by the monitor and regulatory inspectors. The files must contain, at a minimum:

1. Correspondence between the IRB/EC and the Investigator.

2. The following documents:

    a. IRB/EC-approved protocols.

    b. IRB/EC-approved protocol amendments.

    c. IRB/EC-approved Informed Consent Form and information supplied to the subject.

    d. IRB/EC charter, membership, and qualifications.

3. Clinical supplies:

    a. Record of receipt, date and quantity, and batch or lot number.

    b. Disposition dates and quantity administered to each subject.

    c. Inventory records.

The FDA requires retention of records for two years following the date a marketing application is approved, or for two years after the FDA is notified that the IND is discontinued if there is no marketing application. Records must be retained for a period at least as long as that specified by FDA regulations.

11.2 Informed Consent Form

In seeking Informed Consent, the following information shall be provided to each subject:

(1) A statement that the study involves research, an explanation of the purposes of the research and the expected duration of the subject's participation, a description of the procedures to be followed, and identification of any procedures which are experimental.

(2) A description of any reasonably foreseeable risks or discomforts to the subject.

(3) A description of any benefits to the subject or to others that may reasonably be expected from the research.

(4) A disclosure of appropriate alternative procedures or courses of treatment, if any, that might be advantageous to the subject.

(5) A statement describing the extent, if any, to which confidentiality of records identifying the subject will be maintained and that notes the possibility that the Food and Drug Administration or other Regulatory agency may inspect the records.

(6) For research involving more than minimal risk, an explanation as to whether any compensation and as to whether any medical treatments are available if injury occurs and, if so, what they consist of, or where further information may be obtained.

(7) An explanation of whom to contact for answers to pertinent questions about the research and research subjects' rights, and whom to contact in the event of a research-related injury to the subject.

(8) A statement that participation is voluntary, that refusal to participate will involve no penalty or loss of benefits to which the subject is otherwise entitled, and that the subject may discontinue participation at any time without penalty or loss of benefits to which the subject is otherwise entitled.

ADDITIONAL ELEMENTS OF INFORMED CONSENT

(1) A statement that the particular treatment or procedure may involve risks to the subject (or to the embryo or fetus if the subject is or may become pregnant) which are currently unforeseeable.

(2) Anticipated circumstances under which the subject’s participation may be terminated by the Investigator without regard to the subject’s consent.

(3) Any additional costs to the subject that may result from participation in the research.

(4) The consequences of a subject’s decision to withdraw from the research and procedures for orderly termination of participation by the subject.

(5) A statement that significant new findings developed during the course of the research which may relate to the subject’s willingness to continue participation will be provided to the subject.

(6) The approximate number of subjects involved in the study.

11.3 Common Terminology Criteria for Adverse Events v3.0 (CTCAE)

The following 72 pages constitute Appendix 11.3.

Common Terminology Criteria for Adverse Events v3.0 (CTCAE)

Publish Date: August 9, 2006

Quick Reference

The NCI Common Terminology Criteria for Adverse Events v3.0 is a descriptive terminology which can be utilized for Adverse Event (AE) reporting. A grading (severity) scale is provided for each AE term.

Components and Organization

CATEGORY

A CATEGORY is a broad classification of AEs based on anatomy and/or pathophysiology. Within each CATEGORY, AEs are listed accompanied by their descriptions of severity (Grade).

Adverse Event Terms

An AE is any unfavorable and unintended sign (including an abnormal laboratory finding), symptom, or disease temporally associated with the use of a medical treatment or procedure that may or may *not* be considered related to the medical treatment or procedure. An AE is a term that is a unique representation of a specific event used for medical documentation and scientific analyses. Each AE term is mapped to a MedDRA term and code. AEs are listed alphabetically within CATEGORIES.

Short AE Name

The 'SHORT NAME' column is new and it is used to simplify documentation of AE names on Case Report Forms.

Supra-ordinate Terms

A supra-ordinate term is located within a CATEGORY and is a grouping term based on disease process, signs, symptoms, or diagnosis. A supra-ordinate term is followed by the word *'Select'* and is accompanied by specific AEs that are all related to the supra-ordinate term. Supra-ordinate terms provide clustering and consistent representation of Grade for related AEs. Supra-ordinate terms are not AEs, are not mapped to a MedDRA term and code, cannot be graded and cannot be used for reporting.

REMARK

A 'REMARK' is a clarification of an AE.

ALSO CONSIDER

An 'ALSO CONSIDER' indicates additional AEs that are to be graded if they are clinically significant.

NAVIGATION NOTE

A 'NAVIGATION NOTE' indicates the location of an AE term within the CTCAE document. It lists signs/symptoms alphabetically and the CTCAE term will appear in the same CATEGORY unless the 'NAVIGATION NOTE' states differently.

Grades

Grade refers to the severity of the AE. The CTCAE v3.0 displays Grades 1 through 5 with unique clinical descriptions of severity for each AE based on this general guideline:

Grade 1	Mild AE
Grade 2	Moderate AE
Grade 3	Severe AE
Grade 4	Life-threatening or disabling AE
Grade 5	Death related to AE

A Semi-colon indicates 'or' within the description of the grade.

An 'Em dash' (—) indicates a grade not available.

Not all Grades are appropriate for all AEs. Therefore, some AEs are listed with fewer than five options for Grade selection.

Grade 5

Grade 5 (Death) is not appropriate for some AEs and therefore is not an option.

The DEATH CATEGORY is new. Only one Supra-ordinate term is listed in this CATEGORY: 'Death not associated with CTCAE term – *Select*' with 4 AE options: Death NOS; Disease progression NOS; Multi-organ failure; Sudden death.

Important:

- Grade 5 is the only appropriate Grade
- This AE is to be used in the situation where a death
  1. cannot be reported using a CTCAE v3.0 term associated with Grade 5, or
  2. cannot be reported within a CTCAE CATEGORY as 'Other (Specify)'

Contents

ALLERGY/IMMUNOLOGY 1
AUDITORY/EAR 2
BLOOD/BONE MARROW 4
CARDIAC ARRHYTHMIA 5
CARDIAC GENERAL 7
COAGULATION 10
CONSTITUTIONAL SYMPTOMS 11
DEATH 13
DERMATOLOGY/SKIN 14
ENDOCRINE 17
GASTROINTESTINAL 19
GROWTH AND DEVELOPMENT 29
HEMORRHAGE/BLEEDING 30
HEPATOBILIARY/PANCREAS 34
INFECTION 35
LYMPHATICS 38
METABOLIC/LABORATORY 40
MUSCULOSKELETAL/SOFT TISSUE 43
NEUROLOGY 47
OCULAR/VISUAL 52
PAIN 55
PULMONARY/UPPER RESPIRATORY 56
RENAL/GENITOURINARY 60
SECONDARY MALIGNANCY 63
SEXUAL/REPRODUCTIVE FUNCTION 64
SURGERY/INTRA-OPERATIVE INJURY 66
SYNDROMES 68
VASCULAR 70

		Grade				
**Adverse Event**	**Short Name**	**1**	**2**	**3**	**4**	**5**
Allergic reaction/ hypersensitivity (including drug fever)	Allergic reaction	Transient flushing or rash; drug fever <38°C (<100.4°F)	Rash; flushing; urticaria; dyspnea; drug fever ≥38°C (≥100.4°F)	Symptomatic bronchospasm, with or without urticaria; parenteral medication(s) indicated; allergy-related edema/angioedema; hypotension	Anaphylaxis	Death
REMARK: Urticaria with manifestations of allergic or hypersensitivity reaction is graded as Allergic reaction/hypersensitivity (including drug fever).						
ALSO CONSIDER: Cytokine release syndrome/acute infusion reaction.						
Allergic rhinitis (including sneezing, nasal stuffiness, postnasal drip)	Rhinitis	Mild, intervention not indicated	Moderate, intervention indicated	—	—	—
REMARK: Rhinitis associated with obstruction or stenosis is graded as Obstruction/stenosis of airway – *Select* in the PULMONARY/UPPER RESPIRATORY CATEGORY.						
Autoimmune reaction	Autoimmune reaction	Asymptomatic and serologic or other evidence of autoimmune reaction, with normal organ function and intervention not indicated	Evidence of autoimmune reaction involving a non-essential organ or function (e.g., hypothyroidism)	Reversible autoimmune reaction involving function of a major organ or other adverse event (e.g., transient colitis or anemia)	Autoimmune reaction with life-threatening consequences	Death
ALSO CONSIDER: Colitis; Hemoglobin; Hemolysis (e.g., immune hemolytic anemia, drug-related hemolysis); Thyroid function, low (hypothyroidism).						
Serum sickness	Serum sickness	—	—	Present	—	Death
NAVIGATION NOTE: Splenic function is graded in the BLOOD/BONE MARROW CATEGORY.						
NAVIGATION NOTE: Urticaria as an isolated symptom is graded as Urticaria (hives, welts, wheals) in the DERMATOLOGY/SKIN CATEGORY.						
Vasculitis	Vasculitis	Mild, intervention not indicated	Symptomatic, non-steroidal medical intervention indicated	Steroids indicated	Ischemic changes; amputation indicated	Death
Allergy/Immunology – Other (Specify, __)	Allergy – Other (Specify)	Mild	Moderate	Severe	Life-threatening; disabling	Death

AUDITORY/EAR

		Grade				
**Adverse Event**	**Short Name**	**1**	**2**	**3**	**4**	**5**
NAVIGATION NOTE: Earache (otalgia) is graded as Pain – *Select* in the PAIN CATEGORY.						
Hearing: patients with/without baseline audiogram and enrolled in a monitoring program[1]	Hearing (monitoring program)	Threshold shift or loss of 15 – 25 dB relative to baseline, averaged at 2 or more contiguous test frequencies in at least one ear; or subjective change in the absence of a Grade 1 threshold shift	Threshold shift or loss of >25 – 90 dB, averaged at 2 contiguous test frequencies in at least one ear	Adult only: Threshold shift of >25 – 90 dB, averaged at 3 contiguous test frequencies in at least one ear<br>Pediatric: Hearing loss sufficient to indicate therapeutic intervention, including hearing aids (e.g., ≥20 dB bilateral HL in the speech frequencies; ≥30 dB unilateral HL; and requiring additional speech-language related services)	Adult only: Profound bilateral hearing loss (>90 dB)<br>Pediatric: Audiologic indication for cochlear implant and requiring additional speech-language related services	—
REMARK: Pediatric recommendations are identical to those for adults, unless specified. For children and adolescents (≤18 years of age) without a baseline test, pre-exposure/pre-treatment hearing should be considered to be <5 dB loss.						
Hearing: patients without baseline audiogram and not enrolled in a monitoring program[1]	Hearing (without monitoring program)	—	Hearing loss not requiring hearing aid or intervention (i.e., not interfering with ADL)	Hearing loss requiring hearing aid or intervention (i.e., interfering with ADL)	Profound bilateral hearing loss (>90 dB)	—
REMARK: Pediatric recommendations are identical to those for adults, unless specified. For children and adolescents (≤18 years of age) without a baseline test, pre-exposure/pre-treatment hearing should be considered to be <5 dB loss.						
Otitis, external ear (non-infectious)	Otitis, external	External otitis with erythema or dry desquamation	External otitis with moist desquamation, edema, enhanced cerumen or discharge; tympanic membrane perforation; tympanostomy	External otitis with mastoiditis; stenosis or osteomyelitis	Necrosis of soft tissue or bone	Death
ALSO CONSIDER: Hearing: patients with/without baseline audiogram and enrolled in a monitoring program[1]; Hearing: patients without baseline audiogram and not enrolled in a monitoring program[1].						
Otitis, middle ear (non-infectious)	Otitis, middle	Serous otitis	Serous otitis, medical intervention indicated	Otitis with discharge; mastoiditis	Necrosis of the canal soft tissue or bone	Death

AUDITORY/EAR

		Grade				
**Adverse Event**	**Short Name**	**1**	**2**	**3**	**4**	**5**
Tinnitus	Tinnitus	—	Tinnitus not interfering with ADL	Tinnitus interfering with ADL	Disabling	—
ALSO CONSIDER: Hearing: patients with/without baseline audiogram and enrolled in a monitoring program[1]; Hearing: patients without baseline audiogram and not enrolled in a monitoring program[1].						
Auditory/Ear – Other (Specify, __)	Auditory/Ear – Other (Specify)	Mild	Moderate	Severe	Life-threatening; disabling	Death

[1] Drug-induced ototoxicity should be distinguished from age-related threshold decrements or unrelated cochlear insult. When considering whether an adverse event has occurred, it is first necessary to classify the patient into one of two groups. (1) The patient is under standard treatment/enrolled in a clinical trial <2.5 years, and has a 15 dB or greater threshold shift averaged across two contiguous frequencies; or (2) The patient is under standard treatment/enrolled in a clinical trial >2.5 years, and the difference between the expected age-related and the observed threshold shifts is 15 dB or greater averaged across two contiguous frequencies. Consult standard references for appropriate age- and gender-specific hearing norms, e.g., Morrell, et al. Age- and gender-specific reference ranges for hearing level and longitudinal changes in hearing level. Journal of the Acoustical Society of America 100:1949-1967, 1996; or Shotland, et al. Recommendations for cancer prevention trials using potentially ototoxic test agents. Journal of Clinical Oncology 19:1658-1663, 2001.

In the absence of a baseline prior to initial treatment, subsequent audiograms should be referenced to an appropriate database of normals. ANSI. (1996)

American National Standard: Determination of occupational noise exposure and estimation of noise-induced hearing impairment, ANSI S 3.44-1996. (Standard S 3.44). New York: American National Standards Institute. The recommended ANSI S3.44 database is Annex B.

BLOOD/BONE MARROW

		Grade				
**Adverse Event**	**Short Name**	**1**	**2**	**3**	**4**	**5**
Bone marrow cellularity	Bone marrow cellularity	Mildly hypocellular or ≤25% reduction from normal cellularity for age	Moderately hypocellular or >25 – ≤50% reduction from normal cellularity for age	Severely hypocellular or >50 – ≤75% reduction cellularity from normal for age	—	Death
CD4 count	CD4 count	<LLN – 500/mm^3<br><LLN – 0.5 x 10^9 /L	<500 – 200/mm^3<br><0.5 – 0.2 x 10^9 /L	<200 – 50/mm^3<br><0.2 x 0.05 – 10^9 /L	<50/mm^3<br><0.05 x 10^9 /L	Death
Haptoglobin	Haptoglobin	<LLN	—	Absent	—	Death
Hemoglobin	Hemoglobin	<LLN – 10.0 g/dL<br><LLN – 6.2 mmol/L<br><LLN – 100 g/L	<10.0 – 8.0 g/dL<br><6.2 – 4.9 mmol/L<br><100 – 80g/L	<8.0 – 6.5 g/dL<br><4.9 – 4.0 mmol/L<br><80 – 65 g/L	<6.5 g/dL<br><4.0 mmol/L<br><65 g/L	Death
Hemolysis (e.g., immune hemolytic anemia, drug-related hemolysis)	Hemolysis	Laboratory evidence of hemolysis only (e.g., direct antiglobulin test [DAT, Coombs'] schistocytes)	Evidence of red cell destruction and ≥2 gm decrease in hemoglobin, no transfusion	Transfusion or medical intervention (e.g., steroids) indicated	Catastrophic consequences of hemolysis (e.g., renal failure, hypotension, bronchospasm, emergency splenectomy)	Death
ALSO CONSIDER: Haptoglobin; Hemoglobin.						
Iron overload	Iron overload	—	Asymptomatic iron overload, intervention not indicated	Iron overload, intervention indicated	Organ impairment (e.g., endocrinopathy, cardiopathy)	Death
Leukocytes (total WBC)	Leukocytes	<LLN – 3000/mm^3<br><LLN – 3.0 x 10^9 /L	<3000 – 2000/mm^3<br><3.0 – 2.0 x 10^9 /L	<2000 – 1000/mm^3<br><2.0 – 1.0 x 10^9 /L	<1000/mm^3<br><1.0 x 10^9 /L	Death
Lymphopenia	Lymphopenia	<LLN – 800/mm^3<br><LLN x 0.8 – 10^9 /L	<800 – 500/mm^3<br><0.8 – 0.5 x 10^9 /L	<500 – 200 mm^3<br><0.5 – 0.2 x 10^9 /L	<200/mm^3<br><0.2 x 10^9 /L	Death
Myelodysplasia	Myelodysplasia	—	—	Abnormal marrow cytogenetics (marrow blasts ≤5%)	RAEB or RAEB-T (marrow blasts >5%)	Death
Neutrophils/granulocytes (ANC/AGC)	Neutrophils	<LLN – 1500/mm^3<br><LLN – 1.5 x 10^9 /L	<1500 – 1000/mm^3<br><1.5 – 1.0 x 10^9 /L	<1000 – 500/mm^3<br><1.0 – 0.5 x 10^9 /L	<500/mm^3<br><0.5 x 10^9 /L	Death
Platelets	Platelets	<LLN – 75,000/mm^3<br><LLN – 75.0 x 10^9 /L	<75,000 – 50,000/mm^3<br><75.0 – 50.0 x 10^9 /L	<50,000 – 25,000/mm^3<br><50.0 – 25.0 x 10^9 /L	<25,000/mm^3<br><25.0 x 10^9 /L	Death
Splenic function	Splenic function	Incidental findings (e.g., Howell-Jolly bodies)	Prophylactic antibiotics indicated	—	Life-threatening consequences	Death
Blood/Bone Marrow – Other (Specify, __)	Blood – Other (Specify)	Mild	Moderate	Severe	Life-threatening; disabling	Death

CARDIAC ARRHYTHMIA

		Grade				
**Adverse Event**	**Short Name**	**1**	**2**	**3**	**4**	**5**
Conduction abnormality/ atrioventricular heart block – *Select*:<br>– Asystole<br>– AV Block-First degree<br>– AV Block-Second degree Mobitz Type I (Wenckebach)<br>– AV Block-Second degree Mobitz Type II<br>– AV Block-Third degree (Complete AV block)<br>– Conduction abnormality NOS<br>– Sick Sinus Syndrome<br>– Stokes-Adams Syndrome<br>– Wolff-Parkinson-White Syndrome	Conduction abnormality – *Select*	Asymptomatic, intervention not indicated	Non-urgent medical intervention indicated	Incompletely controlled medically or controlled with device (e.g., pacemaker)	Life-threatening (e.g., arrhythmia associated with CHF, hypotension, syncope, shock)	Death
Palpitations	Palpitations	Present	Present with associated symptoms (e.g., lightheadedness, shortness of breath)	—	—	—
REMARK: Grade palpitations <u>only</u> in the absence of a documented arrhythmia.						
Prolonged QTc interval	Prolonged QTc	QTc >0.45 – 0.47 second	QTc >0.47 – 0.50 second; ≥0.06 second above baseline	QTc >0.50 second	QTc >0.50 second; life-threatening signs or symptoms (e.g., arrhythmia, CHF, hypotension, shock syncope); Torsade de pointes	Death
Supraventricular and nodal arrhythmia – *Select*:<br>– Atrial fibrillation<br>– Atrial flutter<br>– Atrial tachycardia/Paroxysmal Atrial Tachycardia<br>– Nodal/Junctional<br>– Sinus arrhythmia<br>– Sinus bradycardia<br>– Sinus tachycardia<br>– Supraventricular arrhythmia NOS<br>– Supraventricular extrasystoles (Premature Atrial Contractions; Premature Nodal/Junctional Contractions)<br>– Supraventricular tachycardia	Supraventricular arrhythmia – *Select*	Asymptomatic, intervention not indicated	Non-urgent medical intervention indicated	Symptomatic and incompletely controlled medically, or controlled with device (e.g., pacemaker)	Life-threatening (e.g., arrhythmia associated with CHF, hypotension, syncope, shock)	Death
NAVIGATION NOTE: Syncope is graded as Syncope (fainting) in the NEUROLOGY CATEGORY.						

CARDIAC ARRHYTHMIA

		Grade				
**Adverse Event**	**Short Name**	**1**	**2**	**3**	**4**	**5**
Vasovagal episode	Vasovagal episode	—	Present without loss of consciousness	Present with loss of consciousness	Life-threatening consequences	Death
Ventricular arrhythmia – *Select:*<br>– Bigeminy<br>– Idioventricular rhythm<br>– PVCs<br>– Torsade de pointes<br>– Trigeminy<br>– Ventricular arrhythmia NOS<br>– Ventricular fibrillation<br>– Ventricular flutter<br>– Ventricular tachycardia	Ventricular arrhythmia – *Select*	Asymptomatic, no intervention indicated	Non-urgent medical intervention indicated	Symptomatic and incompletely controlled medically or controlled with device (e.g., defibrillator)	Life-threatening (e.g., arrhythmia associated with CHF, hypotension, syncope, shock)	Death
Cardiac Arrhythmia – Other (Specify, __)	Cardiac Arrhythmia – Other (Specify)	Mild	Moderate	Severe	Life-threatening; disabling	Death

CARDIAC GENERAL

		Grade				
**Adverse Event**	**Short Name**	**1**	**2**	**3**	**4**	**5**
NAVIGATION NOTE: Angina is graded as Cardiac ischemia/infarction in the CARDIAC GENERAL CATEGORY.						
Cardiac ischemia/infarction	Cardiac ischemia/infarction	Asymptomatic arterial narrowing without ischemia	Asymptomatic and testing suggesting ischemia; stable angina	Symptomatic and testing consistent with ischemia; unstable angina; intervention indicated	Acute myocardial infarction	Death
Cardiac troponin I (cTnI)	cTnI	—	—	Levels consistent with unstable angina as defined by the manufacturer	Levels consistent with myocardial infarction as defined by the manufacturer	Death
Cardiac troponin T (cTnT)	cTnT	0.03 – <0.05 ng/mL	0.05 – <0.1 ng/mL	0.1 – <0.2 ng/mL	0.2 ng/mL	Death
Cardiopulmonary arrest, cause unknown (non-fatal)	Cardiopulmonary arrest	—	—	—	Life-threatening	—
REMARK: Grade 4 (non-fatal) is the only appropriate grade. CTCAE provides three alternatives for reporting Death:<br>1. A CTCAE term associated with Grade 5.<br>2. A CTCAE 'Other (Specify, __)' within any CATEGORY.<br>3. Death not associated with CTCAE term – *Select* in the DEATH CATEGORY.						
NAVIGATION NOTE: Chest pain (non-cardiac and non-pleuritic) is graded as Pain – *Select* in the PAIN CATEGORY.						
NAVIGATION NOTE: CNS ischemia is graded as CNS cerebrovascular ischemia in the NEUROLOGY CATEGORY.						
Hypertension	Hypertension	Asymptomatic, transient (<24 hrs) increase by >20 mmHg (diastolic) or to >150/100 if previously WNL; intervention not indicated<br><br>Pediatric:<br>Asymptomatic, transient (<24 hrs) BP increase >ULN; intervention not indicated	Recurrent or persistent (≥24 hrs) or symptomatic increase by >20 mmHg (diastolic) or to >150/100 if previously WNL; monotherapy may be indicated<br><br>Pediatric:<br>Recurrent or persistent (≥24 hrs) BP >ULN; monotherapy may be indicated	Requiring more than one drug or more intensive therapy than previously<br><br>Pediatric:<br>Same as adult	Life-threatening consequences (e.g., hypertensive crisis)<br><br>Pediatric:<br>Same as adult	Death
REMARK: Use age and gender-appropriate normal values >95th percentile ULN for pediatric patients.						

CARDIAC GENERAL

		Grade				
**Adverse Event**	**Short Name**	**1**	**2**	**3**	**4**	**5**
Hypotension	Hypotension	Changes, intervention not indicated	Brief (<24 hrs) fluid replacement or other therapy; no physiologic consequences	Sustained (≥24 hrs) therapy, resolves without persisting physiologic consequences	Shock (e.g., acidemia; impairment of vital organ function)	Death
ALSO CONSIDER: Syncope (fainting).						
Left ventricular diastolic dysfunction	Left ventricular diastolic dysfunction	Asymptomatic diagnostic finding; intervention not indicated	Asymptomatic, intervention indicated	Symptomatic CHF responsive to intervention	Refractory CHF, poorly controlled; intervention such as ventricular assist device or heart transplant indicated	Death
Left ventricular systolic dysfunction	Left ventricular systolic dysfunction	Asymptomatic, resting ejection fraction (EF) <60 – 50%; shortening fraction (SF) <30 – 24%	Asymptomatic, resting EF <50 – 40%; SF <24 – 15%	Symptomatic CHF responsive to intervention; EF <40 – 20% SF <15%	Refractory CHF or poorly controlled; EF <20%; intervention such as ventricular assist device, ventricular reduction surgery, or heart transplant indicated	Death
NAVIGATION NOTE: Myocardial infarction is graded as Cardiac ischemia/infarction in the CARDIAC GENERAL CATEGORY.						
Myocarditis	Myocarditis	—	—	CHF responsive to intervention	Severe or refractory CHF	Death
Pericardial effusion (non-malignant)	Pericardial effusion	Asymptomatic effusion	—	Effusion with physiologic consequences	Life-threatening consequences (e.g., tamponade); emergency intervention indicated	Death
Pericarditis	Pericarditis	Asymptomatic, ECG or physical exam (rub) changes consistent with pericarditis	Symptomatic pericarditis (e.g., chest pain)	Pericarditis with physiologic consequences (e.g., pericardial constriction)	Life-threatening consequences; emergency intervention indicated	Death
NAVIGATION NOTE: Pleuritic pain is graded as Pain – *Select* in the PAIN CATEGORY.						
Pulmonary hypertension	Pulmonary hypertension	Asymptomatic without therapy	Asymptomatic, therapy indicated	Symptomatic hypertension, responsive to therapy	Symptomatic hypertension, poorly controlled	Death
Restrictive cardiomyopathy	Restrictive cardiomyopathy	Asymptomatic, therapy not indicated	Asymptomatic, therapy indicated	Symptomatic CHF responsive to intervention	Refractory CHF, poorly controlled; intervention such as ventricular assist device, or heart transplant indicated	Death

		Grade				
**Adverse Event**	**Short Name**	**1**	**2**	**3**	**4**	**5**
Right ventricular dysfunction (cor pulmonale)	Right ventricular dysfunction	Asymptomatic without therapy	Asymptomatic, therapy indicated	Symptomatic cor pulmonale, responsive to intervention	Symptomatic cor pulmonale poorly controlled; intervention such as ventricular assist device, or heart transplant indicated	Death
Valvular heart disease	Valvular heart disease	Asymptomatic valvular thickening with or without mild valvular regurgitation or stenosis; treatment other than endocarditis prophylaxis not indicated	Asymptomatic; moderate regurgitation or stenosis by imaging	Symptomatic; severe regurgitation or stenosis; symptoms controlled with medical therapy	Life-threatening; disabling; intervention (e.g., valve replacement, valvuloplasty) indicated	Death
Cardiac General – Other (Specify, __)	Cardiac General – Other (Specify)	Mild	Moderate	Severe	Life-threatening; disabling	Death

COAGULATION

		Grade				
**Adverse Event**	**Short Name**	**1**	**2**	**3**	**4**	**5**
DIC (disseminated intravascular coagulation)	DIC	—	Laboratory findings with no bleeding	Laboratory findings and bleeding	Laboratory findings, life-threatening or disabling consequences (e.g., CNS hemorrhage, organ damage, or hemodynamically significant blood loss)	Death
REMARK: DIC (disseminated intravascular coagulation) must have increased fibrin split products or D-dimer.						
ALSO CONSIDER: Platelets.						
Fibrinogen	Fibrinogen	<1.0 – 0.75 x LLN or <25% decrease from baseline	<0.75 – 0.5 x LLN or 25 – <50% decrease from baseline	<0.5 – 0.25 x LLN or 50 – <75% decrease from baseline	<0.25 x LLN or 75% decrease from baseline or absolute value <50 mg/dL	Death
REMARK: Use % decrease only when baseline is <LLN (local laboratory value).						
INR (International Normalized Ratio of prothrombin time)	INR	>1 – 1.5 x ULN	>1.5 – 2 x ULN	>2 x ULN	—	—
ALSO CONSIDER: Hemorrhage, CNS; Hemorrhage, GI – *Select*; Hemorrhage, GU – *Select*; Hemorrhage, pulmonary/upper respiratory – *Select*.						
PTT (Partial Thromboplastin Time)	PTT	>1 – 1.5 x ULN	>1.5 – 2 x ULN	>2 x ULN	—	—
ALSO CONSIDER: Hemorrhage, CNS; Hemorrhage, GI – *Select*; Hemorrhage, GU – *Select*; Hemorrhage, pulmonary/upper respiratory – *Select*.						
Thrombotic microangiopathy (e.g., thrombotic thrombocytopenic purpura [TTP] or hemolytic uremic syndrome [HUS])	Thrombotic microangiopathy	Evidence of RBC destruction (schistocytosis) without clinical consequences	—	Laboratory findings present with clinical consequences (e.g., renal insufficiency, petechiae)	Laboratory findings and life-threatening or disabling consequences, (e.g., CNS hemorrhage/ bleeding or thrombosis/ embolism or renal failure)	Death
REMARK: Must have microangiopathic changes on blood smear (e.g., schistocytes, helmet cells, red cell fragments).						
ALSO CONSIDER: Creatinine; Hemoglobin; Platelets.						
Coagulation – Other (Specify, __)	Coagulation – Other (Specify)	Mild	Moderate	Severe	Life-threatening; disabling	Death

CONSTITUTIONAL SYMPTOMS

		Grade				
**Adverse Event**	**Short Name**	**1**	**2**	**3**	**4**	**5**
Fatigue (asthenia, lethargy, malaise)	Fatigue	Mild fatigue over baseline	Moderate or causing difficulty performing some ADL	Severe fatigue interfering with ADL	Disabling	—
Fever (in the absence of neutropenia, where neutropenia is defined as ANC <1.0 x 10^9/L)	Fever	38.0 – 39.0°C (100.4 – 102.2°F)	>39.0 – 40.0°C (102.3 – 104.0°F)	>40.0°C (>104.0°F) for ≤24 hrs	>40.0°C (>104.0°F) for >24 hrs	Death
REMARK: The temperature measurements listed are oral or tympanic.						
ALSO CONSIDER: Allergic reaction/hypersensitivity (including drug fever).						
NAVIGATION NOTE: Hot flashes are graded as Hot flashes/flushes in the ENDOCRINE CATEGORY.						
Hypothermia	Hypothermia	—	35 – >32°C<br>95 – >89.6°F	32 – >28°C<br>89.6 – >82.4° F	≤28 °C<br>82.4°F or life-threatening consequences (e.g., coma, hypotension, pulmonary edema, acidemia, ventricular fibrillation)	Death
Insomnia	Insomnia	Occasional difficulty sleeping, not interfering with function	Difficulty sleeping, interfering with function but not interfering with ADL	Frequent difficulty sleeping, interfering with ADL	Disabling	—
REMARK: If pain or other symptoms interfere with sleep, do NOT grade as insomnia. Grade primary event(s) causing insomnia.						
Obesity[2]	Obesity	—	BMI 25 – 29.9 kg/m^2	BMI 30 – 39.99 kg/m^2	BMI ≥40 kg/m^2	—
REMARK: BMI = (weight [kg]) / (height [m])^2						
Odor (patient odor)	Patient odor	Mild odor	Pronounced odor	—	—	—
Rigors/chills	Rigors/chills	Mild	Moderate, narcotics indicated	Severe or prolonged, not responsive to narcotics	—	—

[2] NHLBI Obesity Task Force. "Clinical Guidelines on the Identification, Evaluation, and Treatment of Overweight and Obesity in Adults," *The Evidence Report,* Obes Res 6:51S-209S, 1998.

CONSTITUTIONAL SYMPTOMS

		Grade				
**Adverse Event**	**Short Name**	**1**	**2**	**3**	**4**	**5**
Sweating (diaphoresis)	Sweating	Mild and occasional	Frequent or drenching	—	—	—
ALSO CONSIDER: Hot flashes/flushes.						
Weight gain	Weight gain	5 – <10% of baseline	10 – <20% of baseline	≥20% of baseline	—	—
REMARK: Edema, depending on etiology, is graded in the CARDIAC GENERAL or LYMPHATICS CATEGORIES.						
ALSO CONSIDER: Ascites (non-malignant); Pleural effusion (non-malignant).						
Weight loss	Weight loss	5 to <10% from baseline; intervention not indicated	10 – <20% from baseline; nutritional support indicated	≥20% from baseline; tube feeding or TPN indicated	—	—
Constitutional Symptoms – Other (Specify, __)	Constitutional Symptoms – Other (Specify)	Mild	Moderate	Severe	Life-threatening; disabling	Death

DEATH

		Grade				
**Adverse Event**	**Short Name**	**1**	**2**	**3**	**4**	**5**
Death not associated with CTCAE term – *Select*:<br>– Death NOS<br>– Disease progression NOS<br>– Multi-organ failure<br>– Sudden death	Death not associated with CTCAE term – *Select*	—	—	—	—	Death

REMARK: Grade 5 is the only appropriate grade. 'Death not associated with CTCAE term – *Select'* is to be used where a death:

1. Cannot be attributed to a CTCAE term associated with Grade 5.
2. Cannot be reported within any CATEGORY using a CTCAE 'Other (Specify, __)'.

DERMATOLOGY/SKIN						Page 1 of 3
		Grade				
**Adverse Event**	**Short Name**	**1**	**2**	**3**	**4**	**5**
Atrophy, skin	Atrophy, skin	Detectable	Marked	—	—	—
Atrophy, subcutaneous fat	Atrophy, subcutaneous fat	Detectable	Marked	—	—	—
ALSO CONSIDER: Induration/fibrosis (skin and subcutaneous tissue).						
Bruising (in absence of Grade 3 or 4 thrombocytopenia)	Bruising	Localized or in a dependent area	Generalized	—	—	—
Burn	Burn	Minimal symptoms; intervention not indicated	Medical intervention; minimal debridement indicated	Moderate to major debridement or reconstruction indicated	Life-threatening consequences	Death
REMARK: Burn refers to all burns including radiation, chemical, etc.						
Cheilitis	Cheilitis	Asymptomatic	Symptomatic, not interfering with ADL	Symptomatic, interfering with ADL	—	—
Dry skin	Dry skin	Asymptomatic	Symptomatic, not interfering with ADL	Interfering with ADL	—	—
Flushing	Flushing	Asymptomatic	Symptomatic	—	—	—
Hair loss/alopecia (scalp or body)	Alopecia	Thinning or patchy	Complete	—	—	—
Hyperpigmentation	Hyperpigmentation	Slight or localized	Marked or generalized	—	—	—
Hypopigmentation	Hypopigmentation	Slight or localized	Marked or generalized	—	—	—
Induration/fibrosis (skin and subcutaneous tissue)	Induration	Increased density on palpation	Moderate impairment of function not interfering with ADL; marked increase in density and firmness on palpation with or without minimal retraction	Dysfunction interfering with ADL; very marked density, retraction or fixation	—	—
ALSO CONSIDER: Fibrosis-cosmesis; Fibrosis-deep connective tissue.						
Injection site reaction/ extravasation changes	Injection site reaction	Pain; itching; erythema	Pain or swelling, with inflammation or phlebitis	Ulceration or necrosis that is severe; operative intervention indicated	—	—
ALSO CONSIDER: Allergic reaction/hypersensitivity (including drug fever); Ulceration.						

DERMATOLOGY/SKIN

		Grade				
**Adverse Event**	**Short Name**	**1**	**2**	**3**	**4**	**5**
Nail changes	Nail changes	Discoloration; ridging (koilonychias); pitting	Partial or complete loss of nail(s); pain in nailbed(s)	Interfering with ADL	—	—
NAVIGATION NOTE: Petechiae is graded as Petechiae/purpura (hemorrhage/bleeding into skin or mucosa) in the HEMORRHAGE/BLEEDING CATEGORY.						
Photosensitivity	Photosensitivity	Painless erythema	Painful erythema	Erythema with desquamation	Life-threatening; disabling	Death
Pruritus/itching	Pruritus	Mild or localized	Intense or widespread	Intense or widespread and interfering with ADL	—	—
ALSO CONSIDER: Rash/desquamation.						
Rash/desquamation	Rash	Macular or papular eruption or erythema without associated symptoms	Macular or papular eruption or erythema with pruritus or other associated symptoms; localized desquamation or other lesions covering <50% of body surface area (BSA)	Severe, generalized erythroderma or macular, papular or vesicular eruption; desquamation covering ≥50% BSA	Generalized exfoliative, ulcerative, or bullous dermatitis	Death
REMARK: Rash/desquamation may be used for GVHD.						
Rash: acne/acneiform	Acne	Intervention not indicated	Intervention indicated	Associated with pain, disfigurement, ulceration, or desquamation	—	Death
Rash: dermatitis associated with radiation – *Select*: – Chemoradiation – Radiation	Dermatitis – *Select*	Faint erythema or dry desquamation	Moderate to brisk erythema; patchy moist desquamation, mostly confined to skin folds and creases; moderate edema	Moist desquamation other than skin folds and creases; bleeding induced by minor trauma or abrasion	Skin necrosis or ulceration of full thickness dermis; spontaneous bleeding from involved site	Death
Rash: erythema multiforme (e.g., Stevens-Johnson syndrome, toxic epidermal necrolysis)	Erythema multiforme	—	Scattered, but not generalized eruption	Severe (e.g., generalized rash or painful stomatitis); IV fluids, tube feedings, or TPN indicated	Life-threatening; disabling	Death
Rash: hand-foot skin reaction	Hand-foot	Minimal skin changes or dermatitis (e.g., erythema) without pain	Skin changes (e.g., peeling, blisters, bleeding, edema) or pain, not interfering with function	Ulcerative dermatitis or skin changes with pain interfering with function	—	—

DERMATOLOGY/SKIN

		Grade				
**Adverse Event**	**Short Name**	**1**	**2**	**3**	**4**	**5**
Skin breakdown/ decubitus ulcer	Decubitus	—	Local wound care; medical intervention indicated	Operative debridement or other invasive intervention indicated (e.g., hyperbaric oxygen)	Life-threatening consequences; major invasive intervention indicated (e.g., tissue reconstruction, flap, or grafting)	Death
REMARK: Skin breakdown/decubitus ulcer is to be used for loss of skin integrity or decubitus ulcer from pressure or as the result of operative or medical intervention.						
Striae	Striae	Mild	Cosmetically significant	—	—	—
Telangiectasia	Telangiectasia	Few	Moderate number	Many and confluent	—	—
Ulceration	Ulceration	—	Superficial ulceration <2 cm size; local wound care; medical intervention indicated	Ulceration ≥2 cm size; operative debridement, primary closure or other invasive intervention indicated (e.g., hyperbaric oxygen)	Life-threatening consequences; major invasive intervention indicated (e.g., complete resection, tissue reconstruction, flap, or grafting)	Death
Urticaria (hives, welts, wheals)	Urticaria	Intervention not indicated	Intervention indicated for <24 hrs	Intervention indicated for ≥24 hrs	—	—
ALSO CONSIDER: Allergic reaction/hypersensitivity (including drug fever).						
Wound complication, non-infectious	Wound complication, non-infectious	Incisional separation of ≤25% of wound, no deeper than superficial fascia	Incisional separation >25% of wound with local care; asymptomatic hernia	Symptomatic hernia without evidence of strangulation; fascial disruption/dehiscence without evisceration; primary wound closure or revision by operative intervention indicated; hospitalization or hyperbaric oxygen indicated	Symptomatic hernia with evidence of strangulation; fascial disruption with evisceration; major reconstruction flap, grafting, resection, or amputation indicated	Death
REMARK: Wound complication, non-infectious is to be used for separation of incision, hernia, dehiscence, evisceration, or second surgery for wound revision.						
Dermatology/Skin – Other (Specify, __)	Dermatology – Other (Specify)	Mild	Moderate	Severe	Life-threatening; disabling	Death

ENDOCRINE

		Grade				
**Adverse Event**	**Short Name**	**1**	**2**	**3**	**4**	**5**
Adrenal insufficiency	Adrenal insufficiency	Asymptomatic, intervention not indicated	Symptomatic, intervention indicated	Hospitalization	Life-threatening; disabling	Death
REMARK: Adrenal insufficiency includes any of the following signs and symptoms: abdominal pain, anorexia, constipation, diarrhea, hypotension, pigmentation of mucous membranes, pigmentation of skin, salt craving, syncope (fainting), vitiligo, vomiting, weakness, weight loss. Adrenal insufficiency must be confirmed by laboratory studies (low cortisol frequently accompanied by low aldosterone). ALSO CONSIDER: Potassium, serum-high (hyperkalemia); Thyroid function, low (hypothyroidism).						
Cushingoid appearance (e.g., moon face, buffalo hump, centripetal obesity, cutaneous striae)	Cushingoid	—	Present	—	—	—
ALSO CONSIDER: Glucose, serum-high (hyperglycemia); Potassium, serum-low (hypokalemia).						
Feminization of male	Feminization of male	—	—	Present	—	—
NAVIGATION NOTE: Gynecomastia is graded in the SEXUAL/REPRODUCTIVE FUNCTION CATEGORY.						
Hot flashes/flushes[3]	Hot flashes	Mild	Moderate	Interfering with ADL	—	—
Masculinization of female	Masculinization of female	—	—	Present	—	—
Neuroendocrine: ACTH deficiency	ACTH	Asymptomatic	Symptomatic, not interfering with ADL; intervention indicated	Symptoms interfering with ADL; hospitalization indicated	Life-threatening consequences (e.g., severe hypotension)	Death
Neuroendocrine: ADH secretion abnormality (e.g., SIADH or low ADH)	ADH	Asymptomatic	Symptomatic, not interfering with ADL; intervention indicated	Symptoms interfering with ADL	Life-threatening consequences	Death
Neuroendocrine: gonadotropin secretion abnormality	Gonadotropin	Asymptomatic	Symptomatic, not interfering with ADL; intervention indicated	Symptoms interfering with ADL; osteopenia; fracture; infertility	—	—
Neuroendocrine: growth hormone secretion abnormality	Growth hormone	Asymptomatic	Symptomatic, not interfering with ADL; intervention indicated	—	—	—
Neuroendocrine: prolactin hormone secretion abnormality	Prolactin	Asymptomatic	Symptomatic, not interfering with ADL; intervention indicated	Symptoms interfering with ADL; amenorrhea; galactorrhea	—	Death

[3] Sloan JA, Loprinzi CL, Novotny PJ, Barton DL, Lavasseur BI, Windschitl HJ, "Methodologic Lessons Learned from Hot Flash Studies," *J Clin Oncol* 2001 Dec 1;19(23):4280-90

ENDOCRINE

		Grade				
**Adverse Event**	**Short Name**	**1**	**2**	**3**	**4**	**5**
Pancreatic endocrine: glucose intolerance	Diabetes	Asymptomatic, intervention not indicated	Symptomatic; dietary modification or oral agent indicated	Symptoms interfering with ADL; insulin indicated	Life-threatening consequences (e.g., ketoacidosis, hyperosmolar non-ketotic coma)	Death
Parathyroid function, low (hypoparathyroidism)	Hypoparathyroidism	Asymptomatic, intervention not indicated	Symptomatic; intervention indicated	—	—	—
Thyroid function, high (hyperthyroidism, thyrotoxicosis)	Hyperthyroidism	Asymptomatic, intervention not indicated	Symptomatic, not interfering with ADL; thyroid suppression therapy indicated	Symptoms interfering with ADL; hospitalization indicated	Life-threatening consequences (e.g., thyroid storm)	Death
Thyroid function, low (hypothyroidism)	Hypothyroidism	Asymptomatic, intervention not indicated	Symptomatic, not interfering with ADL; thyroid replacement indicated	Symptoms interfering with ADL; hospitalization indicated	Life-threatening myxedema coma	Death
Endocrine – Other (Specify, __)	Endocrine – Other (Specify)	Mild	Moderate	Severe	Life-threatening; disabling	Death

		Grade				
**Adverse Event**	**Short Name**	**1**	**2**	**3**	**4**	**5**
NAVIGATION NOTE: Abdominal pain or cramping is graded as Pain – *Select* in the PAIN CATEGORY.						
Anorexia	Anorexia	Loss of appetite without alteration in eating habits	Oral intake altered without significant weight loss or malnutrition; oral nutritional supplements indicated	Associated with significant weight loss or malnutrition (e.g., inadequate oral caloric and/or fluid intake); IV fluids, tube feedings or TPN indicated	Life-threatening consequences	Death
ALSO CONSIDER: Weight loss.						
Ascites (non-malignant)	Ascites	Asymptomatic	Symptomatic, medical intervention indicated	Symptomatic, invasive procedure indicated	Life-threatening consequences	Death
REMARK: Ascites (non-malignant) refers to documented non-malignant ascites or unknown etiology, but unlikely malignant, and includes chylous ascites.						
Colitis	Colitis	Asymptomatic, pathologic or radiographic findings only	Abdominal pain; mucus or blood in stool	Abdominal pain, fever, change in bowel habits with ileus; peritoneal signs	Life-threatening consequences (e.g., perforation, bleeding, ischemia, necrosis, toxic megacolon)	Death
ALSO CONSIDER: Hemorrhage, GI – *Select*.						
Constipation	Constipation	Occasional or intermittent symptoms; occasional use of stool softeners, laxatives, dietary modification, or enema	Persistent symptoms with regular use of laxatives or enemas indicated	Symptoms interfering with ADL; obstipation with manual evacuation indicated	Life-threatening consequences (e.g., obstruction, toxic megacolon)	Death
ALSO CONSIDER: Ileus, GI (functional obstruction of bowel, i.e., neuroconstipation); Obstruction, GI – *Select*.						
Dehydration	Dehydration	Increased oral fluids indicated; dry mucous membranes; diminished skin turgor	IV fluids indicated <24 hrs	IV fluids indicated ≥24 hrs	Life-threatening consequences (e.g., hemodynamic collapse)	Death
ALSO CONSIDER: Diarrhea; Hypotension; Vomiting.						
Dental: dentures or prosthesis	Dentures	Minimal discomfort, no restriction in activities	Discomfort preventing use in some activities (e.g., eating), but not others (e.g., speaking)	Unable to use dentures or prosthesis at any time	—	—

GASTROINTESTINAL

		Grade				
**Adverse Event**	**Short Name**	**1**	**2**	**3**	**4**	**5**
Dental:<br>periodontal disease	Periodontal	Gingival recession or gingivitis; limited bleeding on probing; mild local bone loss	Moderate gingival recession or gingivitis; multiple sites of bleeding on probing; moderate bone loss	Spontaneous bleeding; severe bone loss with or without tooth loss; osteonecrosis of maxilla or mandible	—	—
REMARK: Severe periodontal disease leading to osteonecrosis is graded as Osteonecrosis (avascular necrosis) in the MUSCULOSKELETAL CATEGORY.						
Dental:<br>teeth	Teeth	Surface stains; dental caries; restorable, without extractions	Less than full mouth extractions; tooth fracture or crown amputation or repair indicated	Full mouth extractions indicated	—	—
Dental:<br>teeth development	Teeth development	Hypoplasia of tooth or enamel not interfering with function	Functional impairment correctable with oral surgery	Maldevelopment with functional impairment not surgically correctable	—	—
Diarrhea	Diarrhea	Increase of <4 stools per day over baseline; mild increase in ostomy output compared to baseline	Increase of 4 – 6 stools per day over baseline; IV fluids indicated <24hrs; moderate increase in ostomy output compared to baseline; not interfering with ADL	Increase of ≥7 stools per day over baseline; incontinence; IV fluids ≥24 hrs; hospitalization; severe increase in ostomy output compared to baseline; interfering with ADL	Life-threatening consequences (e.g., hemodynamic collapse)	Death
REMARK: Diarrhea includes diarrhea of small bowel or colonic origin, and/or ostomy diarrhea.<br>ALSO CONSIDER: Dehydration; Hypotension.						
Distension/bloating, abdominal	Distension	Asymptomatic	Symptomatic, but not interfering with GI function	Symptomatic, interfering with GI function	—	—
ALSO CONSIDER: Ascites (non-malignant); Ileus, GI (functional obstruction of bowel, i.e., neuroconstipation); Obstruction, GI – *Select*.						

Adverse Event	Short Name	Grade 1	Grade 2	Grade 3	Grade 4	Grade 5
Dry mouth/salivary gland (xerostomia)	Dry mouth	Symptomatic (dry or thick saliva) without significant dietary alteration; unstimulated saliva flow >0.2 ml/min	Symptomatic and significant oral intake alteration (e.g., copious water, other lubricants, diet limited to purees and/or soft, moist foods); unstimulated saliva 0.1 to 0.2 ml/min	Symptoms leading to inability to adequately aliment orally; IV fluids, tube feedings, or TPN indicated; unstimulated saliva <0.1 ml/min	—	—
REMARK: Dry mouth/salivary gland (xerostomia) includes descriptions of grade using both subjective and objective assessment parameters. Record this event consistently throughout a patient's participation on study. If salivary flow measurements are used for initial assessment, subsequent assessments must use salivary flow.						
ALSO CONSIDER: Salivary gland changes/saliva.						
Dysphagia (difficulty swallowing)	Dysphagia	Symptomatic, able to eat regular diet	Symptomatic and altered eating/swallowing (e.g., altered dietary habits, oral supplements); IV fluids indicated <24 hrs	Symptomatic and severely altered eating/swallowing (e.g., inadequate oral caloric or fluid intake); IV fluids, tube feedings, or TPN indicated ≥24 hrs	Life-threatening consequences (e.g., obstruction, perforation)	Death
REMARK: Dysphagia (difficulty swallowing) is to be used for swallowing difficulty from oral, pharyngeal, esophageal, or neurologic origin. Dysphagia requiring dilation is graded as Stricture/stenosis (including anastomotic), GI – *Select*.						
ALSO CONSIDER: Dehydration; Esophagitis.						
Enteritis (inflammation of the small bowel)	Enteritis	Asymptomatic, pathologic or radiographic findings only	Abdominal pain; mucus or blood in stool	Abdominal pain, fever, change in bowel habits with ileus; peritoneal signs	Life-threatening consequences (e.g., perforation, bleeding, ischemia, necrosis)	Death
ALSO CONSIDER: Hemorrhage, GI – *Select*; Typhlitis (cecal inflammation).						
Esophagitis	Esophagitis	Asymptomatic pathologic, radiographic, or endoscopic findings only	Symptomatic; altered eating/swallowing (e.g., altered dietary habits, oral supplements); IV fluids indicated <24 hrs	Symptomatic and severely altered eating/swallowing (e.g., inadequate oral caloric or fluid intake); IV fluids, tube feedings, or TPN indicated ≥24 hrs	Life-threatening consequences	Death
REMARK: Esophagitis includes reflux esophagitis.						
ALSO CONSIDER: Dysphagia (difficulty swallowing).						

GASTROINTESTINAL

Adverse Event	Short Name	Grade 1	Grade 2	Grade 3	Grade 4	Grade 5
Fistula, GI – *Select*:<br>– Abdomen NOS<br>– Anus<br>– Biliary tree<br>– Colon/cecum/appendix<br>– Duodenum<br>– Esophagus<br>– Gallbladder<br>– Ileum<br>– Jejunum<br>– Oral cavity<br>– Pancreas<br>– Pharynx<br>– Rectum<br>– Salivary gland<br>– Small bowel NOS<br>– Stomach	Fistula, GI – *Select*	Asymptomatic, radiographic findings only	Symptomatic; altered GI function (e.g., altered dietary habits, diarrhea, or GI fluid loss); IV fluids indicated <24 hrs	Symptomatic and severely altered GI function (e.g., altered dietary habits, diarrhea, or GI fluid loss); IV fluids, tube feedings, or TPN indicated ≥24 hrs	Life-threatening consequences	Death
REMARK: A fistula is defined as an abnormal communication between two body cavities, potential spaces, and/or the skin. The site indicated for a fistula should be the site from which the abnormal process is believed to have originated. For example, a tracheo-esophageal fistula arising in the context of a resected or irradiated esophageal cancer is graded as Fistula, GI – esophagus.						
Flatulence	Flatulence	Mild	Moderate	—	—	—
Gastritis (including bile reflux gastritis)	Gastritis	Asymptomatic radiographic or endoscopic findings only	Symptomatic; altered gastric function (e.g., inadequate oral caloric or fluid intake); IV fluids indicated <24 hrs	Symptomatic and severely altered gastric function (e.g., inadequate oral caloric or fluid intake); IV fluids, tube feedings, or TPN indicated ≥24 hrs	Life-threatening consequences; operative intervention requiring complete organ resection (e.g., gastrectomy)	Death
ALSO CONSIDER: Hemorrhage, GI – *Select*; Ulcer, GI – *Select*.						
NAVIGATION NOTE: Head and neck soft tissue necrosis is graded as Soft tissue necrosis – *Select* in the MUSCULOSKELETAL/SOFT TISSUE CATEGORY.						
Heartburn/dyspepsia	Heartburn	Mild	Moderate	Severe	—	—
Hemorrhoids	Hemorrhoids	Asymptomatic	Symptomatic; banding or medical intervention indicated	Interfering with ADL; interventional radiology, endoscopic, or operative intervention indicated	Life-threatening consequences	Death

		Grade				
**Adverse Event**	**Short Name**	**1**	**2**	**3**	**4**	**5**
Ileus, GI (functional obstruction of bowel, i.e., neuroconstipation)	Ileus	Asymptomatic, radiographic findings only	Symptomatic; altered GI function (e.g., altered dietary habits); IV fluids indicated <24 hrs	Symptomatic and severely altered GI function; IV fluids, tube feeding, or TPN indicated ≥24 hrs	Life-threatening consequences	Death
REMARK: Ileus, GI is to be used for altered upper or lower GI function (e.g., delayed gastric or colonic emptying).<br>ALSO CONSIDER: Constipation; Nausea; Obstruction, GI – *Select*; Vomiting.						
Incontinence, anal	Incontinence, anal	Occasional use of pads required	Daily use of pads required	Interfering with ADL; operative intervention indicated	Permanent bowel diversion indicated	Death
REMARK: Incontinence, anal is to be used for loss of sphincter control as sequelae of operative or therapeutic intervention.						
Leak (including anastomotic), GI – *Select*:<br>– Biliary tree<br>– Esophagus<br>– Large bowel<br>– Leak NOS<br>– Pancreas<br>– Pharynx<br>– Rectum<br>– Small bowel<br>– Stoma<br>– Stomach	Leak, GI – *Select*	Asymptomatic radiographic findings only	Symptomatic; medical intervention indicated	Symptomatic and interfering with GI function; invasive or endoscopic intervention indicated	Life-threatening consequences	Death
REMARK: Leak (including anasomotic), GI – *Select* is to be used for clinical signs/symptoms or radiographic confirmation of anastomotic or conduit leak (e.g., biliary, esophageal, intestinal, pancreatic, pharyngeal, rectal), but without development of fistula.						
Malabsorption	Malabsorption	—	Altered diet; oral therapies indicated (e.g., enzymes, medications, dietary supplements)	Inability to aliment adequately via GI tract (i.e., TPN indicated)	Life-threatening consequences	Death

GASTROINTESTINAL

		Grade				
**Adverse Event**	**Short Name**	**1**	**2**	**3**	**4**	**5**
Mucositis/stomatitis (clinical exam) – *Select*:<br>– Anus<br>– Esophagus<br>– Large bowel<br>– Larynx<br>– Oral cavity<br>– Pharynx<br>– Rectum<br>– Small bowel<br>– Stomach<br>– Trachea	Mucositis (clinical exam) – *Select*	Erythema of the mucosa	Patchy ulcerations or pseudomembranes	Confluent ulcerations or pseudomembranes; bleeding with minor trauma	Tissue necrosis; significant spontaneous bleeding; life-threatening consequences	Death
REMARK: Mucositis/stomatitis (functional/symptomatic) may be used for mucositis of the upper aero-digestive tract caused by radiation, agents, or GVHD.						
Mucositis/stomatitis (functional/symptomatic) – *Select*:<br>– Anus<br>– Esophagus<br>– Large bowel<br>– Larynx<br>– Oral cavity<br>– Pharynx<br>– Rectum<br>– Small bowel<br>– Stomach<br>– Trachea	Mucositis (functional/ symptomatic) – *Select*	Upper aerodigestive tract sites: Minimal symptoms, normal diet; minimal respiratory symptoms but not interfering with function<br><br>Lower GI sites: Minimal discomfort, intervention not indicated	Upper aerodigestive tract sites: Symptomatic but can eat and swallow modified diet; respiratory symptoms interfering with function but not interfering with ADL<br><br>Lower GI sites: Symptomatic, medical intervention indicated but not interfering with ADL	Upper aerodigestive tract sites: Symptomatic and unable to adequately aliment or hydrate orally; respiratory symptoms interfering with ADL<br><br>Lower GI sites: Stool incontinence or other symptoms interfering with ADL	Symptoms associated with life-threatening consequences	Death
Nausea	Nausea	Loss of appetite without alteration in eating habits	Oral intake decreased without significant weight loss, dehydration or malnutrition; IV fluids indicated <24 hrs	Inadequate oral caloric or fluid intake; IV fluids, tube feedings, or TPN indicated ≥24 hrs	Life-threatening consequences	Death
ALSO CONSIDER: Anorexia; Vomiting.						

		Grade				
**Adverse Event**	**Short Name**	**1**	**2**	**3**	**4**	**5**
Necrosis, GI – *Select*:<br>– Anus<br>– Colon/cecum/appendix<br>– Duodenum<br>– Esophagus<br>– Gallbladder<br>– Hepatic<br>– Ileum<br>– Jejunum<br>– Oral<br>– Pancreas<br>– Peritoneal cavity<br>– Pharynx<br>– Rectum<br>– Small bowel NOS<br>– Stoma<br>– Stomach	Necrosis, GI – *Select*	—	—	Inability to aliment adequately by GI tract (e.g., requiring enteral or parenteral nutrition); interventional radiology, endoscopic, or operative intervention indicated	Life-threatening consequences; operative intervention requiring complete organ resection (e.g., total colectomy)	Death
ALSO CONSIDER: Visceral arterial ischemia (non-myocardial).						
Obstruction, GI – *Select*:<br>– Cecum<br>– Colon<br>– Duodenum<br>– Esophagus<br>– Gallbladder<br>– Ileum<br>– Jejunum<br>– Rectum<br>– Small bowel NOS<br>– Stoma<br>– Stomach	Obstruction, GI – *Select*	Asymptomatic radiographic findings only	Symptomatic; altered GI function (e.g., altered dietary habits, vomiting, diarrhea, or GI fluid loss); IV fluids indicated <24 hrs	Symptomatic and severely altered GI function (e.g., altered dietary habits, vomiting, diarrhea, or GI fluid loss); IV fluids, tube feedings, or TPN indicated ≥24 hrs; operative intervention indicated	Life-threatening consequences; operative intervention requiring complete organ resection (e.g., total colectomy)	Death
NAVIGATION NOTE: Operative injury is graded as Intra-operative injury – *Select Organ or Structure* in the SURGERY/INTRA-OPERATIVE INJURY CATEGORY.						
NAVIGATION NOTE: Pelvic pain is graded as Pain – *Select* in the PAIN CATEGORY.						

		Grade				
**Adverse Event**	**Short Name**	**1**	**2**	**3**	**4**	**5**
Perforation, GI – *Select*:<br>– Appendix<br>– Biliary tree<br>– Cecum<br>– Colon<br>– Duodenum<br>– Esophagus<br>– Gallbladder<br>– Ileum<br>– Jejunum<br>– Rectum<br>– Small bowel NOS<br>– Stomach	Perforation, GI – *Select*	Asymptomatic radiographic findings only	Medical intervention indicated; IV fluids indicated <24 hrs	IV fluids, tube feedings, or TPN indicated ≥24 hrs; operative intervention indicated	Life-threatening consequences	Death
Proctitis	Proctitis	Rectal discomfort, intervention not indicated	Symptoms not interfering with ADL; medical intervention indicated	Stool incontinence or other symptoms interfering with ADL; operative intervention indicated	Life-threatening consequences (e.g., perforation)	Death
Prolapse of stoma, GI	Prolapse of stoma, GI	Asymptomatic	Extraordinary local care or maintenance; minor revision indicated	Dysfunctional stoma; major revision indicated	Life-threatening consequences	Death
REMARK: Other stoma complications may be graded as Fistula, GI – *Select*; Leak (including anastomotic), GI – *Select*; Obstruction, GI – *Select;* Perforation, GI – *Select*; Stricture/stenosis (including anastomotic), GI – *Select*.						
NAVIGATION NOTE: Rectal or perirectal pain (proctalgia) is graded as Pain – *Select* in the PAIN CATEGORY.						
Salivary gland changes/saliva	Salivary gland changes	Slightly thickened saliva; slightly altered taste (e.g., metallic)	Thick, ropy, sticky saliva; markedly altered taste; alteration in diet indicated; secretion-induced symptoms not interfering with ADL	Acute salivary gland necrosis; severe secretion-induced symptoms interfering with ADL	Disabling	—
ALSO CONSIDER: Dry mouth/salivary gland (xerostomia); Mucositis/stomatitis (clinical exam) – *Select*; Mucositis/stomatitis (functional/symptomatic) – *Select*; Taste alteration (dysgeusia).						
NAVIGATION NOTE: Splenic function is graded in the BLOOD/BONE MARROW CATEGORY.						

GASTROINTESTINAL

		Grade				
**Adverse Event**	**Short Name**	**1**	**2**	**3**	**4**	**5**
Stricture/stenosis (including anastomotic), GI – *Select*:<br>– Anus<br>– Biliary tree<br>– Cecum<br>– Colon<br>– Duodenum<br>– Esophagus<br>– Ileum<br>– Jejunum<br>– Pancreas/pancreatic duct<br>– Pharynx<br>– Rectum<br>– Small bowel NOS<br>– Stoma<br>– Stomach	Stricture, GI – *Select*	Asymptomatic radiographic findings only	Symptomatic; altered GI function (e.g., altered dietary habits, vomiting, bleeding, diarrhea); IV fluids indicated <24 hrs	Symptomatic and severely altered GI function (e.g., altered dietary habits, diarrhea, or GI fluid loss); IV fluids, tube feedings, or TPN indicated ≥24 hrs; operative intervention indicated	Life-threatening consequences; operative intervention requiring complete organ resection (e.g., total colectomy)	Death
Taste alteration (dysgeusia)	Taste alteration	Altered taste but no change in diet	Altered taste with change in diet (e.g., oral supplements); noxious or unpleasant taste; loss of taste	—	—	—
Typhlitis (cecal inflammation)	Typhlitis	Asymptomatic, pathologic or radiographic findings only	Abdominal pain; mucus or blood in stool	Abdominal pain, fever, change in bowel habits with ileus; peritoneal signs	Life-threatening consequences (e.g., perforation, bleeding, ischemia, necrosis); operative intervention indicated	Death
ALSO CONSIDER: Colitis; Hemorrhage, GI – *Select* ; Ileus, GI (functional obstruction of bowel, i.e., neuroconstipation).						

GASTROINTESTINAL

		Grade				
**Adverse Event**	**Short Name**	**1**	**2**	**3**	**4**	**5**
Ulcer, GI<br>– *Select*:<br>– Anus<br>– Cecum<br>– Colon<br>– Duodenum<br>– Esophagus<br>– Ileum<br>– Jejunum<br>– Rectum<br>– Small bowel NOS<br>– Stoma<br>– Stomach	Ulcer, GI – *Select*	Asymptomatic, radiographic or endoscopic findings only	Symptomatic; altered GI function (e.g., altered dietary habits, oral supplements); IV fluids indicated <24 hrs	Symptomatic and severely altered GI function (e.g., inadequate oral caloric or fluid intake); IV fluids, tube feedings, or TPN indicated ≥24 hrs	Life-threatening consequences	Death
ALSO CONSIDER: Hemorrhage, GI – *Select*.						
Vomiting	Vomiting	1 episode in 24 hrs	2 – 5 episodes in 24 hrs; IV fluids indicated <24 hrs	≥6 episodes in 24 hrs; IV fluids, or TPN indicated ≥24 hrs	Life-threatening consequences	Death
ALSO CONSIDER: Dehydration.						
Gastrointestinal – Other (Specify, __)	GI – Other (Specify)	Mild	Moderate	Severe	Life-threatening; disabling	Death

GROWTH AND DEVELOPMENT

		Grade				
**Adverse Event**	**Short Name**	**1**	**2**	**3**	**4**	**5**
Bone age (alteration in bone age)	Bone age	—	±2 SD (standard deviation) from normal	—	—	—
Bone growth: femoral head; slipped capital femoral epiphysis	Femoral head growth	Mild valgus/varus deformity	Moderate valgus/varus deformity, symptomatic, interfering with function but not interfering with ADL	Mild slipped capital femoral epiphysis; operative intervention (e.g., fixation) indicated; interfering with ADL	Disabling; severe slipped capital femoral epiphysis >60%; avascular necrosis	—
Bone growth: limb length discrepancy	Limb length	Mild length discrepancy <2 cm	Moderate length discrepancy 2 – 5 cm; shoe lift indicated	Severe length discrepancy >5 cm; operative intervention indicated; interfering with ADL	Disabling; epiphysiodesis	—
Bone growth: spine kyphosis/lordosis	Kyphosis/lordosis	Mild radiographic changes	Moderate accentuation; interfering with function but not interfering with ADL	Severe accentuation; operative intervention indicated; interfering with ADL	Disabling (e.g., cannot lift head)	—
Growth velocity (reduction in growth velocity)	Reduction in growth velocity	10 – 29% reduction in growth from the baseline growth curve	30 – 49% reduction in growth from the baseline growth curve	≥50% reduction in growth from the baseline growth curve	—	—
Puberty (delayed)	Delayed puberty	—	No breast development by age 13 yrs for females; no Tanner Stage 2 development by age 14.5 yrs for males	No sexual development by age 14 yrs for girls, age 16 yrs for boys; hormone replacement indicated	—	—
REMARK: Do not use testicular size for Tanner Stage in male cancer survivors.						
Puberty (precocious)	Precocious puberty	—	Physical signs of puberty <7 years for females, <9 years for males	—	—	—
Short stature	Short stature	Beyond two standard deviations of age and gender mean height	Altered ADL	—	—	—
REMARK: Short stature is secondary to growth hormone deficiency.<br>ALSO CONSIDER: Neuroendocrine: growth hormone secretion abnormality.						
Growth and Development – Other (Specify, __)	Growth and Development – Other (Specify)	Mild	Moderate	Severe	Life-threatening; disabling	Death

HEMORRHAGE/BLEEDING

		Grade				
**Adverse Event**	**Short Name**	**1**	**2**	**3**	**4**	**5**
Hematoma	Hematoma	Minimal symptoms, invasive intervention not indicated	Minimally invasive evacuation or aspiration indicated	Transfusion, interventional radiology, or operative intervention indicated	Life-threatening consequences; major urgent intervention indicated	Death
REMARK: Hematoma refers to extravasation at wound or operative site or secondary to other intervention. Transfusion implies pRBC.						
ALSO CONSIDER: Fibrinogen; INR (International Normalized Ratio of prothrombin time); Platelets; PTT (Partial Thromboplastin Time).						
Hemorrhage/bleeding associated with surgery, intra-operative or postoperative	Hemorrhage with surgery	—	—	Requiring transfusion of 2 units non-autologous (10 cc/kg for pediatrics) pRBCs beyond protocol specification; postoperative interventional radiology, endoscopic, or operative intervention indicated	Life-threatening consequences	Death
REMARK: Postoperative period is defined as ≤72 hours after surgery. Verify protocol-specific acceptable guidelines regarding pRBC transfusion.						
ALSO CONSIDER: Fibrinogen; INR (International Normalized Ratio of prothrombin time); Platelets; PTT (Partial Thromboplastin Time).						
Hemorrhage, CNS	CNS hemorrhage	Asymptomatic, radiographic findings only	Medical intervention indicated	Ventriculostomy, ICP monitoring, intraventricular thrombolysis, or operative intervention indicated	Life-threatening consequences; neurologic deficit or disability	Death
ALSO CONSIDER: Fibrinogen; INR (International Normalized Ratio of prothrombin time); Platelets; PTT (Partial Thromboplastin Time).						

HEMORRHAGE/BLEEDING

		Grade				
**Adverse Event**	**Short Name**	**1**	**2**	**3**	**4**	**5**
Hemorrhage, GI – *Select*:<br>– Abdomen NOS<br>– Anus<br>– Biliary tree<br>– Cecum/appendix<br>– Colon<br>– Duodenum<br>– Esophagus<br>– Ileum<br>– Jejunum<br>– Liver<br>– Lower GI NOS<br>– Oral cavity<br>– Pancreas<br>– Peritoneal cavity<br>– Rectum<br>– Stoma<br>– Stomach<br>– Upper GI NOS<br>– Varices (esophageal)<br>– Varices (rectal)	Hemorrhage, GI – *Select*	Mild, intervention (other than iron supplements) not indicated	Symptomatic and medical intervention or minor cauterization indicated	Transfusion, interventional radiology, endoscopic, or operative intervention indicated; radiation therapy (i.e., hemostasis of bleeding site)	Life-threatening consequences; major urgent intervention indicated	Death

REMARK: Transfusion implies pRBC.

ALSO CONSIDER: Fibrinogen; INR (International Normalized Ratio of prothrombin time); Platelets; PTT (Partial Thromboplastin Time).

HEMORRHAGE/BLEEDING

		Grade				
**Adverse Event**	**Short Name**	**1**	**2**	**3**	**4**	**5**
Hemorrhage, GU – *Select*:<br>– Bladder<br>– Fallopian tube<br>– Kidney<br>– Ovary<br>– Prostate<br>– Retroperitoneum<br>– Spermatic cord<br>– Stoma<br>– Testes<br>– Ureter<br>– Urethra<br>– Urinary NOS<br>– Uterus<br>– Vagina<br>– Vas deferens	Hemorrhage, GU – *Select*	Minimal or microscopic bleeding; intervention not indicated	Gross bleeding, medical intervention, or urinary tract irrigation indicated	Transfusion, interventional radiology, endoscopic, or operative intervention indicated; radiation therapy (i.e., hemostasis of bleeding site)	Life-threatening consequences; major urgent intervention indicated	Death
REMARK: Transfusion implies pRBC.						
ALSO CONSIDER: Fibrinogen; INR (International Normalized Ratio of prothrombin time); Platelets; PTT (Partial Thromboplastin Time).						
Hemorrhage, pulmonary/ upper respiratory – *Select*:<br>– Bronchopulmonary NOS<br>– Bronchus<br>– Larynx<br>– Lung<br>– Mediastinum<br>– Nose<br>– Pharynx<br>– Pleura<br>– Respiratory tract NOS<br>– Stoma<br>– Trachea	Hemorrhage pulmonary – *Select*	Mild, intervention not indicated	Symptomatic and medical intervention indicated	Transfusion, interventional radiology, endoscopic, or operative intervention indicated; radiation therapy (i.e., hemostasis of bleeding site)	Life-threatening consequences; major urgent intervention indicated	Death
REMARK: Transfusion implies pRBC.						
ALSO CONSIDER: Fibrinogen; INR (International Normalized Ratio of prothrombin time); Platelets; PTT (Partial Thromboplastin Time).						
Petechiae/purpura (hemorrhage/bleeding into skin or mucosa)	Petechiae	Few petechiae	Moderate petechiae; purpura	Generalized petechiae or purpura	—	—
ALSO CONSIDER: Fibrinogen; INR (International Normalized Ratio of prothrombin time); Platelets; PTT (Partial Thromboplastin Time).						

HEMORRHAGE/BLEEDING						
		Grade				
**Adverse Event**	**Short Name**	**1**	**2**	**3**	**4**	**5**
NAVIGATION NOTE: Vitreous hemorrhage is graded in the OCULAR/VISUAL CATEGORY.						
Hemorrhage/Bleeding – Other (Specify, __)	Hemorrhage – Other (Specify)	Mild without transfusion	—	Transfusion indicated	Catastrophic bleeding, requiring major non-elective intervention	Death

HEPATOBILIARY/PANCREAS

		Grade				
**Adverse Event**	**Short Name**	**1**	**2**	**3**	**4**	**5**
NAVIGATION NOTE: Biliary tree damage is graded as Fistula, GI – *Select*; Leak (including anastomotic), GI – *Select*; Necrosis, GI – *Select*; Obstruction, GI – *Select*; Perforation, GI – *Select*; Stricture/stenosis (including anastomotic), GI – *Select* in the GASTROINTESTINAL CATEGORY.						
Cholecystitis	Cholecystitis	Asymptomatic, radiographic findings only	Symptomatic, medical intervention indicated	Interventional radiology, endoscopic, or operative intervention indicated	Life-threatening consequences (e.g., sepsis or perforation)	Death
ALSO CONSIDER: Infection (documented clinically or microbiologically) with Grade 3 or 4 neutrophils – *Select*; Infection with normal ANC or Grade 1 or 2 neutrophils – *Select*; Infection with unknown ANC – *Select*.						
Liver dysfunction/failure (clinical)	Liver dysfunction	—	Jaundice	Asterixis	Encephalopathy or coma	Death
REMARK: Jaundice is not an AE, but occurs when the liver is not working properly or when a bile duct is blocked. It is graded as a result of liver dysfunction/failure or elevated bilirubin.<br>ALSO CONSIDER: Bilirubin (hyperbilirubinemia).						
Pancreas, exocrine enzyme deficiency	Pancreas, exocrine enzyme deficiency	—	Increase in stool frequency, bulk, or odor; steatorrhea	Sequelae of absorption deficiency (e.g., weight loss)	Life-threatening consequences	Death
ALSO CONSIDER: Diarrhea.						
Pancreatitis	Pancreatitis	Asymptomatic, enzyme elevation and/or radiographic findings	Symptomatic, medical intervention indicated	Interventional radiology or operative intervention indicated	Life-threatening consequences (e.g., circulatory failure, hemorrhage, sepsis)	Death
ALSO CONSIDER: Amylase.						
NAVIGATION NOTE: Stricture (biliary tree, hepatic or pancreatic) is graded as Stricture/stenosis (including anastomotic), GI – *Select* in the GASTROINTESTINAL CATEGORY.						
Hepatobiliary/Pancreas – Other (Specify, __)	Hepatobiliary – Other (Specify)	Mild	Moderate	Severe	Life-threatening; disabling	Death

INFECTION

		Grade				
**Adverse Event**	**Short Name**	**1**	**2**	**3**	**4**	**5**
Colitis, infectious (e.g., Clostridium difficile)	Colitis, infectious	Asymptomatic, pathologic or radiographic findings only	Abdominal pain with mucus and/or blood in stool	IV antibiotics or TPN indicated	Life-threatening consequences (e.g., perforation, bleeding, ischemia, necrosis or toxic megacolon); operative resection or diversion indicated	Death
ALSO CONSIDER: Hemorrhage, GI – *Select*; Typhlitis (cecal inflammation).						
Febrile neutropenia (fever of unknown origin without clinically or microbiologically documented infection) (ANC <1.0 x 10^9/L, fever ≥38.5°C)	Febrile neutropenia	—	—	Present	Life-threatening consequences (e.g., septic shock, hypotension, acidosis, necrosis)	Death
ALSO CONSIDER: Neutrophils/granulocytes (ANC/AGC).						
Infection (documented clinically or microbiologically) with Grade 3 or 4 neutrophils (ANC <1.0 x 10^9/L) – *Select*<br>*'Select'* AEs appear at the end of the CATEGORY.	Infection (documented clinically) with Grade 3 or 4 ANC – *Select*	—	Localized, local intervention indicated	IV antibiotic, antifungal, or antiviral intervention indicated; interventional radiology or operative intervention indicated	Life-threatening consequences (e.g., septic shock, hypotension, acidosis, necrosis)	Death
REMARK: Fever with Grade 3 or 4 neutrophils in the absence of documented infection is graded as Febrile neutropenia (fever of unknown origin without clinically or microbiologically documented infection).<br>ALSO CONSIDER: Neutrophils/granulocytes (ANC/AGC).						
Infection with normal ANC or Grade 1 or 2 neutrophils – *Select*<br>'*Select*' AEs appear at the end of the CATEGORY.	Infection with normal ANC – *Select*	—	Localized, local intervention indicated	IV antibiotic, antifungal, or antiviral intervention indicated; interventional radiology or operative intervention indicated	Life-threatening consequences (e.g., septic shock, hypotension, acidosis, necrosis)	Death

		Grade				
**Adverse Event**	**Short Name**	**1**	**2**	**3**	**4**	**5**
Infection with unknown ANC – *Select*<br>*'Select'* AEs appear at the end of the CATEGORY.	Infection with unknown ANC – *Select*	—	Localized, local intervention indicated	IV antibiotic, antifungal, or antiviral intervention indicated; interventional radiology or operative intervention indicated	Life-threatening consequences (e.g., septic shock, hypotension, acidosis, necrosis)	Death
REMARK: Infection with unknown ANC – *Select* is to be used in the rare case when ANC is unknown.						
Opportunistic infection associated with ≥Grade 2 Lymphopenia	Opportunistic infection	—	Localized, local intervention indicated	IV antibiotic, antifungal, or antiviral intervention indicated; interventional radiology or operative intervention indicated	Life-threatening consequences (e.g., septic shock, hypotension, acidosis, necrosis)	Death
ALSO CONSIDER: Lymphopenia.						
Viral hepatitis	Viral hepatitis	Present; transaminases and liver function normal	Transaminases abnormal, liver function normal	Symptomatic liver dysfunction; fibrosis by biopsy; compensated cirrhosis	Decompensated liver function (e.g., ascites, coagulopathy, encephalopathy, coma)	Death
REMARK: Non-viral hepatitis is graded as Infection – *Select*.<br>ALSO CONSIDER: Albumin, serum-low (hypoalbuminemia); ALT, SGPT (serum glutamic pyruvic transaminase); AST, SGOT (serum glutamic oxaloacetic transaminase); Bilirubin (hyperbilirubinemia); Encephalopathy.						
Infection – Other (Specify, __)	Infection – Other (Specify)	Mild	Moderate	Severe	Life-threatening; disabling	Death

INFECTION – *SELECT*

AUDITORY/EAR
- External ear (otitis externa)
- Middle ear (otitis media)

CARDIOVASCULAR
- Artery
- Heart (endocarditis)
- Spleen
- Vein

DERMATOLOGY/SKIN
- Lip/perioral
- Peristomal
- Skin (cellulitis)
- Ungual (nails)

GASTROINTESTINAL
- Abdomen NOS
- Anal/perianal
- Appendix
- Cecum
- Colon
- Dental-tooth
- Duodenum
- Esophagus
- Ileum
- Jejunum
- Oral cavity-gums (gingivitis)
- Peritoneal cavity
- Rectum
- Salivary gland
- Small bowel NOS
- Stomach

GENERAL
- Blood
- Catheter-related
- Foreign body (e.g., graft, implant, prosthesis, stent)
- Wound

HEPATOBILIARY/PANCREAS
- Biliary tree
- Gallbladder (cholecystitis)
- Liver
- Pancreas

LYMPHATIC
- Lymphatic

MUSCULOSKELETAL
- Bone (osteomyelitis)
- Joint
- Muscle (infection myositis)
- Soft tissue NOS

NEUROLOGY
- Brain (encephalitis, infectious)
- Brain + Spinal cord (encephalomyelitis)
- Meninges (meningitis)
- Nerve-cranial
- Nerve-peripheral
- Spinal cord (myelitis)

OCULAR
- Conjunctiva
- Cornea
- Eye NOS
- Lens

PULMONARY/UPPER RESPIRATORY
- Bronchus
- Larynx
- Lung (pneumonia)
- Mediastinum NOS
- Mucosa
- Neck NOS
- Nose
- Paranasal
- Pharynx
- Pleura (empyema)
- Sinus
- Trachea
- Upper aerodigestive NOS
- Upper airway NOS

RENAL/GENITOURINARY
- Bladder (urinary)
- Kidney
- Prostate
- Ureter
- Urethra
- Urinary tract NOS

SEXUAL/REPRODUCTIVE FUNCTION
- Cervix
- Fallopian tube
- Pelvis NOS
- Penis
- Scrotum
- Uterus
- Vagina
- Vulva

LYMPHATICS

		Grade				
**Adverse Event**	**Short Name**	**1**	**2**	**3**	**4**	**5**
Chyle or lymph leakage	Chyle or lymph leakage	Asymptomatic, clinical or radiographic findings	Symptomatic, medical intervention indicated	Interventional radiology or operative intervention indicated	Life-threatening complications	Death
ALSO CONSIDER: Chylothorax.						
Dermal change lymphedema, phlebolymphedema	Dermal change	Trace thickening or faint discoloration	Marked discoloration; leathery skin texture; papillary formation	—	—	—
REMARK: Dermal change lymphedema, phlebolymphedema refers to changes due to venous stasis.<br>ALSO CONSIDER: Ulceration.						
Edema: head and neck	Edema: head and neck	Localized to dependent areas, no disability or functional impairment	Localized facial or neck edema with functional impairment	Generalized facial or neck edema with functional impairment (e.g., difficulty in turning neck or opening mouth compared to baseline)	Severe with ulceration or cerebral edema; tracheotomy or feeding tube indicated	Death
Edema: limb	Edema: limb	5 – 10% inter-limb discrepancy in volume or circumference at point of greatest visible difference; swelling or obscuration of anatomic architecture on close inspection; pitting edema	>10 – 30% inter-limb discrepancy in volume or circumference at point of greatest visible difference; readily apparent obscuration of anatomic architecture; obliteration of skin folds; readily apparent deviation from normal anatomic contour	>30% inter-limb discrepancy in volume; lymphorrhea; gross deviation from normal anatomic contour; interfering with ADL	Progression to malignancy (i.e., lymphangiosarcoma); amputation indicated; disabling	Death
Edema: trunk/genital	Edema: trunk/genital	Swelling or obscuration of anatomic architecture on close inspection; pitting edema	Readily apparent obscuration of anatomic architecture; obliteration of skin folds; readily apparent deviation from normal anatomic contour	Lymphorrhea; interfering with ADL; gross deviation from normal anatomic contour	Progression to malignancy (i.e., lymphangiosarcoma); disabling	Death
Edema: viscera	Edema: viscera	Asymptomatic; clinical or radiographic findings only	Symptomatic; medical intervention indicated	Symptomatic and unable to aliment adequately orally; interventional radiology or operative intervention indicated	Life-threatening consequences	Death

LYMPHATICS						
		Grade				
**Adverse Event**	**Short Name**	**1**	**2**	**3**	**4**	**5**
Lymphedema-related fibrosis	Lymphedema-related fibrosis	Minimal to moderate redundant soft tissue, unresponsive to elevation or compression, with moderately firm texture or spongy feel	Marked increase in density and firmness, with or without tethering	Very marked density and firmness with tethering affecting ≥40% of the edematous area	—	—
Lymphocele	Lymphocele	Asymptomatic, clinical or radiographic findings only	Symptomatic; medical intervention indicated	Symptomatic and interventional radiology or operative intervention indicated	—	—
Phlebolymphatic cording	Phlebolymphatic cording	Asymptomatic, clinical findings only	Symptomatic; medical intervention indicated	Symptomatic and leading to contracture or reduced range of motion	—	—
Lymphatics – Other (Specify, __)	Lymphatics – Other (Specify)	Mild	Moderate	Severe	Life-threatening; disabling	Death

METABOLIC/LABORATORY

		Grade				
**Adverse Event**	**Short Name**	**1**	**2**	**3**	**4**	**5**
Acidosis (metabolic or respiratory)	Acidosis	pH <normal, but ≥7.3	—	pH <7.3	pH <7.3 with life-threatening consequences	Death
Albumin, serum-low (hypoalbuminemia)	Hypoalbuminemia	<LLN – 3 g/dL<br><LLN – 30 g/L	<3 – 2 g/dL<br><30 – 20 g/L	<2 g/dL<br><20 g/L	—	Death
Alkaline phosphatase	Alkaline phosphatase	>ULN – 2.5 x ULN	>2.5 – 5.0 x ULN	>5.0 – 20.0 x ULN	>20.0 x ULN	—
Alkalosis (metabolic or respiratory)	Alkalosis	pH >normal, but ≤7.5	—	pH >7.5	pH >7.5 with life-threatening consequences	Death
ALT, SGPT (serum glutamic pyruvic transaminase)	ALT	>ULN – 2.5 x ULN	>2.5 – 5.0 x ULN	>5.0 – 20.0 x ULN	>20.0 x ULN	—
Amylase	Amylase	>ULN – 1.5 x ULN	>1.5 – 2.0 x ULN	>2.0 – 5.0 x ULN	>5.0 x ULN	—
AST, SGOT (serum glutamic oxaloacetic transaminase)	AST	>ULN – 2.5 x ULN	>2.5 – 5.0 x ULN	>5.0 – 20.0 x ULN	>20.0 x ULN	—
Bicarbonate, serum-low	Bicarbonate, serum-low	<LLN – 16 mmol/L	<16 – 11 mmol/L	<11 – 8 mmol/L	<8 mmol/L	Death
Bilirubin (hyperbilirubinemia)	Bilirubin	>ULN – 1.5 x ULN	>1.5 – 3.0 x ULN	>3.0 – 10.0 x ULN	>10.0 x ULN	—
REMARK: Jaundice is not an AE, but may be a manifestation of liver dysfunction/failure or elevated bilirubin. If jaundice is associated with elevated bilirubin, grade bilirubin.						
Calcium, serum-low (hypocalcemia)	Hypocalcemia	<LLN – 8.0 mg/dL<br><LLN – 2.0 mmol/L<br><br>Ionized calcium:<br><LLN – 1.0 mmol/L	<8.0 – 7.0 mg/dL<br><2.0 – 1.75 mmol/L<br><br>Ionized calcium:<br><1.0 – 0.9 mmol/L	<7.0 – 6.0 mg/dL<br><1.75 – 1.5 mmol/L<br><br>Ionized calcium:<br><0.9 – 0.8 mmol/L	<6.0 mg/dL<br><1.5 mmol/L<br><br>Ionized calcium:<br><0.8 mmol/L	Death
REMARK: Calcium can be falsely low if hypoalbuminemia is present. Serum albumin is <4.0 g/dL, hypocalcemia is reported after the following corrective calculation has been performed: Corrected Calcium (mg/dL) = Total Calcium (mg/dL) – 0.8 [Albumin (g/dL) – 4] [4]. Alternatively, direct measurement of ionized calcium is the definitive method to diagnose metabolically relevant alterations in serum calcium.						

[4] Crit Rev Clin Lab Sci 1984;21(1):51-97

METABOLIC/LABORATORY

Adverse Event	Short Name	Grade 1	Grade 2	Grade 3	Grade 4	Grade 5
Calcium, serum-high (hypercalcemia)	Hypercalcemia	>ULN – 11.5 mg/dL<br>>ULN – 2.9 mmol/L<br><br>Ionized calcium:<br>>ULN – 1.5 mmol/L	>11.5 – 12.5 mg/dL<br>>2.9 – 3.1 mmol/L<br><br>Ionized calcium:<br>>1.5 – 1.6 mmol/L	>12.5 – 13.5 mg/dL<br>>3.1 – 3.4 mmol/L<br><br>Ionized calcium:<br>>1.6 – 1.8 mmol/L	>13.5 mg/dL<br>>3.4 mmol/L<br><br>Ionized calcium:<br>>1.8 mmol/L	Death
Cholesterol, serum-high (hypercholesteremia)	Cholesterol	>ULN – 300 mg/dL<br>>ULN – 7.75 mmol/L	>300 – 400 mg/dL<br>>7.75 – 10.34 mmol/L	>400 – 500 mg/dL<br>>10.34 – 12.92 mmol/L	>500 mg/dL<br>>12.92 mmol/L	Death
CPK (creatine phosphokinase)	CPK	>ULN – 2.5 x ULN	>2.5 x ULN – 5 x ULN	>5 x ULN – 10 x ULN	>10 x ULN	Death
Creatinine	Creatinine	>ULN – 1.5 x ULN	>1.5 – 3.0 x ULN	>3.0 – 6.0 x ULN	>6.0 x ULN	Death
REMARK: Adjust to age-appropriate levels for pediatric patients.<br>ALSO CONSIDER: Glomerular filtration rate.						
GGT (γ-Glutamyl transpeptidase)	GGT	>ULN – 2.5 x ULN	>2.5 – 5.0 x ULN	>5.0 – 20.0 x ULN	>20.0 x ULN	—
Glomerular filtration rate	GFR	<75 – 50% LLN	<50 – 25% LLN	<25% LLN, chronic dialysis not indicated	Chronic dialysis or renal transplant indicated	Death
ALSO CONSIDER: Creatinine.						
Glucose, serum-high (hyperglycemia)	Hyperglycemia	>ULN – 160 mg/dL<br>>ULN – 8.9 mmol/L	>160 – 250 mg/dL<br>>8.9 – 13.9 mmol/L	>250 – 500 mg/dL<br>>13.9 – 27.8 mmol/L	>500 mg/dL<br>>27.8 mmol/L or acidosis	Death
REMARK: Hyperglycemia, in general, is defined as fasting unless otherwise specified in protocol.						
Glucose, serum-low (hypoglycemia)	Hypoglycemia	<LLN – 55 mg/dL<br><LLN – 3.0 mmol/L	<55 – 40 mg/dL<br><3.0 – 2.2 mmol/L	<40 – 30 mg/dL<br><2.2 – 1.7 mmol/L	<30 mg/dL<br><1.7 mmol/L	Death
Hemoglobinuria	Hemoglobinuria	Present	—	—	—	Death
Lipase	Lipase	>ULN – 1.5 x ULN	>1.5 – 2.0 x ULN	>2.0 – 5.0 x ULN	>5.0 x ULN	—
Magnesium, serum-high (hypermagnesemia)	Hypermagnesemia	>ULN – 3.0 mg/dL<br>>ULN – 1.23 mmol/L	—	>3.0 – 8.0 mg/dL<br>>1.23 – 3.30 mmol/L	>8.0 mg/dL<br>>3.30 mmol/L	Death
Magnesium, serum-low (hypomagnesemia)	Hypomagnesemia	<LLN – 1.2 mg/dL<br><LLN – 0.5 mmol/L	<1.2 – 0.9 mg/dL<br><0.5 – 0.4 mmol/L	<0.9 – 0.7 mg/dL<br><0.4 – 0.3 mmol/L	<0.7 mg/dL<br><0.3 mmol/L	Death
Phosphate, serum-low (hypophosphatemia)	Hypophosphatemia	<LLN – 2.5 mg/dL<br><LLN – 0.8 mmol/L	<2.5 – 2.0 mg/dL<br><0.8 – 0.6 mmol/L	<2.0 – 1.0 mg/dL<br><0.6 – 0.3 mmol/L	<1.0 mg/dL<br><0.3 mmol/L	Death
Potassium, serum-high (hyperkalemia)	Hyperkalemia	>ULN – 5.5 mmol/L	>5.5 – 6.0 mmol/L	>6.0 – 7.0 mmol/L	>7.0 mmol/L	Death

METABOLIC/LABORATORY

		Grade				
**Adverse Event**	**Short Name**	**1**	**2**	**3**	**4**	**5**
Potassium, serum-low (hypokalemia)	Hypokalemia	<LLN – 3.0 mmol/L	—	<3.0 – 2.5 mmol/L	<2.5 mmol/L	Death
Proteinuria	Proteinuria	1+ or<br>0.15 – 1.0 g/24 hrs	2+ to 3+ or<br>>1.0 – 3.5 g/24 hrs	4+ or<br>>3.5 g/24 hrs	Nephrotic syndrome	Death
Sodium, serum-high (hypernatremia)	Hypernatremia	>ULN – 150 mmol/L	>150 – 155 mmol/L	>155 – 160 mmol/L	>160 mmol/L	Death
Sodium, serum-low (hyponatremia)	Hyponatremia	<LLN – 130 mmol/L	—	<130 – 120 mmol/L	<120 mmol/L	Death
Triglyceride, serum-high (hypertriglyceridemia)	Hypertriglyceridemia	>ULN – 2.5 x ULN	>2.5 – 5.0 x ULN	>5.0 – 10 x ULN	>10 x ULN	Death
Uric acid, serum-high (hyperuricemia)	Hyperuricemia	>ULN – 10 mg/dL<br>≤0.59 mmol/L without physiologic consequences	—	>ULN – 10 mg/dL<br>≤0.59 mmol/L with physiologic consequences	>10 mg/dL<br>>0.59 mmol/L	Death
ALSO CONSIDER: Creatinine; Potassium, serum-high (hyperkalemia); Renal failure; Tumor lysis syndrome.						
Metabolic/Laboratory – Other (Specify, __)	Metabolic/Lab – Other (Specify)	Mild	Moderate	Severe	Life-threatening; disabling	Death

MUSCULOSKELETAL/SOFT TISSUE

		Grade				
**Adverse Event**	**Short Name**	**1**	**2**	**3**	**4**	**5**
Arthritis (non-septic)	Arthritis	Mild pain with inflammation, erythema, or joint swelling, but not interfering with function	Moderate pain with inflammation, erythema, or joint swelling interfering with function, but not interfering with ADL	Severe pain with inflammation, erythema, or joint swelling and interfering with ADL	Disabling	Death
REMARK: Report only when the diagnosis of arthritis (e.g., inflammation of a joint or a state characterized by inflammation of joints) is made. Arthralgia (sign or symptom of pain in a joint, especially non-inflammatory in character) is graded as Pain – *Select* in the PAIN CATEGORY.						
Bone: spine-scoliosis	Scoliosis	≤20 degrees; clinically undetectable	>20 – 45 degrees; visible by forward flexion; interfering with function but not interfering with ADL	>45 degrees; scapular prominence in forward flexion; operative intervention indicated; interfering with ADL	Disabling (e.g., interfering with cardiopulmonary function)	Death
Cervical spine-range of motion	Cervical spine ROM	Mild restriction of rotation or flexion between 60 – 70 degrees	Rotation <60 degrees to right or left; <60 degrees of flexion	Ankylosed/fused over multiple segments with no C-spine rotation	—	—
REMARK: 60 – 65 degrees of rotation is required for reversing a car; 60 – 65 degrees of flexion is required to tie shoes.						
Exostosis	Exostosis	Asymptomatic	Involving multiple sites; pain or interfering with function	Excision indicated	Progression to malignancy (i.e., chondrosarcoma)	Death
Extremity-lower (gait/walking)	Gait/walking	Limp evident only to trained observer and able to walk ≥1 kilometer; cane indicated for walking	Noticeable limp, or limitation of limb function, but able to walk ≥0.1 kilometer (1 city block); quad cane indicated for walking	Severe limp with stride modified to maintain balance (widened base of support, marked reduction in step length); ambulation limited to walker; crutches indicated	Unable to walk	—
ALSO CONSIDER: Ataxia (incoordination); Muscle weakness, generalized or specific area (not due to neuropathy) – *Select*.						
Extremity-upper (function)	Extremity-upper (function)	Able to perform most household or work activities with affected limb	Able to perform most household or work activities with compensation from unaffected limb	Interfering with ADL	Disabling; no function of affected limb	—
Fibrosis-cosmesis	Fibrosis-cosmesis	Visible only on close examination	Readily apparent but not disfiguring	Significant disfigurement; operative intervention indicated if patient chooses	—	—

		Grade				
**Adverse Event**	**Short Name**	**1**	**2**	**3**	**4**	**5**
Fibrosis-deep connective tissue	Fibrosis-deep connective tissue	Increased density, "spongy" feel	Increased density with firmness or tethering	Increased density with fixation of tissue; operative intervention indicated; interfering with ADL	Life-threatening; disabling; loss of limb; interfering with vital organ function	Death
ALSO CONSIDER: Induration/fibrosis (skin and subcutaneous tissue); Muscle weakness, generalized or specific area (not due to neuropathy) – *Select*; Neuropathy: motor; Neuropathy: sensory.						
Fracture	Fracture	Asymptomatic, radiographic findings only (e.g., asymptomatic rib fracture on plain x-ray, pelvic insufficiency fracture on MRI, etc.)	Symptomatic but non-displaced; immobilization indicated	Symptomatic and displaced or open wound with bone exposure; operative intervention indicated	Disabling; amputation indicated	Death
Joint-effusion	Joint-effusion	Asymptomatic, clinical or radiographic findings only	Symptomatic; interfering with function but not interfering with ADL	Symptomatic and interfering with ADL	Disabling	Death
ALSO CONSIDER: Arthritis (non-septic).						
Joint-function[5]	Joint-function	Stiffness interfering with athletic activity; ≤25% loss of range of motion (ROM)	Stiffness interfering with function but not interfering with ADL; >25 – 50% decrease in ROM	Stiffness interfering with ADL; >50 – 75% decrease in ROM	Fixed or non-functional joint (arthrodesis); >75% decrease in ROM	—
ALSO CONSIDER: Arthritis (non-septic).						
Local complication – device/prosthesis-related	Device/prosthesis	Asymptomatic	Symptomatic, but not interfering with ADL; local wound care; medical intervention indicated	Symptomatic, interfering with ADL; operative intervention indicated (e.g., hardware/device replacement or removal, reconstruction)	Life-threatening; disabling; loss of limb or organ	Death
Lumbar spine-range of motion	Lumbar spine ROM	Stiffness and difficulty bending to the floor to pick up a very light object but able to do activity	Some lumbar spine flexion but requires a reaching aid to pick up a very light object from the floor	Ankylosed/fused over multiple segments with no L-spine flexion (i.e., unable to reach to floor to pick up a very light	—	—

[5] Adapted from the *International SFTR Method of Measuring and Recording Joint Motion, International Standard Orthopedic Measurements (ISOM),* Jon J. Gerhardt and Otto A. Russee, Bern, Switzerland, Han Huber 9 Publisher), 1975.

MUSCULOSKELETAL/SOFT TISSUE

		Grade				
**Adverse Event**	**Short Name**	**1**	**2**	**3**	**4**	**5**
				object)		
Muscle weakness, generalized or specific area (not due to neuropathy) – *Select*: – Extraocular – Extremity-lower – Extremity-upper – Facial – Left-sided – Ocular – Pelvic – Right-sided – Trunk – Whole body/generalized	Muscle weakness – *Select*	Asymptomatic, weakness on physical exam	Symptomatic and interfering with function, but not interfering with ADL	Symptomatic and interfering with ADL	Life-threatening; disabling	Death
ALSO CONSIDER: Fatigue (asthenia, lethargy, malaise).						
Muscular/skeletal hypoplasia	Muscular/skeletal hypoplasia	Cosmetically and functionally insignificant hypoplasia	Deformity, hypoplasia, or asymmetry able to be remediated by prosthesis (e.g., shoe insert) or covered by clothing	Functionally significant deformity, hypoplasia, or asymmetry, unable to be remediated by prosthesis or covered by clothing	Disabling	—
Myositis (inflammation/damage of muscle)	Myositis	Mild pain, not interfering with function	Pain interfering with function, but not interfering with ADL	Pain interfering with ADL	Disabling	Death
REMARK: Myositis implies muscle damage (i.e., elevated CPK).						
ALSO CONSIDER: CPK (creatine phosphokinase); Pain – *Select*.						
Osteonecrosis (avascular necrosis)	Osteonecrosis	Asymptomatic, radiographic findings only	Symptomatic and interfering with function, but not interfering with ADL; minimal bone removal indicated (i.e., minor sequestrectomy)	Symptomatic and interfering with ADL; operative intervention or hyperbaric oxygen indicated	Disabling	Death

MUSCULOSKELETAL/SOFT TISSUE

		Grade				
**Adverse Event**	**Short Name**	**1**	**2**	**3**	**4**	**5**
Osteoporosis[6]	Osteoporosis	Radiographic evidence of osteoporosis or Bone Mineral Density (BMD) t-score –1 to –2.5 (osteopenia) and no loss of height or therapy indicated	BMD t-score < –2.5; loss of height <2 cm; anti-osteoporotic therapy indicated	Fractures; loss of height ≥2 cm	Disabling	Death
Seroma	Seroma	Asymptomatic	Symptomatic; medical intervention or simple aspiration indicated	Symptomatic, interventional radiology or operative intervention indicated	—	—
Soft tissue necrosis – *Select*:<br>– Abdomen<br>– Extremity-lower<br>– Extremity-upper<br>– Head<br>– Neck<br>– Pelvic<br>– Thorax	Soft tissue necrosis – *Select*	—	Local wound care; medical intervention indicated	Operative debridement or other invasive intervention indicated (e.g., hyperbaric oxygen)	Life-threatening consequences; major invasive intervention indicated (e.g., tissue reconstruction, flap, or grafting)	Death
Trismus (difficulty, restriction or pain when opening mouth)	Trismus	Decreased range of motion without impaired eating	Decreased range of motion requiring small bites, soft foods or purees	Decreased range of motion with inability to adequately aliment or hydrate orally	—	—
NAVIGATION NOTE: Wound-infectious is graded as Infection – *Select* in the INFECTION CATEGORY.						
NAVIGATION NOTE: Wound non-infectious is graded as Wound complication, non-infectious in the DERMATOLOGY/SKIN CATEGORY.						
Musculoskeletal/Soft Tissue – Other (Specify, __)	Musculoskeletal – Other (Specify)	Mild	Moderate	Severe	Life-threatening; disabling	Death

[6] "Assessment of Fracture Risk and its Application to Screening for Postmenopausal Osteoporosis," Report of a *WHO Study Group Technical Report Series*, No. 843, 1994, v + 129 pages [C*, E, F, R, S], ISBN 92 4 120843 0, Sw.fr. 22.-/US $19.80; in developing countries: Sw.fr. 15.40, Order no. 1100843

		Grade				
**Adverse Event**	**Short Name**	**1**	**2**	**3**	**4**	**5**
NAVIGATION NOTE: ADD (Attention Deficit Disorder) is graded as Cognitive disturbance.						
NAVIGATION NOTE: Aphasia, receptive and/or expressive, is graded as Speech impairment (e.g., dysphasia or aphasia).						
Apnea	Apnea	—	—	Present	Intubation indicated	Death
Arachnoiditis/ meningismus/radiculitis	Arachnoiditis	Symptomatic, not interfering with function; medical intervention indicated	Symptomatic (e.g., photophobia, nausea) interfering with function but not interfering with ADL	Symptomatic, interfering with ADL	Life-threatening; disabling (e.g., paraplegia)	Death
ALSO CONSIDER: Fever (in the absence of neutropenia, where neutropenia is defined as ANC <1.0 x 10^9/L); Infection (documented clinically or microbiologically) with Grade 3 or 4 neutrophils (ANC <1.0 x 109/L) – *Select*; Infection with normal ANC or Grade 1 or 2 neutrophils – *Select*; Infection with unknown ANC – *Select*; Pain – *Select*; Vomiting.						
Ataxia (incoordination)	Ataxia	Asymptomatic	Symptomatic, not interfering with ADL	Symptomatic, interfering with ADL; mechanical assistance indicated	Disabling	Death
REMARK: Ataxia (incoordination) refers to the consequence of medical or operative intervention.						
Brachial plexopathy	Brachial plexopathy	Asymptomatic	Symptomatic, not interfering with ADL	Symptomatic, interfering with ADL	Disabling	Death
CNS cerebrovascular ischemia	CNS ischemia	—	Asymptomatic, radiographic findings only	Transient ischemic event or attack (TIA) ≤24 hrs duration	Cerebral vascular accident (CVA, stroke), neurologic deficit >24 hrs	Death
NAVIGATION NOTE: CNS hemorrhage/bleeding is graded as Hemorrhage, CNS in the HEMORRHAGE/BLEEDING CATEGORY.						
CNS necrosis/cystic progression	CNS necrosis	Asymptomatic, radiographic findings only	Symptomatic, not interfering with ADL; medical intervention indicated	Symptomatic and interfering with ADL; hyperbaric oxygen indicated	Life-threatening; disabling; operative intervention indicated to prevent or treat CNS necrosis/cystic progression	Death
Cognitive disturbance	Cognitive disturbance	Mild cognitive disability; not interfering with work/school/life performance; specialized educational services/devices not indicated	Moderate cognitive disability; interfering with work/school/life performance but capable of independent living; specialized resources on part-time basis indicated	Severe cognitive disability; significant impairment of work/school/life performance	Unable to perform ADL; full-time specialized resources or institutionalization indicated	Death
REMARK: Cognitive disturbance may be used for Attention Deficit Disorder (ADD).						

NEUROLOGY

		Grade				
**Adverse Event**	**Short Name**	**1**	**2**	**3**	**4**	**5**
Confusion	Confusion	Transient confusion, disorientation, or attention deficit	Confusion, disorientation, or attention deficit interfering with function, but not interfering with ADL	Confusion or delirium interfering with ADL	Harmful to others or self; hospitalization indicated	Death
Remark: Attention Deficit Disorder (ADD) is graded as Cognitive disturbance.						
Navigation Note: Cranial neuropathy is graded as Neuropathy-cranial – *Select*.						
Dizziness	Dizziness	With head movements or nystagmus only; not interfering with function	Interfering with function, but not interfering with ADL	Interfering with ADL	Disabling	—
Remark: Dizziness includes disequilibrium, lightheadedness, and vertigo.<br>Also Consider: Neuropathy: cranial – *Select*; Syncope (fainting).						
Navigation Note: Dysphasia, receptive and/or expressive, is graded as Speech impairment (e.g., dysphasia or aphasia).						
Encephalopathy	Encephalopathy	—	Mild signs or symptoms; not interfering with ADL	Signs or symptoms interfering with ADL; hospitalization indicated	Life-threatening; disabling	Death
Also Consider: Cognitive disturbance; Confusion; Dizziness; Memory impairment; Mental status; Mood alteration – *Select*; Psychosis (hallucinations/delusions); Somnolence/depressed level of consciousness.						
Extrapyramidal/ involuntary movement/ restlessness	Involuntary movement	Mild involuntary movements not interfering with function	Moderate involuntary movements interfering with function, but not interfering with ADL	Severe involuntary movements or torticollis interfering with ADL	Disabling	Death
Navigation Note: Headache/neuropathic pain (e.g., jaw pain, neurologic pain, phantom limb pain, post-infectious neuralgia, or painful neuropathies) is graded as Pain – *Select* in the PAIN CATEGORY.						
Hydrocephalus	Hydrocephalus	Asymptomatic, radiographic findings only	Mild to moderate symptoms not interfering with ADL	Severe symptoms or neurological deficit interfering with ADL	Disabling	Death
Irritability (children <3 years of age)	Irritability	Mild; easily consolable	Moderate; requiring increased attention	Severe; inconsolable	—	—
Laryngeal nerve dysfunction	Laryngeal nerve	Asymptomatic, weakness on clinical examination/testing only	Symptomatic, but not interfering with ADL; intervention not indicated	Symptomatic, interfering with ADL; intervention indicated (e.g., thyroplasty, vocal cord injection)	Life-threatening; tracheostomy indicated	Death

		Grade				
**Adverse Event**	**Short Name**	**1**	**2**	**3**	**4**	**5**
Leak, cerebrospinal fluid (CSF)	CSF leak	Transient headache; postural care indicated	Symptomatic, not interfering with ADL; blood patch indicated	Symptomatic, interfering with ADL; operative intervention indicated	Life-threatening; disabling	Death
REMARK: Leak, cerebrospinal fluid (CSF) may be used for CSF leak associated with operation and persisting >72 hours.						
Leukoencephalopathy (radiographic findings)	Leukoencephalopathy	Mild increase in subarachnoid space (SAS); mild ventriculomegaly; small (+/- multiple) focal T2 hyperintensities, involving periventricular white matter or <1/3 of susceptible areas of cerebrum	Moderate increase in SAS; moderate ventriculomegaly; focal T2 hyperintensities extending into centrum ovale or involving 1/3 to 2/3 of susceptible areas of cerebrum	Severe increase in SAS; severe ventriculomegaly; near total white matter T2 hyperintensities or diffuse low attenuation (CT)	—	—
REMARK: Leukoencephalopathy is a diffuse white matter process, specifically NOT associated with necrosis. Leukoencephalopathy (radiographic findings) does not include lacunas, which are areas that become void of neural tissue.						
Memory impairment	Memory impairment	Memory impairment not interfering with function	Memory impairment interfering with function, but not interfering with ADL	Memory impairment interfering with ADL	Amnesia	—
Mental status[7]	Mental status	—	1 – 3 point below age and educational norm in Folstein Mini-Mental Status Exam (MMSE)	>3 point below age and educational norm in Folstein MMSE	—	—
Mood alteration – *Select*:<br>– Agitation<br>– Anxiety<br>– Depression<br>– Euphoria	Mood alteration – *Select*	Mild mood alteration not interfering with function	Moderate mood alteration interfering with function, but not interfering with ADL; medication indicated	Severe mood alteration interfering with ADL	Suicidal ideation; danger to self or others	Death
Myelitis	Myelitis	Asymptomatic, mild signs (e.g., Babinski's or Lhermitte's sign)	Weakness or sensory loss not interfering with ADL	Weakness or sensory loss interfering with ADL	Disabling	Death

[7] Folstein MF, Folstein, SE and McHugh PR (1975) "Mini-Mental State: A Practical Method for Grading the State of Patients for the Clinician," *Journal of Psychiatric Research*, 12: 189-198

		Grade				
**Adverse Event**	**Short Name**	**1**	**2**	**3**	**4**	**5**
NAVIGATION NOTE: Neuropathic pain is graded as Pain – *Select* in the PAIN CATEGORY.						
Neuropathy: cranial – *Select*:<br>– CN I Smell<br>– CN II Vision<br>– CN III Pupil, upper eyelid, extra ocular movements<br>– CN IV Downward, inward movement of eye<br>– CN V Motor-jaw muscles; Sensory-facial<br>– CN VI Lateral deviation of eye<br>– CN VII Motor-face; Sensory-taste<br>– CN VIII Hearing and balance<br>– CN IX Motor-pharynx; Sensory-ear, pharynx, tongue<br>– CN X Motor-palate; pharynx, larynx<br>– CN XI Motor-sternomastoid and trapezius<br>– CN XII Motor-tongue	Neuropathy: cranial – *Select*	Asymptomatic, detected on exam/testing only	Symptomatic, not interfering with ADL	Symptomatic, interfering with ADL	Life-threatening; disabling	Death
Neuropathy: motor	Neuropathy-motor	Asymptomatic, weakness on exam/testing only	Symptomatic weakness interfering with function, but not interfering with ADL	Weakness interfering with ADL; bracing or assistance to walk (e.g., cane or walker) indicated	Life-threatening; disabling (e.g., paralysis)	Death
REMARK: Cranial nerve motor neuropathy is graded as Neuropathy: cranial – *Select*.<br>ALSO CONSIDER: Laryngeal nerve dysfunction; Phrenic nerve dysfunction.						
Neuropathy: sensory	Neuropathy-sensory	Asymptomatic; loss of deep tendon reflexes or paresthesia (including tingling) but not interfering with function	Sensory alteration or paresthesia (including tingling), interfering with function, but not interfering with ADL	Sensory alteration or paresthesia interfering with ADL	Disabling	Death
REMARK: Cranial nerve sensory neuropathy is graded as Neuropathy: cranial – *Select*.						
Personality/behavioral	Personality	Change, but not adversely affecting patient or family	Change, adversely affecting patient or family	Mental health intervention indicated	Change harmful to others or self; hospitalization indicated	Death
Phrenic nerve dysfunction	Phrenic nerve	Asymptomatic weakness on exam/testing only	Symptomatic but not interfering with ADL; intervention not indicated	Significant dysfunction; intervention indicated (e.g., diaphragmatic plication)	Life-threatening respiratory compromise; mechanical ventilation indicated	Death
Psychosis (hallucinations/ delusions)	Psychosis	—	Transient episode	Interfering with ADL; medication, supervision	Harmful to others or self; life-threatening	Death

		Grade				
**Adverse Event**	**Short Name**	**1**	**2**	**3**	**4**	**5**
				or restraints indicated	consequences	
Pyramidal tract dysfunction (e.g., ↑ tone, hyperreflexia, positive Babinski, ↓ fine motor coordination)	Pyramidal tract dysfunction	Asymptomatic, abnormality on exam or testing only	Symptomatic; interfering with function but not interfering with ADL	Interfering with ADL	Disabling; paralysis	Death
Seizure	Seizure	—	One brief generalized seizure; seizure(s) well controlled by anticonvulsants or infrequent focal motor seizures not interfering with ADL	Seizures in which consciousness is altered; poorly controlled seizure disorder, with breakthrough generalized seizures despite medical intervention	Seizures of any kind which are prolonged, repetitive, or difficult to control (e.g., status epilepticus, intractable epilepsy)	Death
Somnolence/depressed level of consciousness	Somnolence	—	Somnolence or sedation interfering with function, but not interfering with ADL	Obtundation or stupor; difficult to arouse; interfering with ADL	Coma	Death
Speech impairment (e.g., dysphasia or aphasia)	Speech impairment	—	Awareness of receptive or expressive dysphasia, not impairing ability to communicate	Receptive or expressive dysphasia, impairing ability to communicate	Inability to communicate	—
REMARK: Speech impairment refers to a primary CNS process, not neuropathy or end organ dysfunction.						
ALSO CONSIDER: Laryngeal nerve dysfunction; Voice changes/dysarthria (e.g., hoarseness, loss, or alteration in voice, laryngitis).						
Syncope (fainting)	Syncope (fainting)	—	—	Present	Life-threatening consequences	Death
ALSO CONSIDER: CNS cerebrovascular ischemia; Conduction abnormality/atrioventricular heart block – *Select*; Dizziness; Supraventricular and nodal arrhythmia – *Select*; Vasovagal episode; Ventricular arrhythmia – *Select*.						
NAVIGATION NOTE: Taste alteration (CN VII, IX) is graded as Taste alteration (dysgeusia) in the GASTROINTESTINAL CATEGORY.						
Tremor	Tremor	Mild and brief or intermittent but not interfering with function	Moderate tremor interfering with function, but not interfering with ADL	Severe tremor interfering with ADL	Disabling	—
Neurology – Other (Specify, __)	Neurology – Other (Specify)	Mild	Moderate	Severe	Life-threatening; disabling	Death

OCULAR/VISUAL

Adverse Event	Short Name	Grade 1	Grade 2	Grade 3	Grade 4	Grade 5
Cataract	Cataract	Asymptomatic, detected on exam only	Symptomatic, with moderate decrease in visual acuity (20/40 or better); decreased visual function correctable with glasses	Symptomatic with marked decrease in visual acuity (worse than 20/40); operative intervention indicated (e.g., cataract surgery)	—	—
Dry eye syndrome	Dry eye	Mild, intervention not indicated	Symptomatic, interfering with function but not interfering with ADL; medical intervention indicated	Symptomatic or decrease in visual acuity interfering with ADL; operative intervention indicated	—	—
Eyelid dysfunction	Eyelid dysfunction	Asymptomatic	Symptomatic, interfering with function but not ADL; requiring topical agents or epilation	Symptomatic; interfering with ADL; surgical intervention indicated	—	—
REMARK: Eyelid dysfunction includes canalicular stenosis, ectropion, entropion, erythema, madarosis, symblepharon, telangiectasis, thickening, and trichiasis.						
ALSO CONSIDER: Neuropathy: cranial – *Select*.						
Glaucoma	Glaucoma	Elevated intraocular pressure (EIOP) with single topical agent for intervention; no visual field deficit	EIOP causing early visual field deficit (i.e., nasal step or arcuate deficit); multiple topical or oral agents indicated	EIOP causing marked visual field deficits (i.e., involving both superior and inferior visual fields); operative intervention indicated	EIOP resulting in blindness (20/200 or worse); enucleation indicated	—
Keratitis (corneal inflammation/corneal ulceration)	Keratitis	Abnormal ophthalmologic changes only; intervention not indicated	Symptomatic and interfering with function, but not interfering with ADL	Symptomatic and interfering with ADL; operative intervention indicated	Perforation or blindness (20/200 or worse)	—
NAVIGATION NOTE: Ocular muscle weakness is graded as Muscle weakness, generalized or specific area (not due to neuropathy) – *Select* in the MUSCULOSKELETAL/SOFT TISSUE CATEGORY.						
Night blindness (nyctalopia)	Nyctalopia	Symptomatic, not interfering with function	Symptomatic and interfering with function but not interfering with ADL	Symptomatic and interfering with ADL	Disabling	—

OCULAR/VISUAL

		Grade				
**Adverse Event**	**Short Name**	**1**	**2**	**3**	**4**	**5**
Nystagmus	Nystagmus	Asymptomatic	Symptomatic and interfering with function but not interfering with ADL	Symptomatic and interfering with ADL	Disabling	—
ALSO CONSIDER: Neuropathy: cranial – *Select*; Ophthalmoplegia/diplopia (double vision).						
Ocular surface disease	Ocular surface disease	Asymptomatic or minimally symptomatic but not interfering with function	Symptomatic, interfering with function but not interfering with ADL; topical antibiotics or other topical intervention indicated	Symptomatic, interfering with ADL; operative intervention indicated	—	—
REMARK: Ocular surface disease includes conjunctivitis, keratoconjunctivitis sicca, chemosis, keratinization, and palpebral conjunctival epithelial metaplasia.						
Ophthalmoplegia/ diplopia (double vision)	Diplopia	Intermittently symptomatic, intervention not indicated	Symptomatic and interfering with function but not interfering with ADL	Symptomatic and interfering with ADL; surgical intervention indicated	Disabling	—
ALSO CONSIDER: Neuropathy: cranial – *Select*.						
Optic disc edema	Optic disc edema	Asymptomatic	Decreased visual acuity (20/40 or better); visual field defect present	Decreased visual acuity (worse than 20/40); marked visual field defect but sparing the central 20 degrees	Blindness (20/200 or worse)	—
ALSO CONSIDER: Neuropathy: cranial – *Select*.						
Proptosis/enophthalmos	Proptosis/enophthalmos	Asymptomatic, intervention not indicated	Symptomatic and interfering with function, but not interfering with ADL	Symptomatic and interfering with ADL	—	—
Retinal detachment	Retinal detachment	Exudative; no central vision loss; intervention not indicated	Exudative and visual acuity 20/40 or better but intervention not indicated	Rhegmatogenous or exudative detachment; operative intervention indicated	Blindness (20/200 or worse)	—
Retinopathy	Retinopathy	Asymptomatic	Symptomatic with moderate decrease in visual acuity (20/40 or better)	Symptomatic with marked decrease in visual acuity (worse than 20/40)	Blindness (20/200 or worse)	—

OCULAR/VISUAL

		Grade				
**Adverse Event**	**Short Name**	**1**	**2**	**3**	**4**	**5**
Scleral necrosis/melt	Scleral necrosis	Asymptomatic or symptomatic but not interfering with function	Symptomatic, interfering with function but not interfering with ADL; moderate decrease in visual acuity (20/40 or better); medical intervention indicated	Symptomatic, interfering with ADL; marked decrease in visual acuity (worse than 20/40); operative intervention indicated	Blindness (20/200 or worse); painful eye with enucleation indicated	—
Uveitis	Uveitis	Asymptomatic	Anterior uveitis; medical intervention indicated	Posterior or pan-uveitis; operative intervention indicated	Blindness (20/200 or worse)	—
Vision-blurred vision	Blurred vision	Symptomatic not interfering with function	Symptomatic and interfering with function, but not interfering with ADL	Symptomatic and interfering with ADL	Disabling	—
Vision-flashing lights/floaters	Flashing lights	Symptomatic not interfering with function	Symptomatic and interfering with function, but not interfering with ADL	Symptomatic and interfering with ADL	Disabling	—
Vision-photophobia	Photophobia	Symptomatic not interfering with function	Symptomatic and interfering with function, but not interfering with ADL	Symptomatic and interfering with ADL	Disabling	—
Vitreous hemorrhage	Vitreous hemorrhage	Asymptomatic, clinical findings only	Symptomatic, interfering with function, but not interfering with ADL; intervention not indicated	Symptomatic, interfering with ADL; vitrectomy indicated	—	—
Watery eye (epiphora, tearing)	Watery eye	Symptomatic, intervention not indicated	Symptomatic, interfering with function but not interfering with ADL	Symptomatic, interfering with ADL	—	—
Ocular/Visual – Other (Specify, __)	Ocular – Other (Specify)	Symptomatic not interfering with function	Symptomatic and interfering with function, but not interfering with ADL	Symptomatic and interfering with ADL	Blindness (20/200 or worse)	Death

PAIN

		Grade				
**Adverse Event**	**Short Name**	**1**	**2**	**3**	**4**	**5**
Pain – *Select*: ‘*Select*’ AEs appear at the end of the CATEGORY.	Pain – *Select*	Mild pain not interfering with function	Moderate pain; pain or analgesics interfering with function, but not interfering with ADL	Severe pain; pain or analgesics severely interfering with ADL	Disabling	—
Pain – Other (Specify, __)	Pain – Other (Specify)	Mild pain not interfering with function	Moderate pain; pain or analgesics interfering with function, but not interfering with ADL	Severe pain; pain or analgesics severely interfering with ADL	Disabling	—

PAIN – *SELECT*

AUDITORY/EAR
- External ear
- Middle ear

CARDIOVASCULAR
- Cardiac/heart
- Pericardium

DERMATOLOGY/SKIN
- Face
- Lip
- Oral-gums
- Scalp
- Skin

GASTROINTESTINAL
- Abdomen NOS
- Anus
- Dental/teeth/peridontal
- Esophagus
- Oral cavity
- Peritoneum
- Rectum
- Stomach

GENERAL
- Pain NOS
- Tumor pain

HEPATOBILIARY/PANCREAS
- Gallbladder
- Liver

LYMPHATIC
- Lymph node

MUSCULOSKELETAL
- Back
- Bone
- Buttock
- Extremity-limb
- Intestine
- Joint
- Muscle
- Neck
- Phantom (pain associated with missing limb)

NEUROLOGY
- Head/headache
- Neuralgia/peripheral nerve

OCULAR
- Eye

PULMONARY/UPPER RESPIRATORY
- Chest wall
- Chest/thorax NOS

PULMONARY/UPPER RESPIRATORY (*continued*)
- Larynx
- Pleura
- Sinus
- Throat/pharynx/larynx

RENAL/GENITOURINARY
- Bladder
- Kidney

SEXUAL/REPRODUCTIVE FUNCTION
- Breast
- Ovulatory
- Pelvis
- Penis
- Perineum
- Prostate
- Scrotum
- Testicle
- Urethra
- Uterus
- Vagina

PULMONARY/UPPER RESPIRATORY

		Grade				
**Adverse Event**	**Short Name**	**1**	**2**	**3**	**4**	**5**
Adult Respiratory Distress Syndrome (ARDS)	ARDS	—	—	Present, intubation not indicated	Present, intubation indicated	Death
ALSO CONSIDER: Dyspnea (shortness of breath); Hypoxia; Pneumonitis/pulmonary infiltrates.						
Aspiration	Aspiration	Asymptomatic ("silent aspiration"); endoscopy or radiographic (e.g., barium swallow) findings	Symptomatic (e.g., altered eating habits, coughing or choking episodes consistent with aspiration); medical intervention indicated (e.g., antibiotics, suction or oxygen)	Clinical or radiographic signs of pneumonia or pneumonitis; unable to aliment orally	Life-threatening (e.g., aspiration pneumonia or pneumonitis)	Death
ALSO CONSIDER: Infection (documented clinically or microbiologically) with Grade 3 or 4 neutrophils (ANC <1.0 x 10^9/L) – *Select;* Infection with normal ANC or Grade 1 or 2 neutrophils – *Select;* Infection with unknown ANC – *Select;* Laryngeal nerve dysfunction; Neuropathy: cranial – *Select*; Pneumonitis/pulmonary infiltrates.						
Atelectasis	Atelectasis	Asymptomatic	Symptomatic (e.g., dyspnea, cough), medical intervention indicated (e.g., bronchoscopic suctioning, chest physiotherapy, suctioning)	Operative (e.g., stent, laser) intervention indicated	Life-threatening respiratory compromise	Death
ALSO CONSIDER: Adult Respiratory Distress Syndrome (ARDS); Cough; Dyspnea (shortness of breath); Hypoxia; Infection (documented clinically or microbiologically) with Grade 3 or 4 neutrophils (ANC <1.0 x 109/L) – *Select*; Infection with normal ANC or Grade 1 or 2 neutrophils – *Select*; Infection with unknown ANC – *Select*; Obstruction/stenosis of airway – *Select*; Pneumonitis/pulmonary infiltrates; Pulmonary fibrosis (radiographic changes).						
Bronchospasm, wheezing	Bronchospasm	Asymptomatic	Symptomatic not interfering with function	Symptomatic interfering with function	Life-threatening	Death
ALSO CONSIDER: Allergic reaction/hypersensitivity (including drug fever); Dyspnea (shortness of breath).						
Carbon monoxide diffusion capacity (DL_{CO})	DL_{CO}	90 – 75% of predicted value	<75 – 50% of predicted value	<50 – 25% of predicted value	<25% of predicted value	Death
ALSO CONSIDER: Hypoxia; Pneumonitis/pulmonary infiltrates; Pulmonary fibrosis (radiographic changes).						
Chylothorax	Chylothorax	Asymptomatic	Symptomatic; thoracentesis or tube drainage indicated	Operative intervention indicated	Life-threatening (e.g., hemodynamic instability or ventilatory support indicated)	Death
Cough	Cough	Symptomatic, non-narcotic medication only indicated	Symptomatic and narcotic medication indicated	Symptomatic and significantly interfering with sleep or ADL	—	—

PULMONARY/UPPER RESPIRATORY

		Grade				
**Adverse Event**	**Short Name**	**1**	**2**	**3**	**4**	**5**
Dyspnea (shortness of breath)	Dyspnea	Dyspnea on exertion, but can walk 1 flight of stairs without stopping	Dyspnea on exertion but unable to walk 1 flight of stairs or 1 city block (0.1km) without stopping	Dyspnea with ADL	Dyspnea at rest; intubation/ventilator indicated	Death
ALSO CONSIDER: Hypoxia; Neuropathy: motor; Pneumonitis/pulmonary infiltrates; Pulmonary fibrosis (radiographic changes).						
Edema, larynx	Edema, larynx	Asymptomatic edema by exam only	Symptomatic edema, no respiratory distress	Stridor; respiratory distress; interfering with ADL	Life-threatening airway compromise; tracheotomy, intubation, or laryngectomy indicated	Death
ALSO CONSIDER: Allergic reaction/hypersensitivity (including drug fever).						
FEV_1	FEV_1	90 – 75% of predicted value	<75 – 50% of predicted value	<50 – 25% of predicted value	<25% of predicted	Death
Fistula, pulmonary/upper respiratory<br>– *Select*:<br>– Bronchus<br>– Larynx<br>– Lung<br>– Oral cavity<br>– Pharynx<br>– Pleura<br>– Trachea	Fistula, pulmonary<br>– *Select*	Asymptomatic, radiographic findings only	Symptomatic, tube thoracostomy or medical management indicated; associated with altered respiratory function but not interfering with ADL	Symptomatic and associated with altered respiratory function interfering with ADL; or endoscopic (e.g., stent) or primary closure by operative intervention indicated	Life-threatening consequences; operative intervention with thoracoplasty, chronic open drainage or multiple thoracotomies indicated	Death
REMARK: A fistula is defined as an abnormal communication between two body cavities, potential spaces, and/or the skin. The site indicated for a fistula should be the site from which the abnormal process is believed to have arisen. For example, a tracheo-esophageal fistula arising in the context of a resected or irradiated esophageal cancer should be graded as Fistula, GI – esophagus in the GASTROINTESTINAL CATEGORY.						
NAVIGATION NOTE: Hemoptysis is graded as Hemorrhage, pulmonary/upper respiratory – *Select* in the HEMORRHAGE/BLEEDING CATEGORY.						
Hiccoughs (hiccups, singultus)	Hiccoughs	Symptomatic, intervention not indicated	Symptomatic, intervention indicated	Symptomatic, significantly interfering with sleep or ADL	—	—
Hypoxia	Hypoxia	—	Decreased O_2 saturation with exercise (e.g., pulse oximeter <88%); intermittent supplemental oxygen	Decreased O_2 saturation at rest; continuous oxygen indicated	Life-threatening; intubation or ventilation indicated	Death

		Grade				
**Adverse Event**	**Short Name**	**1**	**2**	**3**	**4**	**5**
Nasal cavity/paranasal sinus reactions	Nasal/paranasal reactions	Asymptomatic mucosal crusting, blood-tinged secretions	Symptomatic stenosis or edema/narrowing interfering with airflow	Stenosis with significant nasal obstruction; interfering with ADL	Necrosis of soft tissue or bone	Death
ALSO CONSIDER: Infection (documented clinically or microbiologically) with Grade 3 or 4 neutrophils (ANC <1.0 x 10^9/L) – *Select;* Infection with normal ANC or Grade 1 or 2 neutrophils – *Select;* Infection with unknown ANC – *Select*.						
Obstruction/stenosis of airway – *Select*: – Bronchus – Larynx – Pharynx – Trachea	Airway obstruction – *Select*	Asymptomatic obstruction or stenosis on exam, endoscopy, or radiograph	Symptomatic (e.g., noisy airway breathing), but causing no respiratory distress; medical management indicated (e.g., steroids)	Interfering with ADL; stridor or endoscopic intervention indicated (e.g., stent, laser)	Life-threatening airway compromise; tracheotomy or intubation indicated	Death
Pleural effusion (non-malignant)	Pleural effusion	Asymptomatic	Symptomatic, intervention such as diuretics or up to 2 therapeutic thoracenteses indicated	Symptomatic and supplemental oxygen, >2 therapeutic thoracenteses, tube drainage, or pleurodesis indicated	Life-threatening (e.g., causing hemodynamic instability or ventilatory support indicated)	Death
ALSO CONSIDER: Atelectasis; Cough; Dyspnea (shortness of breath); Hypoxia; Pneumonitis/pulmonary infiltrates; Pulmonary fibrosis (radiographic changes).						
NAVIGATION NOTE: Pleuritic pain is graded as Pain – *Select* in the PAIN CATEGORY.						
Pneumonitis/pulmonary infiltrates	Pneumonitis	Asymptomatic, radiographic findings only	Symptomatic, not interfering with ADL	Symptomatic, interfering with ADL; O_2 indicated	Life-threatening; ventilatory support indicated	Death
ALSO CONSIDER: Adult Respiratory Distress Syndrome (ARDS); Cough; Dyspnea (shortness of breath); Hypoxia; Infection (documented clinically or microbiologically) with Grade 3 or 4 neutrophils (ANC <1.0 x 10^9/L) – *Select;* Infection with normal ANC or Grade 1 or 2 neutrophils – *Select;* Infection with unknown ANC – *Select*; Pneumonitis/pulmonary infiltrates; Pulmonary fibrosis (radiographic changes).						
Pneumothorax	Pneumothorax	Asymptomatic, radiographic findings only	Symptomatic; intervention indicated (e.g., hospitalization for observation, tube placement without sclerosis)	Sclerosis and/or operative intervention indicated	Life-threatening, causing hemodynamic instability (e.g., tension pneumothorax); ventilatory support indicated	Death
Prolonged chest tube drainage or air leak after pulmonary resection	Chest tube drainage or leak	—	Sclerosis or additional tube thoracostomy indicated	Operative intervention indicated (e.g., thoracotomy with stapling or sealant application)	Life-threatening; debilitating; organ resection indicated	Death

		Grade				
**Adverse Event**	**Short Name**	**1**	**2**	**3**	**4**	**5**
Prolonged intubation after pulmonary resection (>24 hrs after surgery)	Prolonged intubation	—	Extubated within 24 – 72 hrs postoperatively	Extubated >72 hrs postoperatively, but before tracheostomy indicated	Tracheostomy indicated	Death
NAVIGATION NOTE: Pulmonary embolism is graded as Grade 4 either as Thrombosis/embolism (vascular access-related) or Thrombosis/thrombus/embolism in the VASCULAR CATEGORY.						
Pulmonary fibrosis (radiographic changes)	Pulmonary fibrosis	Minimal radiographic findings (or patchy or bi-basilar changes) with estimated radiographic proportion of total lung volume that is fibrotic of <25%	Patchy or bi-basilar changes with estimated radiographic proportion of total lung volume that is fibrotic of 25 – <50%	Dense or widespread infiltrates/consolidation with estimated radiographic proportion of total lung volume that is fibrotic of 50 – <75%	Estimated radiographic proportion of total lung volume that is fibrotic is ≥75%; honeycombing	Death
REMARK: Fibrosis is usually a "late effect" seen >3 months after radiation or combined modality therapy (including surgery). It is thought to represent scar/fibrotic lung tissue. It may be difficult to distinguish from pneumonitis that is generally seen within 3 months of radiation or combined modality therapy.						
ALSO CONSIDER: Adult Respiratory Distress Syndrome (ARDS); Cough; Dyspnea (shortness of breath); Hypoxia; Infection (documented clinically or microbiologically) with Grade 3 or 4 neutrophils (ANC <1.0 x 10^9/L) – *Select;* Infection with normal ANC or Grade 1 or 2 neutrophils – *Select;* Infection with unknown ANC – *Select.*						
NAVIGATION NOTE: Recurrent laryngeal nerve dysfunction is graded as Laryngeal nerve dysfunction in the NEUROLOGY CATEGORY.						
Vital capacity	Vital capacity	90 – 75% of predicted value	<75 – 50% of predicted value	<50 – 25% of predicted value	<25% of predicted value	Death
Voice changes/dysarthria (e.g., hoarseness, loss or alteration in voice, laryngitis)	Voice changes	Mild or intermittent hoarseness or voice change, but fully understandable	Moderate or persistent voice changes, may require occasional repetition but understandable on telephone	Severe voice changes including predominantly whispered speech; may require frequent repetition or face-to-face contact for understandability; requires voice aid (e.g., electrolarynx) for ≤50% of communication	Disabling; non-understandable voice or aphonic; requires voice aid (e.g., electrolarynx) for >50% of communication or requires >50% written communication	Death
ALSO CONSIDER: Laryngeal nerve dysfunction; Speech impairment (e.g., dysphasia or aphasia).						
Pulmonary/Upper Respiratory – Other (Specify, __)	Pulmonary – Other (Specify)	Mild	Moderate	Severe	Life-threatening; disabling	Death

		Grade				
**Adverse Event**	**Short Name**	**1**	**2**	**3**	**4**	**5**
Bladder spasms	Bladder spasms	Symptomatic, intervention not indicated	Symptomatic, antispasmodics indicated	Narcotics indicated	Major surgical intervention indicated (e.g., cystectomy)	—
Cystitis	Cystitis	Asymptomatic	Frequency with dysuria; macroscopic hematuria	Transfusion; IV pain medications; bladder irrigation indicated	Catastrophic bleeding; major non-elective intervention indicated	Death
ALSO CONSIDER: Infection (documented clinically or microbiologically) with Grade 3 or 4 neutrophils (ANC <1.0 x 109/L) – *Select*; Infection with normal ANC or Grade 1 or 2 neutrophils – *Select*; Infection with unknown ANC – *Select*; Pain – *Select*.						
Fistula, GU – *Select*:<br>– Bladder<br>– Genital tract-female<br>– Kidney<br>– Ureter<br>– Urethra<br>– Uterus<br>– Vagina	Fistula, GU – *Select*	Asymptomatic, radiographic findings only	Symptomatic; noninvasive intervention indicated	Symptomatic interfering with ADL; invasive intervention indicated	Life-threatening consequences; operative intervention requiring partial or full organ resection; permanent urinary diversion	Death
REMARK: A fistula is defined as an abnormal communication between two body cavities, potential spaces, and/or the skin. The site indicated for a fistula should be the site from which the abnormal process is believed to have originated.						
Incontinence, urinary	Incontinence, urinary	Occasional (e.g., with coughing, sneezing, etc.), pads not indicated	Spontaneous, pads indicated	Interfering with ADL; intervention indicated (e.g., clamp, collagen injections)	Operative intervention indicated (e.g., cystectomy or permanent urinary diversion)	—
Leak (including anastomotic), GU – *Select*:<br>– Bladder<br>– Fallopian tube<br>– Kidney<br>– Spermatic cord<br>– Stoma<br>– Ureter<br>– Urethra<br>– Uterus<br>– Vagina<br>– Vas deferens	Leak, GU – *Select*	Asymptomatic, radiographic findings only	Symptomatic; medical intervention indicated	Symptomatic, interfering with GU function; invasive or endoscopic intervention indicated	Life-threatening	Death
REMARK: Leak (including anastomotic), GU – *Select* refers to clinical signs and symptoms or radiographic confirmation of anastomotic leak but without development of fistula.						

		Grade				
**Adverse Event**	**Short Name**	**1**	**2**	**3**	**4**	**5**
Obstruction, GU – *Select*:<br>– Bladder<br>– Fallopian tube<br>– Prostate<br>– Spermatic cord<br>– Stoma<br>– Testes<br>– Ureter<br>– Urethra<br>– Uterus<br>– Vagina<br>– Vas deferens	Obstruction, GU – *Select*	Asymptomatic, radiographic or endoscopic findings only	Symptomatic but no hydronephrosis, sepsis or renal dysfunction; dilation or endoscopic repair or stent placement indicated	Symptomatic and altered organ function (e.g., sepsis or hydronephrosis, or renal dysfunction); operative intervention indicated	Life-threatening consequences; organ failure or operative intervention requiring complete organ resection indicated	Death
NAVIGATION NOTE: Operative injury is graded as Intra-operative injury – *Select Organ or Structure* in the SURGERY/INTRA-OPERATIVE INJURY CATEGORY.						
Perforation, GU – *Select*:<br>– Bladder<br>– Fallopian tube<br>– Kidney<br>– Ovary<br>– Prostate<br>– Spermatic cord<br>– Stoma<br>– Testes<br>– Ureter<br>– Urethra<br>– Uterus<br>– Vagina<br>– Vas deferens	Perforation, GU – *Select*	Asymptomatic radiographic findings only	Symptomatic, associated with altered renal/GU function	Symptomatic, operative intervention indicated	Life-threatening consequences or organ failure; operative intervention requiring organ resection indicated	Death
Prolapse of stoma, GU	Prolapse stoma, GU	Asymptomatic; special intervention, extraordinary care not indicated	Extraordinary local care or maintenance; minor revision under local anesthesia indicated	Dysfunctional stoma; operative intervention or major stomal revision indicated	Life-threatening consequences	Death
REMARK: Other stoma complications may be graded as Fistula, GU – *Select*; Leak (including anastomotic), GU – *Select*; Obstruction, GU – *Select;* Perforation, GU – *Select*; Stricture/stenosis (including anastomotic), GU – *Select*.						
Renal failure	Renal failure	—	—	Chronic dialysis not indicated	Chronic dialysis or renal transplant indicated	Death
ALSO CONSIDER: Glomerular filtration rate.						

RENAL/GENITOURINARY

		Grade				
**Adverse Event**	**Short Name**	**1**	**2**	**3**	**4**	**5**
Stricture/stenosis (including anastomotic), GU – *Select*:<br>– Bladder<br>– Fallopian tube<br>– Prostate<br>– Spermatic cord<br>– Stoma<br>– Testes<br>– Ureter<br>– Urethra<br>– Uterus<br>– Vagina<br>– Vas deferens	Stricture, anastomotic, GU – *Select*	Asymptomatic, radiographic or endoscopic findings only	Symptomatic but no hydronephrosis, sepsis or renal dysfunction; dilation or endoscopic repair or stent placement indicated	Symptomatic and altered organ function (e.g., sepsis or hydronephrosis, or renal dysfunction); operative intervention indicated	Life-threatening consequences; organ failure or operative intervention requiring organ resection indicated	Death
ALSO CONSIDER: Obstruction, GU – *Select*.						
Urinary electrolyte wasting (e.g., Fanconi's syndrome, renal tubular acidosis)	Urinary electrolyte wasting	Asymptomatic, intervention not indicated	Mild, reversible and manageable with replacement	Irreversible, requiring continued replacement	—	—
ALSO CONSIDER: Acidosis (metabolic or respiratory); Bicarbonate, serum-low; Calcium, serum-low (hypocalcemia); Phosphate, serum-low (hypophosphatemia).						
Urinary frequency/urgency	Urinary frequency	Increase in frequency or nocturia up to 2 x normal; enuresis	Increase >2 x normal but <hourly	≥1 x/hr; urgency; catheter indicated	—	—
Urinary retention (including neurogenic bladder)	Urinary retention	Hesitancy or dribbling, no significant residual urine; retention occurring during the immediate postoperative period	Hesitancy requiring medication; or operative bladder atony requiring indwelling catheter beyond immediate postoperative period but for <6 weeks	More than daily catheterization indicated; urological intervention indicated (e.g., TURP, suprapubic tube, urethrotomy)	Life-threatening consequences; organ failure (e.g., bladder rupture); operative intervention requiring organ resection indicated	Death
REMARK: The etiology of retention (if known) is graded as Obstruction, GU – *Select*; Stricture/stenosis (including anastomotic), GU – *Select*.<br>ALSO CONSIDER: Obstruction, GU – *Select*; Stricture/stenosis (including anastomotic), GU – *Select*.						
Urine color change	Urine color change	Present	—	—	—	—
REMARK: Urine color refers to change that is not related to other dietary or physiologic cause (e.g., bilirubin, concentrated urine, and hematuria).						
Renal/Genitourinary – Other (Specify, __)	Renal – Other (Specify)	Mild	Moderate	Severe	Life-threatening; disabling	Death

SECONDARY MALIGNANCY

		Grade				
**Adverse Event**	**Short Name**	**1**	**2**	**3**	**4**	**5**
Secondary Malignancy – possibly related to cancer treatment (Specify, __)	Secondary Malignancy (possibly related to cancer treatment)	—	—	Non-life-threatening basal or squamous cell carcinoma of the skin	Solid tumor, leukemia or lymphoma	Death

REMARK: Secondary malignancy excludes metastasis from initial primary. Any malignancy possibly related to cancer treatment (including AML/MDS) should be reported via the routine reporting mechanisms outlined in each protocol. Important: Secondary Malignancy is an exception to NCI Expedited Adverse Event Reporting Guidelines. Secondary Malignancy is "Grade 4, present" but NCI does not require AdEERS Expedited Reporting for any (related or unrelated to treatment) Secondary Malignancy. A diagnosis of AML/MDS following treatment with an NCI-sponsored investigational agent is to be reported using the form available from the CTEP Web site at http://ctep.cancer.gov. Cancers not suspected of being treatment-related are not to be reported here.

SEXUAL/REPRODUCTIVE FUNCTION

		Grade				
**Adverse Event**	**Short Name**	**1**	**2**	**3**	**4**	**5**
Breast function/lactation	Breast function	Mammary abnormality, not functionally significant	Mammary abnormality, functionally significant	—	—	—
Breast nipple/areolar deformity	Nipple/areolar	Limited areolar asymmetry with no change in nipple/areolar projection	Asymmetry of nipple areolar complex with slight deviation in nipple projection	Marked deviation of nipple projection	—	—
Breast volume/hypoplasia	Breast	Minimal asymmetry; minimal hypoplasia	Asymmetry exists, ≤1/3 of the breast volume; moderate hypoplasia	Asymmetry exists, >1/3 of the breast volume; severe hypoplasia	—	—
REMARK: Breast volume is referenced with both arms straight overhead.						
NAVIGATION NOTE: Dysmenorrhea is graded as Pain – *Select* in the PAIN CATEGORY.						
NAVIGATION NOTE: Dyspareunia is graded as Pain – *Select* in the PAIN CATEGORY.						
NAVIGATION NOTE: Dysuria (painful urination) is graded as Pain – *Select* in the PAIN CATEGORY.						
Erectile dysfunction	Erectile dysfunction	Decrease in erectile function (frequency/rigidity of erections) but erectile aids not indicated	Decrease in erectile function (frequency/rigidity of erections), erectile aids indicated	Decrease in erectile function (frequency/rigidity of erections) but erectile aids not helpful; penile prosthesis indicated	—	—
Ejaculatory dysfunction	Ejaculatory dysfunction	Diminished ejaculation	Anejaculation or retrograde ejaculation	—	—	—
NAVIGATION NOTE: Feminization of male is graded in the ENDOCRINE CATEGORY.						
Gynecomastia	Gynecomastia	—	Asymptomatic breast enlargement	Symptomatic breast enlargement; intervention indicated	—	—
ALSO CONSIDER: Pain – *Select*.						
Infertility/sterility	Infertility/sterility	—	Male: oligospermia/low sperm count<br>Female: diminished fertility/ovulation	Male: sterile/azoospermia<br>Female: infertile/ anovulatory	—	—
Irregular menses (change from baseline)	Irregular menses	1 – 3 months without menses	>3 – 6 months without menses but continuing menstrual cycles	Persistent amenorrhea for >6 months	—	—

SEXUAL/REPRODUCTIVE FUNCTION

		Grade				
**Adverse Event**	**Short Name**	**1**	**2**	**3**	**4**	**5**
Libido	Libido	Decrease in interest but not affecting relationship; intervention not indicated	Decrease in interest and adversely affecting relationship; intervention indicated	—	—	—
NAVIGATION NOTE: Masculinization of female is graded in the ENDOCRINE CATEGORY.						
Orgasmic dysfunction	Orgasmic function	Transient decrease	Decrease in orgasmic response requiring intervention	Complete inability of orgasmic response; not responding to intervention	—	—
NAVIGATION NOTE: Pelvic pain is graded as Pain – *Select* in the PAIN CATEGORY.						
NAVIGATION NOTE: Ulcers of the labia or perineum are graded as Ulceration in DERMATOLOGY/SKIN CATEGORY.						
Vaginal discharge (non-infectious)	Vaginal discharge	Mild	Moderate to heavy; pad use indicated	—	—	—
Vaginal dryness	Vaginal dryness	Mild	Interfering with sexual function; dyspareunia; intervention indicated	—	—	—
ALSO CONSIDER: Pain – *Select*.						
Vaginal mucositis	Vaginal mucositis	Erythema of the mucosa; minimal symptoms	Patchy ulcerations; moderate symptoms or dyspareunia	Confluent ulcerations; bleeding with trauma; unable to tolerate vaginal exam, sexual intercourse or tampon placement	Tissue necrosis; significant spontaneous bleeding; life-threatening consequences	—
Vaginal stenosis/length	Vaginal stenosis	Vaginal narrowing and/or shortening not interfering with function	Vaginal narrowing and/or shortening interfering with function	Complete obliteration; not surgically correctable	—	—
Vaginitis (not due to infection)	Vaginitis	Mild, intervention not indicated	Moderate, intervention indicated	Severe, not relieved with treatment; ulceration, but operative intervention not indicated	Ulceration and operative intervention indicated	—
Sexual/Reproductive Function – Other (Specify, __)	Sexual – Other (Specify)	Mild	Moderate	Severe	Disabling	Death

SURGERY/INTRA-OPERATIVE INJURY

		Grade				
**Adverse Event**	**Short Name**	**1**	**2**	**3**	**4**	**5**
NAVIGATION NOTE: Intra-operative hemorrhage is graded as Hemorrhage/bleeding associated with surgery, intra-operative or postoperative in the HEMORRHAGE/BLEEDING CATEGORY.						
Intra-operative injury – *Select Organ or Structure*<br>*'Select'* AEs appear at the end of the CATEGORY.	Intraop injury – *Select*	Primary repair of injured organ/structure indicated	Partial resection of injured organ/structure indicated	Complete resection or reconstruction of injured organ/structure indicated	Life threatening consequences; disabling	—
REMARK: The *'Select'* AEs are defined as significant, unanticipated injuries that are recognized at the time of surgery. These AEs do not refer to additional surgical procedures that must be performed because of a change in the operative plan based on intra-operative findings. Any sequelae resulting from the intra-operative injury that result in an adverse outcome for the patient must also be recorded and graded under the relevant CTCAE Term.						
Intra-operative Injury – Other (Specify, __)	Intraop Injury – Other (Specify)	Primary repair of injured organ/structure indicated	Partial resection of injured organ/structure indicated	Complete resection or reconstruction of injured organ/structure indicated	Life threatening consequences; disabling	—
REMARK: Intra-operative Injury – Other (Specify, __) is to be used only to report an organ/structure not included in the *'Select'* AEs found at the end of the CATEGORY. Any sequelae resulting from the intra-operative injury that result in an adverse outcome for the patient must also be recorded and graded under the relevant CTCAE Term.						

AUDITORY/EAR
- Inner ear
- Middle ear
- Outer ear NOS
- Outer ear-Pinna

CARDIOVASCULAR
- Artery-aorta
- Artery-carotid
- Artery-cerebral
- Artery-extremity (lower)
- Artery-extremity (upper)
- Artery-hepatic
- Artery-major visceral artery
- Artery-pulmonary
- Artery NOS
- Heart
- Spleen
- Vein-extremity (lower)
- Vein-extremity (upper)
- Vein-hepatic
- Vein-inferior vena cava
- Vein-jugular
- Vein-major visceral vein
- Vein-portal vein
- Vein-pulmonary
- Vein-superior vena cava
- Vein NOS

DERMATOLOGY/SKIN
- Breast
- Nails
- Skin

ENDOCRINE
- Adrenal gland
- Parathyroid
- Pituitary

ENDOCRINE (*continued*)
- Thyroid

HEAD AND NECK
- Gingiva
- Larynx
- Lip/perioral area
- Face NOS
- Nasal cavity
- Nasopharynx
- Neck NOS
- Nose
- Oral cavity NOS
- Parotid gland
- Pharynx
- Salivary duct
- Salivary gland
- Sinus
- Teeth
- Tongue
- Upper aerodigestive NOS

GASTROINTESTINAL
- Abdomen NOS
- Anal sphincter
- Anus
- Appendix
- Cecum
- Colon
- Duodenum
- Esophagus
- Ileum
- Jejunum
- Oral
- Peritoneal cavity
- Rectum
- Small bowel NOS

GASTROINTESTINAL (*continued*)
- Stoma (GI)
- Stomach

HEPATOBILIARY/ PANCREAS
- Biliary tree-common bile duct
- Biliary tree-common hepatic duct
- Biliary tree-left hepatic duct
- Biliary tree-right hepatic duct
- Biliary tree NOS
- Gallbladder
- Liver
- Pancreas
- Pancreatic duct

MUSCULOSKELETAL
- Bone
- Cartilage
- Extremity-lower
- Extremity-upper
- Joint
- Ligament
- Muscle
- Soft tissue NOS
- Tendon

NEUROLOGY
- Brain
- Meninges
- Spinal cord

<u>NERVES:</u>
- Brachial plexus
- CN I (olfactory)
- CN II (optic)
- CN III (oculomotor)
- CN IV (trochlear)

NEUROLOGY (*continued*)

<u>NERVES:</u>
- CN V (trigeminal) motor
- CN V (trigeminal) sensory
- CN VI (abducens)
- CN VII (facial) motor-face
- CN VII (facial) sensory-taste
- CN VIII (vestibulocochlear)
- CN IX (glossopharyngeal) motor pharynx
- CN IX (glossopharyngeal) sensory ear-pharynx-tongue
- CN X (vagus)
- CN XI (spinal accessory)
- CN XII (hypoglossal)
- Cranial nerve or branch NOS
- Lingual
- Lung thoracic
- Peripheral motor NOS
- Peripheral sensory NOS
- Recurrent laryngeal
- Sacral plexus
- Sciatic
- Thoracodorsal

OCULAR
- Conjunctiva
- Cornea
- Eye NOS
- Lens
- Retina

PULMONARY/UPPER RESPIRATORY
- Bronchus
- Lung
- Mediastinum
- Pleura
- Thoracic duct
- Trachea
- Upper airway NOS

RENAL/GENITOURINARY
- Bladder
- Cervix
- Fallopian tube
- Kidney
- Ovary
- Pelvis NOS
- Penis
- Prostate
- Scrotum
- Testis
- Ureter
- Urethra
- Urinary conduit
- Urinary tract NOS
- Uterus
- Vagina
- Vulva

SYNDROMES

		Grade				
**Adverse Event**	**Short Name**	**1**	**2**	**3**	**4**	**5**
NAVIGATION NOTE: Acute vascular leak syndrome is graded in the VASCULAR CATEGORY.						
NAVIGATION NOTE: Adrenal insufficiency is graded in the ENDOCRINE CATEGORY.						
NAVIGATION NOTE: Adult Respiratory Distress Syndrome (ARDS) is graded in the PULMONARY/UPPER RESPIRATORY CATEGORY.						
Alcohol intolerance syndrome (antabuse-like syndrome)	Alcohol intolerance syndrome	—	—	Present	—	Death
REMARK: An antabuse-like syndrome occurs with some new anti-androgens (e.g., nilutamide) when patient also consumes alcohol.						
NAVIGATION NOTE: Autoimmune reaction is graded as Autoimmune reaction/hypersensitivity (including drug fever) in the ALLERGY/IMMUNOLOGY CATEGORY.						
Cytokine release syndrome/acute infusion reaction	Cytokine release syndrome	Mild reaction; infusion interruption not indicated; intervention not indicated	Requires therapy or infusion interruption but responds promptly to symptomatic treatment (e.g., antihistamines, NSAIDS, narcotics, IV fluids); prophylactic medications indicated for ≤24 hrs	Prolonged (i.e., not rapidly responsive to symptomatic medication and/or brief interruption of infusion); recurrence of symptoms following initial improvement; hospitalization indicated for other clinical sequelae (e.g., renal impairment, pulmonary infiltrates)	Life-threatening; pressor or ventilatory support indicated	Death
REMARK: Cytokine release syndromes/acute infusion reactions are different from Allergic/hypersensitive reactions, although some of the manifestations are common to both AEs. An acute infusion reaction may occur with an agent that causes cytokine release (e.g., monoclonal antibodies or other biological agents). Signs and symptoms usually develop during or shortly after drug infusion and generally resolve completely within 24 hrs of completion of infusion. Signs/symptoms may include: Allergic reaction/hypersensitivity (including drug fever); Arthralgia (joint pain); Bronchospasm; Cough; Dizziness; Dyspnea (shortness of breath); Fatigue (asthenia, lethargy, malaise); Headache; Hypertension; Hypotension; Myalgia (muscle pain); Nausea; Pruritis/itching; Rash/desquamation; Rigors/chills; Sweating (diaphoresis); Tachycardia; Tumor pain (onset or exacerbation of tumor pain due to treatment); Urticaria (hives, welts, wheals); Vomiting.<br>ALSO CONSIDER: Allergic reaction/hypersensitivity (including drug fever); Bronchospasm, wheezing; Dyspnea (shortness of breath); Hypertension; Hypotension; Hypoxia; Prolonged QTc interval; Supraventricular and nodal arrhythmia – *Select*; Ventricular arrhythmia – *Select*.						
NAVIGATION NOTE: Disseminated intravascular coagulation (DIC) is graded in the COAGULATION CATEGORY.						
NAVIGATION NOTE: Fanconi's syndrome is graded as Urinary electrolyte wasting (e.g., Fanconi's syndrome, renal tubular acidosis) in the RENAL/GENITOURINARY CATEGORY.						
Flu-like syndrome	Flu-like syndrome	Symptoms present but not interfering with function	Moderate or causing difficulty performing some ADL	Severe symptoms interfering with ADL	Disabling	Death
REMARK: Flu-like syndrome represents a constellation of symptoms which may include cough with catarrhal symptoms, fever, headache, malaise, myalgia, prostration, and is to be used when the symptoms occur in a cluster consistent with one single pathophysiological process.						
NAVIGATION NOTE: Renal tubular acidosis is graded as Urinary electrolyte wasting (e.g., Fanconi's syndrome, renal tubular acidosis) in the RENAL/GENITOURINARY CATEGORY.						

		Grade				
**Adverse Event**	**Short Name**	**1**	**2**	**3**	**4**	**5**
"Retinoic acid syndrome"	"Retinoic acid syndrome"	Fluid retention; less than 3 kg of weight gain; intervention with fluid restriction and/or diuretics indicated	Mild to moderate signs/ symptoms; steroids indicated	Severe signs/symptoms; hospitalization indicated	Life-threatening; ventilatory support indicated	Death
REMARK: Patients with acute promyelocytic leukemia may experience a syndrome similar to "retinoic acid syndrome" in association with other agents such as arsenic trioxide. The syndrome is usually manifested by otherwise unexplained fever, weight gain, respiratory distress, pulmonary infiltrates and/or pleural effusion, with or without leukocytosis.						
ALSO CONSIDER: Acute vascular leak syndrome; Pleural effusion (non-malignant); Pneumonitis/pulmonary infiltrates.						
NAVIGATION NOTE: SIADH is graded as Neuroendocrine: ADH secretion abnormality (e.g., SIADH or low ADH) in the ENDOCRINE CATEGORY.						
NAVIGATION NOTE: Stevens-Johnson syndrome is graded as Rash: erythema multiforme (e.g., Stevens-Johnson syndrome, toxic epidermal necrolysis) in the DERMATOLOGY/SKIN CATEGORY.						
NAVIGATION NOTE: Thrombotic microangiopathy is graded as Thrombotic microangiopathy (e.g., thrombotic thrombocytopenic purpura [TTP] or hemolytic uremic syndrome [HUS]) in the COAGULATION CATEGORY.						
Tumor flare	Tumor flare	Mild pain not interfering with function	Moderate pain; pain or analgesics interfering with function, but not interfering with ADL	Severe pain; pain or analgesics interfering with function and interfering with ADL	Disabling	Death
REMARK: Tumor flare is characterized by a constellation of signs and symptoms in direct relation to initiation of therapy (e.g., anti-estrogens/androgens or additional hormones). The symptoms/signs include tumor pain, inflammation of visible tumor, hypercalcemia, diffuse bone pain, and other electrolyte disturbances.						
ALSO CONSIDER: Calcium, serum-high (hypercalcemia).						
Tumor lysis syndrome	Tumor lysis syndrome	—	—	Present	—	Death
ALSO CONSIDER: Creatinine; Potassium, serum-high (hyperkalemia).						
Syndromes – Other (Specify, __)	Syndromes – Other (Specify)	Mild	Moderate	Severe	Life-threatening; disabling	Death

VASCULAR

		Grade				
**Adverse Event**	**Short Name**	**1**	**2**	**3**	**4**	**5**
Acute vascular leak syndrome	Acute vascular leak syndrome	—	Symptomatic, fluid support not indicated	Respiratory compromise or fluids indicated	Life-threatening; pressor support or ventilatory support indicated	Death
Peripheral arterial ischemia	Peripheral arterial ischemia	—	Brief (<24 hrs) episode of ischemia managed non-surgically and without permanent deficit	Recurring or prolonged (≥24 hrs) and/or invasive intervention indicated	Life-threatening, disabling and/or associated with end organ damage (e.g., limb loss)	Death
Phlebitis (including superficial thrombosis)	Phlebitis	—	Present	—	—	—
ALSO CONSIDER: Injection site reaction/extravasation changes.						
Portal vein flow	Portal flow	—	Decreased portal vein flow	Reversal/retrograde portal vein flow	—	—
Thrombosis/embolism (vascular access-related)	Thrombosis/embolism (vascular access)	—	Deep vein thrombosis or cardiac thrombosis; intervention (e.g., anticoagulation, lysis, filter, invasive procedure) not indicated	Deep vein thrombosis or cardiac thrombosis; intervention (e.g., anticoagulation, lysis, filter, invasive procedure) indicated	Embolic event including pulmonary embolism or life-threatening thrombus	Death
Thrombosis/thrombus/ embolism	Thrombosis/thrombus/ embolism	—	Deep vein thrombosis or cardiac thrombosis; intervention (e.g., anticoagulation, lysis, filter, invasive procedure) not indicated	Deep vein thrombosis or cardiac thrombosis; intervention (e.g., anticoagulation, lysis, filter, invasive procedure) indicated	Embolic event including pulmonary embolism or life-threatening thrombus	Death
Vessel injury-artery – *Select*:<br>– Aorta<br>– Carotid<br>– Extremity-lower<br>– Extremity-upper<br>– Other NOS<br>– Visceral	Artery injury – *Select*	Asymptomatic diagnostic finding; intervention not indicated	Symptomatic (e.g., claudication); not interfering with ADL; repair or revision not indicated	Symptomatic interfering with ADL; repair or revision indicated	Life-threatening; disabling; evidence of end organ damage (e.g., stroke, MI, organ or limb loss)	Death

NAVIGATION NOTE: Vessel injury to an artery intra-operatively is graded as Intra-operative injury – *Select Organ or Structure* in the SURGERY/INTRA-OPERATIVE INJURY CATEGORY.

		Grade				
**Adverse Event**	**Short Name**	**1**	**2**	**3**	**4**	**5**
Vessel injury-vein – *Select*:<br>– Extremity-lower<br>– Extremity-upper<br>– IVC<br>– Jugular<br>– Other NOS<br>– SVC<br>– Viscera	Vein injury – *Select*	Asymptomatic diagnostic finding; intervention not indicated	Symptomatic (e.g., claudication); not interfering with ADL; repair or revision not indicated	Symptomatic interfering with ADL; repair or revision indicated	Life-threatening; disabling; evidence of end organ damage	Death
NAVIGATION NOTE: Vessel injury to a vein intra-operatively is graded as Intra-operative injury – *Select Organ or Structure* in the SURGERY/INTRA-OPERATIVE INJURY CATEGORY.						
Visceral arterial ischemia (non-myocardial)	Visceral arterial ischemia	—	Brief (<24 hrs) episode of ischemia managed medically and without permanent deficit	Prolonged (≥24 hrs) or recurring symptoms and/or invasive intervention indicated	Life-threatening; disabling; evidence of end organ damage	Death
ALSO CONSIDER: CNS cerebrovascular ischemia.						
Vascular – Other (Specify, __)	Vascular – Other (Specify)	Mild	Moderate	Severe	Life-threatening; disabling	Death
