# Supplementary material for: Characterization of Pharmacologic and Pharmacokinetic Properties of CCX168, a Potent and Selective Orally Administered Complement 5a Receptor Inhibitor, Based on Preclinical Evaluation and Randomized Phase 1 Clinical Study
Source: PLoS One. 2016 Oct 21;11(10):e0164646. doi: 10.1371/journal.pone.0164646 (PMC5074546; doi:10.1371/journal.pone.0164646)
Supplement: S1 Table — (DOCX) [file pone.0164646.s003.docx]

**S1 Table. Selectivity profile towards other chemokine receptors, cytochrome P450 enzymes and hERG patch clamp test.**

| **Human Receptor or Enzyme** | **IC50 (nM)** | **Assay** | **Cells** |
| --- | --- | --- | --- |
| **CCR1** | >10,000 | Migration | THP-1 |
| **CCR2** | >10,000 | Migration | THP-1 |
| **CCR3** | >10,000 | Calcium | HEK 293-CCR3 |
| **CCR4** | >10,000 | Calcium | Activated T cells |
| **CCR5** | 6,700 | Migration | Baf3-CCR5 |
| **CCR6** | >10,000 | Calcium | Activated T cells |
| **CCR7** | >10,000 | Calcium | Activated T cells |
| **CCR8** | >10,000 | Calcium | Activated T cells |
| **CCR9** | >10,000 | Migration | MOLT-4 |
| **CCR10** | 8,000 | Migration | L1.2-CCR10 |
| **CCR12** | >10,000 | Migration | U937 |
| **CXCR1** | >10,000 | Calcium | Neutrophils |
| **CXCR2** | >10,000 | Calcium | Neutrophils |
| **CXCR3** | >10,000 | Calcium | Activated T cells |
| **CXCR4** | >10,000 | Calcium | Activated T cells |
| **CXCR5** | >10,000 | Calcium | L1.2-CXCR5 |
| **CXCR6** | >10,000 | Calcium | Activated T cells |
| **CXCR7** | >10,000 | Binding | MDA-MB 435-CXCR7 |
| **C5L2** | >10,000 | Binding | HEK 293-C5L2 |
| **C3aR** | >10,000 | Calcium | HEK 293-C3aR |
| **ChemR23** | >10,000 | Migration | L1.2-ChemR23 |
| **GPR1** | >10,000 | Binding | HEK 293-GPR1 |
| **FPR1** | >10,000 | Migration | U937 |
| **CYP 1A2** | >10,000 | Enzyme Inhibition | Human liver microsomes |
| **CYP 2C9** | >10,000 | Enzyme Inhibition | Human liver microsomes |
| **CYP 2C19** | >10,000 | Enzyme Inhibition | Human liver microsomes |
| **CYP 2D6** | >10,000 | Enzyme Inhibition | Human liver microsomes |
| **CYP 3A4** | >10,000 | Enzyme Inhibition | Human liver microsomes |
| **hERG** | >5,000 | Patch clamp | HEK 293 hERG |
