# Supplementary material for: Characterization of Pharmacologic and Pharmacokinetic Properties of CCX168, a Potent and Selective Orally Administered Complement 5a Receptor Inhibitor, Based on Preclinical Evaluation and Randomized Phase 1 Clinical Study
Source: PLoS One. 2016 Oct 21;11(10):e0164646. doi: 10.1371/journal.pone.0164646 (PMC5074546; doi:10.1371/journal.pone.0164646)
Supplement: S2 Table — (DOCX) [file pone.0164646.s004.docx]

**S1 Table. CCX168 selectivity profile against a broad panel of targets.***

| **Receptors** | **Inhibition (%) at 10 µM** |
| --- | --- |
| **Adenosine A1** | 5 |
| **Adenosine A2A** | 42 |
| **Adenosine A3** | 33 |
| **Adrenergic alpha 1** | 2 |
| **Adrenergic alpha 2** | -1 |
| **Adrenergic beta 1** | 6 |
| **Adrenergic beta** | 19 |
| **Angiotensin-II AT1** | 9 |
| **BZD (central) (agonist radioligand)** | -14 |
| **Bradykinin B2** | 17 |
| **Cannabinoid CB1** | 18 |
| **Cholecystokinin CCK1 (CCKA)** | -45 |
| **Dopamine D1** | 19 |
| **Dopamine D2S** | 3 |
| **Endothelin ETA** | 1 |
| **GABA** | 14 |
| **Galanin GAL2** | 6 |
| **Chemokine CXCR2** | -11 |
| **Chemokine CCR1** | 4 |
| **Histamine H1** | 7 |
| **Histamine H2** | -36 |
| **Melanocortin MC4** | -5 |
| **Melatonin MT1 (ML1A)** | -8 |
| **Muscarinic M1** | -10 |
| **Muscarinic M2** | -1 |
| **Muscarinic M3** | 12 |
| **Neurokinin NK2** | 12 |
| **Neurokinin NK3** | 12 |
| **Neuropeptide Y1** | 2 |
| **Neuropeptide Y2** | 2 |
| **Neurotensin NTS1 (NT1)** | -8 |
| **Opioid and opioid-like delta 2 (DOP)** | 4 |
| **Opioid and opioid-like kappa (KOP)** | 20 |
| **Opioid and opioid-like mu (MOP)** | 19 |
| **Opioid and opioid-like NOP (ORL1)** | 8 |
| **TP (h) (TXA2/PGH2)** | -1 |
| **Serotonin 5-HT1A** | 6 |
| **Serotonin 5-HT1B** | 12 |
| **Serotonin 5-HT2A** | 4 |
| **Serotonin 5-HT2B** | -5 |
| **Serotonin 5-HT3** | 1 |
| **Serotonin 5-HT5A** | 3 |
| **Serotonin 5-HT6** | 8 |
| **Serotonin 5-HT7** | 9 |
| **Somatostatin sst** | -1 |
| **Vasoactive intestinal peptide VPAC1 (VIP1)** | 1 |
| **Vasopressin V1a** | -2 |
| **Ca2+ channel (L, verapamil site)** | -45 |
| **KV channel** | -2 |
| **SKCa channel (antagonist radioligand)** | -11 |
| **Na^+^ channel (site 2)** | 59 |
| **Cl^-^ channel (GABA-gated)** | 23 |
| **norepinephrine transporter** | 16 |
| **dopamine transporter** | 9 |
| **5-HT transporter** | 5 |

* Assays performed by Cerep, Inc (http://www.cerep.fr; France) as part of their ExpresSProfile service. Data are for a CCX168 concentration of 10,000 nM.
